# Prenatal diagnosis and prevalence of critical congenital heart defects: an international retrospective cohort study

**DOI:** 10.1136/bmjopen-2018-028139

**Published:** 2019-07-02

**Authors:** Marian K Bakker, Jorieke E H Bergman, Sergey Krikov, Emmanuelle Amar, Guido Cocchi, Janet Cragan, Hermien E K de Walle, Miriam Gatt, Boris Groisman, Shiliang Liu, Wendy N Nembhard, Anna Pierini, Anke Rissmann, Shanthi Chidambarathanu, Antonin Sipek Jr, Elena Szabova, Giovanna Tagliabue, David Tucker, Pierpaolo Mastroiacovo, Lorenzo D Botto

**Affiliations:** 1 Department of Genetics, Eurocat registration Northern Netherlands, University of Groningen, University Medical Center Groningen, Groningen, The Netherlands; 2 Division of Medical Genetics, Department of Pediatrics, University of Utah, Salt Lake City, Utah, USA; 3 Registre Des Malformations en Rhone Alpes, REMERA, Lyon, France; 4 Neonatology Unit, S.Orsola-Malpighi Hospital, Department of Medical and Surgical Sciences, University of Bologna, Bologna, Italy; 5 Metropolitan Atlanta Congenital Defects Program, National Center on Birth Defects and Developmental Disabilities, Centers for Disease Control and Prevention, Atlanta, Georgia, USA; 6 Malta Congenital Anomalies Registry, Directorate for Health Information and Research, Malta, Malta; 7 National Network of Congenital Anomalies of Argentina (RENAC), National Center of Medical Genetics, National Ministry of Health, Buenos Aires, Argentina; 8 Maternal, Child and Youth Health Division, Public Health Agency of Canada, Ottawa, Canada; 9 Arkansas Reproductive Health Monitoring System, University of Arkansas for Medical Sciences, Fay W Boozman College of Public Health and the Arkansas Children’s Research Institute, Little Rock, Arkansas, USA; 10 Institute of Clinical Physiology, National Research Council and Fondazione Toscana Gabriele Monasterio, Tuscany Registry of Congenital Defects, Pisa, Italy; 11 Malformation Monitoring Centre, Medical Faculty, Otto von Guericke University, Magdeburg, Germany; 12 Birth Defects Registry of India, Mediscan Systems, Chennai, India; 13 Institute of Medical Biology and Genetics First Faculty of Medicine Charles University and General University Hospital, Prague, Czech Republic; 14 Slovak Teratologic Information Centre (FPH), Slovak Medical University, Bratislava, Slovakia; 15 Lombardy Birth Defects Registry, Fondazione IRCCS Instituto Nazionale Tumori, Milan, Italy; 16 Congenital Anomaly Register and Information Service for Wales, Public Health Wales, Swansea, Wales, UK; 17 International Center on Birth Defects, University of Utah, Salt Lake City, Utah, USA

**Keywords:** critical congenital heart defects, prenatal diagnosis, epidemiology

## Abstract

**Objectives:**

To assess international trends and patterns of prenatal diagnosis of critical congenital heart defects (CCHDs) and their relation to total and live birth CCHD prevalence and mortality.

**Setting:**

Fifteen birth defect surveillance programmes that participate in the International Clearinghouse for Birth Defects Surveillance and Research from 12 countries in Europe, North and South America and Asia.

**Participants:**

Live births, stillbirths and elective terminations of pregnancy for fetal anomaly diagnosed with 1 of 12 selected CCHD, ascertained by the 15 programmes for delivery years 2000 to 2014.

**Results:**

18 243 CCHD cases were reported among 8 847 081 births. The median total prevalence was 19.1 per 10 000 births but varied threefold between programmes from 10.1 to 31.0 per 10 000. CCHD were prenatally detected for at least 50% of the cases in one-third of the programmes. However, prenatal detection varied from 13% in Slovak Republic to 87% in some areas in France. Prenatal detection was consistently high for hypoplastic left heart syndrome (64% overall) and was lowest for total anomalous pulmonary venous return (28% overall). Surveillance programmes in countries that do not legally permit terminations of pregnancy tended to have higher live birth prevalence of CCHD. Most programmes showed an increasing trend in prenatally diagnosed CCHD cases.

**Discussion and conclusions:**

Prenatal detection already accounts for 50% or more of CCHD detected in many programmes and is increasing. Local policies and access likely account for the wide variability of reported occurrence and prenatal diagnosis. Detection rates are high especially for CCHD that are more easily diagnosed on a standard obstetric four-chamber ultrasound or for fetuses that have extracardiac anomalies. These ongoing trends in prenatal diagnosis, potentially in combination with newborn pulse oximetry, are likely to modify the epidemiology and clinical outcomes of CCHD in the near future.

**Strengths and limitations of this study**

- This retrospective cohort study includes a large sample of more than 18 000 cases with critical congenital heart defects from 15 birth defect surveillance programmes from Europe, North and South America and Asia.
- The programmes come from areas with different policies regarding prenatal screening and diagnosis and therefore allow a wider view of factors related to prevalence, ascertainment and prenatal diagnosis.
- The individual case records were centrally reviewed by clinicians with expertise in genetics and paediatric cardiology in order to harmonise diagnoses and clinical classification.
- The quality and completeness of the data depend on the programme’s methods related to data collection, coding and classification.
- Details on the severity of each case were not available.

## Introduction

Congenital heart defects (CHDs) are among the most common birth defects, affecting approximately 1 in 100 births.^1 2^ About 20%–25% of CHD, or about 1 in 500 births, have been described as critical congenital heart defects (CCHDs) because they require urgent and significant medical and surgical care to ensure survival.^1 3^ CCHD represent a significant clinical and public health challenge. In lower income countries, where complex health resources are the scarcest, CCHD are associated with very high mortality. In high-income countries, including North America and Europe, CCHDs are associated with lifelong morbidities and, for healthcare systems, with some of the leading drivers for paediatric in-hospital care costs.^4 5^

Treatment and outcomes of CCHD have improved dramatically over the last decades.^6–9^ A major part of the treatment strategy is to identify CCHD as early as possible, so that a management plan can be agreed on and put in place prior to the baby presenting acutely and often in cardiac failure.^10–14^ Prenatal diagnosis and newborn screening are two such early detection strategies, with prenatal diagnosis allowing for more deliberate management planning with family and care providers.

Prenatal detection of CCHD depends on several factors, including technology (the availability of adequate equipment), sonographer skills (CCHD detection requires more experience than the standard prenatal anatomic scan), screening policies and access to prenatal screening services (location and costs).^15 16^ Because these factors vary by country, within a country, and over time, as services and policies evolve, so will the rate and impact of prenatal diagnosis of CCHD. In turn, the rate of prenatal diagnosis can have multiple consequences on the pattern, trends and outcomes of CCHD in a given population. Through earlier detection, prenatal diagnosis will improve overall ascertainment of CCHD by the time of birth, which could be reflected in more accurate estimates of prevalence at birth by birth registries. This in turn can improve longitudinal population-based surveillance of CCHD-related outcomes through registry or linkage studies. Prenatal detection may also be associated with elective terminations of pregnancy for fetal anomaly (TOPFA), possibly reducing the live birth (LB) prevalence of CCHD and changing the overall pattern of CHD in the population.^17^ Thus, prenatal diagnosis of CCHD has the potential of changing the epidemiology and public health impact of CCHD in complex ways. In this study, we examined the changing trends of prenatal diagnosis of CCHD and their impact on CCHD birth prevalence and mortality in a geographically diverse set of programmes that participate in the International Clearinghouse for Birth Defects Surveillance and Research (ICBDSR).

## Methods

### Study design and contributing programmes

This retrospective cohort study is based on data from 15 birth defect surveillance programmes (table 1) that are members of the ICBDSR. The ICBDSR is an international network of birth defects surveillance and research programmes, whose mission is collaborative surveillance of birth defects and research into their causes and outcomes (www.icbdsr.org). The 15 programmes represent 12 countries from Europe, North America, South America and Asia. Participating programmes had to be able to provide case-level data with specific diagnoses for CHD and extracardiac malformations for at least two birth years. Most contributing programmes are population based, while the remainder are hospital based. The programme from India is hospital based and a solely prenatal programme, meaning that only cases that are prenatally diagnosed within the contributing hospitals are registered within the programme. The other programmes include both prenatally and postnatally diagnosed cases.

**Table 1:** Selected geographic, registration procedure and policy characteristics of participating surveillance programmes, International Clearinghouse for Birth Defects Surveillance and Research (ICBDSR) Critical Congenital Heart Defects (CCHD) Prenatal Diagnosis study 2000–2014

Country	Area	Type of programme*	Ascertainment period	TOPFA legal	Stillbirth definition for study	Birth years contributed to study
UK	Wales	P	18 years	Yes	≥24 WGA	2001–2012
Germany	Saxony Anhalt	P	1 year	Yes	≥500 g	2001–2012
The Netherlands	Northern	P	10 years	Yes	≥24 WGA	2001–2012
France	Rhone Alpes	P	18 years	Yes	≥20 WGA	2006–2012
Italy	Emilia Romagna	P	1 year	Yes	≥20 WGA	2001–2012
Italy	Lombardy	P	6 years	Yes	≥23 WGA	2009–2010
Italy	Tuscany	P	1 year	Yes	≥20 WGA	2001–2012
Malta	National	P	1 year	No	≥22 WGA or >500 g	2001–2012
Czech Republic	National	P	15 years	Yes	≥28 WGA or >1000 g	2000–2013
Slovak Republic	National	P	Hospital discharge	Yes	≥1000 g	2001–2012
Canada	National	P	1 year	Yes	≥20 WGA or >500 g (or >22 WGA if birth weight is unknown)	2004–2014
USA	Arkansas	P	2 years	Yes	≥20 WGA or >350 g	2001–2010
USA	Atlanta	P	6 years	Yes	≥20 WGA	2001–2008
Argentina	National	H	Hospital discharge	No	≥500 g	2013–2014
India	Chennai	H	Prenatal only	Yes	n.a.	2008–2012

*Type of programme: H: hospital based; P: population based.

†Data for Quebec not included (not available).

CCHDs, critical congenital heart defects; n.a, not applicable (live fetuses only, prenatal screening programme); TOPFA, termination of pregnancy for fetal anomaly; WGA, weeks of gestational age.

### Data contributed

The study included cases (LBs, stillbirths (SBs) and TOPFAs, depending on programme) with 1 of 12 types of CCHD: hypoplastic left heart syndrome (HLHS), coarctation of the aorta (COA), aortic valve stenosis (AoS), tetralogy of Fallot (TOF), d-transposition of great arteries (DTGA), double outlet right ventricle, persistent truncus arteriosus (PTA), interrupted aortic arch (IAA), pulmonary valve atresia with intact ventricular septum, tricuspid valve atresia/hypoplastic right heart (TriA/HRH), single ventricle (SV) and total anomalous pulmonary venous return (TAPVR). These CCHDs are identifiable prenatally through ultrasound either by a four-chamber view or an outflow tract view. The programmes review medical records and abstract clinical information including the diagnoses which, depending on local practices, are made by obstetricians or paediatric cardiologist dependings. The diagnoses are coded and classified by trained registry staff. For each case with 1 of the 12 selected CCHD, programmes provided the following key information: type of CCHD (International Classification of Disease, 9th Revision – Clinical Modification (ICD-9-CM) or International Classification of Disease, 10th Revision – Clinical Modification (ICD-10-CM) code plus verbatim description (if available), timing of diagnosis (prenatal versus postnatal), pregnancy outcome (LB, SB and TOPFA), presence of extracardiac anomalies (structural malformations or syndrome diagnoses, as ICD code plus verbatim description) and, for LBs, survival up to 1 year of age. Cases with an end-of-pregnancy date (delivery or termination of pregnancy) between 2000 and 2014 were included in the study. Most programmes provided data for the time period from 2001 to 2012. Italy–Lombardy provided data on 2009 and 2010 and Argentina provided data on birth years 2013–2014. For the years for which they provided cases, programmes also provided corresponding yearly denominator data, including total number of LBs and total number of SBs.

For cases with more than one CCHD diagnoses, one clinical geneticist with specific expertise in paediatric cardiology (LDB) developed a structured hierarchical process to assign a single main CCHD diagnosis (for details, see the appendix in the online supplementary material). In addition, two clinical geneticists (LDB and JEHB) reviewed all cases with extracardiac or syndrome diagnoses to classify the case either as isolated, with multiple congenital anomalies (MCA) or genetic/syndromic. MCA was defined as any combination of congenital anomalies (cardiac plus one or more extracardiac anomalies) without a recognised underlying cause (genetic or teratogenic) and not constituting a sequence.

10.1136/bmjopen-2018-028139.supp1 Supplementary data

Along with case data, programmes also completed a short questionnaire on local practices and policies related to prenatal diagnosis and pregnancy termination. With the exception of Argentina and Malta, termination of pregnancy was legal in the areas covered by contributing programmes (table 1). In all regions covered by the programmes, ultrasound scans are performed as part of standard obstetric care, including a scan around 18–20 weeks. These scans, depending on local healthcare systems, can be free of charge. In the Netherlands, a routine screening programme for congenital anomalies is offered since 2007, while in Argentina, screening is part of standard obstetric care but depends on availability of technology.

### Analyses

The analyses focused on prevalence, time of detection, clinical presentation and survival. Because some programmes contributed considerably more cases than others, and because a main goal of the study was to examine variations across programmes and countries, the findings are presented primarily by programme rather than in the aggregate. We calculated total prevalence and LB prevalence, with 95% CI computed based on the normal distribution. Total prevalence was calculated as total cases (LB+SB+TOPFA) divided by births (LB+SB), expressed per 10 000 births. LB prevalence was calculated as number of live born cases divided by total number of LBs per 10 000 births. For programmes that contributed more than 2 years of data, we examined time trends in total prevalence and used the χ^2^ test for trend. Timing of detection of the CCHD (prenatal vs postnatal) was examined by programme, by CCHD type and by clinical presentation (isolated, MCA and genetic/syndromic). The proportion prenatally diagnosed over time was also examined for trends (χ^2^ test for trend). Analyses were performed in Excel (Microsoft Office Professional plus 2010) and IBM SPSS Statistics for Windows, V.23.0. Each programme has local approved procedures for ethics approval, and because this study was done using deidentified data, no additional ethics committee approval was required.

### Patient and public involvement

No patients were involved in setting the research question or the outcome measures, nor were they involved in developing plans for implementation of the study. No patients were asked to advise on interpretation or writing up of results. There are no plans to disseminate the results of the research to study participants or the relevant patient community.

## Results

### Prevalence

**Figure 1:**
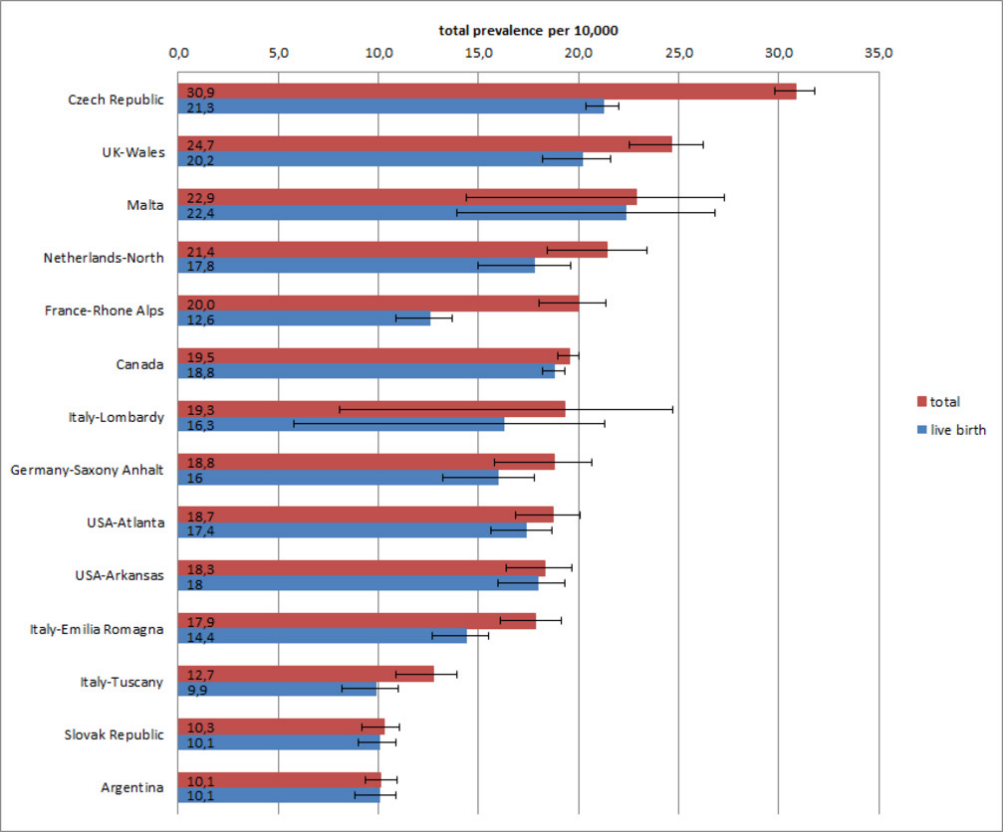
Total prevalence and live birth prevalence (per 10 000 births) with 95% CIs for 12 CCHD types, by programme, International Clearinghouse for Birth Defects Surveillance and Research (ICBDSR) Critical Congenital Heart Defects Prenatal Diagnosis study 2000–2014. ICBDSR programmes, ordered by descending total prevalence, contributed data for different years within this time period (see table 1).Chennai, India programme is not included in prevalence estimates because for this exclusively prenatal programme the denominator data (total births and total live births) are unavailable.

Programmes ascertained 18 243 CCHD cases among 8 847 081 births. The median prevalence was 19.1 per 10 000 births or 1 in 524 births (IQR: 18.2–22.2 per 10 000 births). The highest total prevalence was observed in the Czech Republic (30.9 per 10 000 births) and the lowest in Slovak Republic and Argentina (10.3 and 10.1 per 10 000 births, respectively, table 2 and figure 1). The highest LB prevalence among all programmes was observed in Malta (22.4 per 10 000). During the study period, CCHD showed an increasing trend in total prevalence in France-Rhone Alpes and USA-Arkansas, a decreasing trend in the Czech Republic and USA-Atlanta, and more complex trends in Northern Netherlands and Germany-Saxony Anhalt (online supplementary table S1).

**Table 2:** Total prevalence of CCHD types per 10 000 births, International Clearinghouse for Birth Defects Surveillance and Research (ICBDSR) Critical Congenital Heart Defects (CCHD) Prenatal Diagnosis study 2000–2014*

Programme (by geographic region)	HLHS	COA	AoS	TOF	DTGA	DORV	PTA	IAA	PulmA	TriA/HRH	SV	TAPVR	Total prevalence
UK-Wales	3.3	5.0	2.5	3.5	3.4	1.3	1.1	0.8	1.3	0.5	0.9	1.1	24.7
Germany-Saxony Anhalt	2.7	4.5	1.3	3.3	3.3	0.7	0.6	0.2	0.9	0.3	0.4	0.6	18.8
Netherlands-Northern	3.3	3.6	2.2	3.3	3.7	1.1	0.4	0.4	1.3	0.4	0.9	0.7	21.4
France-Rhone Alpes	4.6	2.2	0.8	3.5	4.0	1.0	0.8	0.1	0.8	0.9	1.1	0.2	20.0
Italy-Lombardy	3.2	4.9	1.1	4.9	1.4	1.4	0.4	0.0	0.7	0.7	0.7	0.0	19.3
Italy-Emilia Romagna	2.6	3.1	0.6	3.8	2.9	1.2	0.7	0.2	0.8	0.8	0.8	0.4	17.9
Italy-Tuscany	2.3	2.0	0.5	2.5	2.7	0.8	0.3	0.1	0.5	0.4	0.5	0.2	12.7
Malta	4.1	4.1	1.2	3.3	4.7	0.6	0.4	0.4	1.2	0.8	1.6	0.2	22.9
Czech Republic	3.3	5.7	4.9	3.8	3.6	3.4	1.3	0.8	1.8	0.6	0.9	0.8	30.9
Slovak Republic	2.3	1.2	0.8	1.8	1.0	0.7	0.9	0.2	0.5	0.3	0.4	0.2	10.3
Canada	1.9	4.9	1.5	3.9	3.0	1.2	0.5	0.1	0.8	0.5	0.4	1.0	19.5
USA-Arkansas	3.2	4.7	2.0	0.9	2.4	1.1	0.6	0.5	0.7	0.5	0.8	1.0	18.3
USA-Atlanta	2.2	4.0	1.1	4.8	2.0	0.5	0.8	0.3	0.6	0.6	0.9	0.7	18.7
Argentina	1.9	1.5	0.3	1.5	1.5	0.5	0.4	0.3	0.3	0.2	1.1	0.5	10.1

*ICBDSR programmes contributed data for different years within this time period (see table 1).

AoS, aortic valve stenosis; CCHD, critical congenital heart defects; COA, coarctation of the aorta; DTGA, d-transposition of great arteries; DORV, double outlet right ventricle; HLHS, hypoplastic left heart syndrome; IAA, interrupted aortic arch; PulmA, pulmonary valve atresia with intact ventricular septum; PTA, persistent truncus arteriosus; SV, single ventricle; TOF, tetralogy of Fallot; TriA/HRH, tricuspid valve atresia/hypoplastic right heart; TAPVR, total anomalous pulmonary venous return.

The difference between total and LB prevalence of CCHD (figure 1) reflected the proportion of TOPFA cases (table 3). The proportion of TOPFA cases varied several-fold in programmes in which TOPFA were legal, from <1% in USA-Arkansas to 24% in the Czech Republic and 35% in France-Rhone Alpes. In Malta and Argentina, termination of pregnancy is not allowed. In India-Chennai, information on the outcome of pregnancy was unavailable in the majority of cases. The proportion of SB CCHD cases was small, on average 2% of total cases, with minor differences among programmes (highest SB proportion of 4% in Northern Netherlands).

**Table 3:** Cases of CCHD by programme and by pregnancy outcome and clinical presentation, International Clearinghouse for Birth Defects Surveillance and Research (ICBDSR) Critical Congenital Heart Defects (CCHD) Prenatal Diagnosis study 2000–2014*

Programme – region	Total cases	Pregnancy outcome (%)				Clinical presentation (%)		
		LB	SB	TOPFA	Unknown	Isolated	MCA	Syndromic
UK-Wales	1 003	81.2	2.5	16.4	0	71.6	15.5	13.0
Germany-Saxony Anhalt	392	84.7	2.0	13.3	0	74.7	14.0	11.2
Netherlands-Northern	477	82.4	4.2	13.4	0	74.8	11.9	13.2
France-Rhone Alps	820	61.7	3.2	35.1	0	70.0	17.6	12.4
Italy-Emilia Romagna	795	79.5	0.1	20.4	0	81.3	9.4	9.3
Italy-Lombardy	55	83.6	3.6	12.7	0	85.5	7.3	7.3
Italy-Tuscany	451	77.2	2.2	20.6	0	90.5	4.7	4.9
Malta	111	97.3	2.7	na	0	79.3	9.0	11.7
Czech Republic	4 569	68.4	0.8	23.6	7.3	89.6	5.8	4.6
Slovak Republic	687	98.1	0.4	1.2	0.3	83.6	10.9	5.5
Canada	6 157	95.2	1.7	3.1	0	79.2	11.6	9.1
USA-Arkansas	722	97.4	2.1	0.4	0.1	67.6	20.2	12.2
USA-Atlanta	796	92.8	2.9	3.4	0.9	67.5	13.7	18.8
Argentina	609	98.4	1.5	na	0.2	75.5	18.4	6.1
India-Chennai†	599	6.8	0.7	35.2	57.3	82.8	15.4	1.8

*ICBDSR programmes contributed data for different years within this time period (see table 1).

†India-Chennai is a prenatal programme, and only includes congenital heart defects that are prenatally diagnosed

CCHD, critical congenital heart defects; LB, live births; MCA, multiple congenital anomalies; na, not available; SB, stillbirths; TOPFA, termination of pregnancy for fetal anomaly.

#### Patterns and distribution of the 12 CCHD types

The total prevalence by CCHD type is presented by programme in table 2. Although the prevalence varied, the proportion of CCHD types was similar among programmes. Five CCHD types—HLHS, CoA and AoS (left ventricular outflow tract obstruction anomalies), TOF and DTGA—accounted for 71% of cases, with some variations among programmes (80% in Lombardy and 56% in India, online supplementary table S2).

#### Prenatal diagnosis

**Figure 2:**
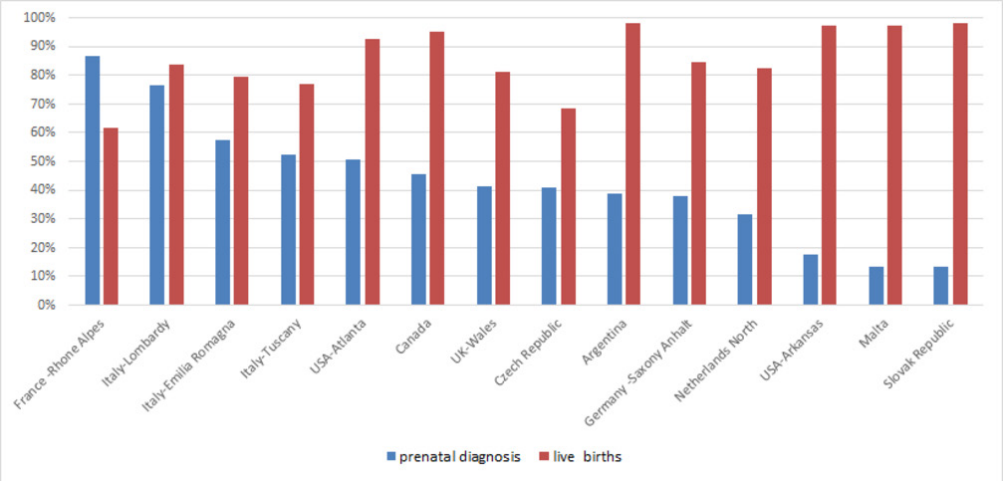
Proportion prenatally diagnosed and proportion of live births among all CCHD cases by programme, International Clearinghouse for Birth Defects Surveillance and Research (ICBDSR) Critical Congenital Heart Defects (CCHD) Prenatal Diagnosis study 2000–2014. ICBDSR Programmes (ordered by descending prenatal diagnosis proportion) contributed data for different years within this time period (see table 1). India-Chennai is not included in the figure because as an exclusively prenatal diagnosis programme, all cases by design were prenatally diagnosed, and information on outcome of pregnancy is missing in the majority of cases.

There was considerable variation in proportion of CCHD identified via prenatal diagnosis among programmes (figure 2) from 87% in France-Rhone Alpes to 13% in Malta and Slovak Republic. In India-Chennai, an exclusively prenatal diagnosis programme, all cases by design were prenatally diagnosed. In programmes with a high proportion of prenatally diagnosed CCHD cases, the proportion of LBs tended to be lower and the proportion of TOPFA higher. The converse was also true: the proportion of LB cases was higher in programmes with a low fraction of prenatally diagnosed cases.

In most programmes, the proportion of CCHD cases prenatally diagnosed increased considerably during the study period, in some cases several-fold (table 4). The proportion prenatally diagnosed also varied by type of CCHD. Such proportion was higher for HLHS and SV, which markedly affect ventricular morphology, and lower for dTGA, TAPVR and AoS, which affect ventricular morphology less markedly or frequently, thereby making prenatal detection more difficult. Among CCHD types, the fraction prenatally diagnosed varied considerably between programmes, but the rank order was similar (table 5). For example, the proportion of HLHS cases prenatally diagnosed varied from 24% in Slovak Republic to 95% in France-Rhone Alpes and 100% in Italy-Lombardy, but within each programme, HLHS was the CCHD diagnosed prenatally most frequently.

**Table 4:** Proportion (%) of prenatally diagnosed CCHD cases by year and result of trend analyses, International Clearinghouse for Birth Defects Surveillance and Research (ICBDSR) Critical Congenital Heart Defects (CCHD) Prenatal Diagnosis study 2000–2014*

Programme by geographic region	2000	2001	2002	2003	2004	2005	2006	2007	2008	2009	2010	2011	2012	2013	2014	Trend
Czech Republic	32.5	35.4	38.8	38.1	40.7	40.8	32.6	51.3	42.9	49.0	50.4	39.8	41.5	20.9		†
UK-Wales		21.9	30.1	32.6	32.9	42.7	38.5	37.1	55.9	40.2	55.6	55.4	50.6			†
Netherlands-Northern		5.3	10.0	17.8	13.6	18.5	31.6	32.4	33.3	44.7	58.3	51.7	65.9			†
France-Rhone Alpes							88.8	85.1	81.0	93.1	92.4	85.2	88.3			-
Canada					43.7	42.5	45.1	43.5	46.7	48.0	45.4	43.5	47.6	46.4	48.1	†
Germany-Saxony Anhalt		40.9	50.0	50.0	39.1	52.6	40.7	55.0	40.0	41.7	40.7	38.7	40.0			-
USA-Atlanta		42.2	41.2	38.1	38.7	48.1	76.7	75.0	66.7							†
USA-Arkansas		23.7	10.3	13.8	10.2	1.9	16.0	18.5	28.9	25.6	19.0					†
Italy-Emilia Romagna		51.1	60.9	64.7	69.6	64.3	67.7	40.6	60.7	53.9	43.8	55.0	61.3			-
Italy-Tuscany		40.0	20.8	35.3	48.6	46.4	50.0	52.8	59.5	55.0	62.5	74.4	73.1			†
Slovac republic		4.3	4.7	7.7	4.7	17.9	14.8	7.5	4.4	23.8	10.8	33.3	20.9			†
Malta				33.3	9.1		12.5	21.4	30.0	36.4		20.0				nc
Italy-Lombardy										75.0	78.3					nc
Argentina														33.5	47.0	nc
India-Chennai									100.0	100.0	100.0	100.0	100.0			nc

*ICBDSR programmes contributed data for different years within this time period, see table 1.

†significant increasing trend, - no trend, nc denotes not calculated because of too few data.

CCHD, critical congenital heart defects.

**Table 5:** Proportion (%) of CCHD prenatally diagnosed, by CCHD type and programme, International Clearinghouse for Birth Defects Surveillance and Research (ICBDSR) Critical Congenital Heart Defects (CCHD) Prenatal Diagnosis study 2000–2014*†

ICBDSR programme by geographic region	Selected CCHD												Overall
	HLHS	SV	PulmA	TriA/HRH	TOF	DTGA	DORV	PTA	IAA	COA	AoS	TAPVR	
France-Rhone Alpes	95.2	100.0	100.0	94.6	84.1	90.2	95.2	70.6	100.0	66.3	61.3	50.0	**86.7**
Italy-Lombardy	100.0	50.0	100.0	100.0	85.7	50.0	75.0	100.0		57.1	66.7		**76.4**
Italy-Emilia Romagna	81.9	68.6	48.6	64.9	50.6	58.6	74.5	75.0	28.6	42.4	28.0	27.8	**57.6**
Italy-Tuscany	84.3	78.9	56.3	71.4	48.9	36.8	82.1	54.5	50.0	25.0	35.3	0.0	**52.3**
USA-Atlanta	77.9	76.9	60.0	70.4	50.7	43.7	63.6	61.1	50.0	34.7	33.3	16.1	**50.5**
Canada	57.8	38.3	52.5	42.0	48.6	34.1	56.0	52.3	50.0	42.4	42.4	46.4	**45.5**
Czech Republic	72.2	59.5	61.4	37.9	29.0	29.9	55.3	53.3	80.7	23.3	30.9	19.5	**40.9**
UK-Wales	88.1	77.1	48.1	66.7	36.8	37.2	57.7	71.1	27.3	17.8	11.8	17.8	**41.5**
Argentina	54.0	55.1	38.1	40.0	36.7	21.6	53.3	29.6	38.9	31.5	25.0	20.0	**38.6**
Germany-Saxony Anhalt	66.1	66.7	50.0	71.4	30.9	33.3	40.0	46.2	40.0	28.0	18.5	25.0	**38.0**
Netherlands-Northern	71.6	63.2	41.4	30.0	24.3	25.6	68.0	11.1	20.0	11.1	4.1	6.7	**31.7**
USA-Arkansas	42.1	32.3	14.8	38.9	14.7	10.4	25.6	36.0	14.3	5.4	2.6	5.1	**17.5**
Malta	25.0	25.0	16.7	25.0	12.5	13.0	33.3	0.0	0.0	0.0	0.0	0.0	**13.5**
Slovak Republic	24.3	10.0	3.2	11.8	6.5	5.8	18.8	21.0	9.1	13.0	5.8	0.0	**13.2**

Programmes are ordered vertically by overall proportion of cases prenatally diagnosed (from high to low), whereas CCHD types are arranged horizontally left to right by approximate ease of prenatal diagnosis by fetal ultrasound (from more easily to less easily detectable on standard four-chamber view).

*ICBDSR programmes contributed data for different years within this time period (see table 1).

†India-Chennai, an exclusively prenatal diagnosis programme, is not included in the table because all cases by design were prenatally diagnosed.

AoS, Artic valve stenosis; CCHD, critical congenital heart defects; COA, coarctation of the aorta, DTGA,  d-transposition of great arteries, DORV,  double outlet right ventricle, HLHS,  hypoplastic left heart syndrome, IAA, interrupted aortic arch, PTA, persistent truncus arteriosus; PulmA, pulmonary valve atresia with intact ventricular septum; SV, single ventricle; TAPVR, total anomalous pulmonary venous return; TOF, tetralogy of Fallot; TriA/HRH, tricuspid valve atresia/hypoplastic right heart.

#### Clinical presentation

**Figure 3:**
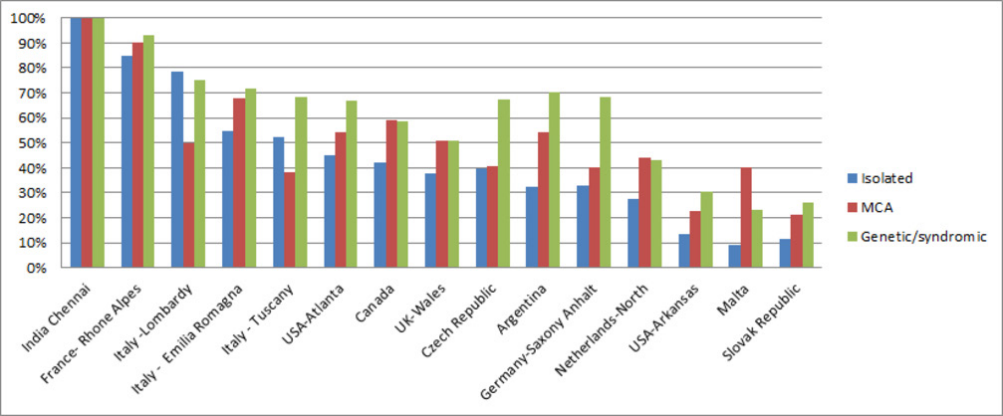
Proportion of prenatally diagnosed CCHD cases according to clinical presentation and by programme International Clearinghouse for Birth Defects Surveillance and Research (ICBDSR) Critical Congenital Heart Defects (CCHD) Prenatal Diagnosis study 2000–2014. Programmes (ordered by descending prenatal detection proportion) contributed data for different years within this time period (see table 1). India-Chennai is a prenatal diagnosis-only programme. MCA, multiple congenital anomalies.

The proportion of prenatally detected cases was higher in syndromic and MCA CCHD cases compared with isolated cases, and the difference was more pronounced in programmes with lower overall prenatal detection proportion (figure 3). Overall, most CCHD present as isolated (80%), with variations between programmes. In Italy-Tuscany and Czech Republic, 90% of the CCHD cases presented as isolated, whereas in USA-Arkansas and USA-Atlanta, 68% presented as isolated (table 2). Some CCHD types were more commonly reported as isolated (AoS, DTGA, TRiA/HRH, HLHS and COA in >80% of the cases) compared with others such as PTA and IAA, which had a higher proportion of syndromic cases (>17% of the cases, data not shown).

#### Mortality in first month of life

**Figure 4:**
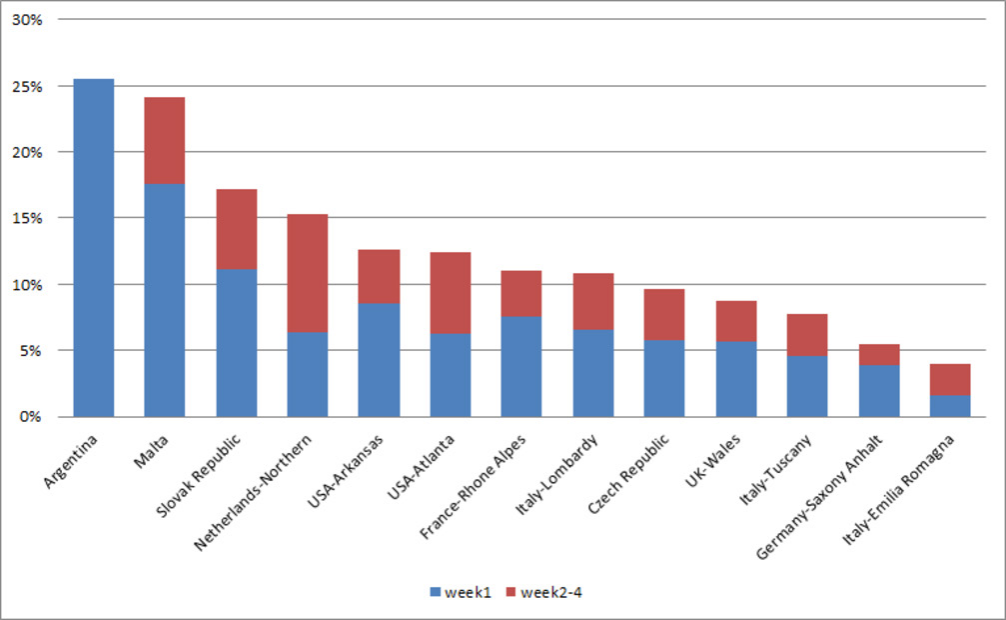
First month mortality in live birth cases with selected CCHD by programme, International Clearinghouse for Birth Defects Surveillance and Research (ICBDSR) Critical Congenital Heart Defects (CCHD) Prenatal Diagnosis study 2000–2014. ICBDSR programmes (ordered by descending first month mortality) contributed data for different years within this time period (see table 1). India-Chennai and Canada are not included in the graph: pregnancy outcomes in India-Chennai are poorly reported and Canada reported on mortality 1 year after birth, not specified in first week or first month mortality.

Because of the variations in follow-up period among programmes, we focused the analysis on neonatal mortality (mortality by the first month of life in LBSs). The highest neonatal mortality was found in Argentina (25.5%) and Malta (24.1%) (figure 4). In these countries, termination of pregnancy is not allowed, and prenatal detection for CCHD is relatively low (table 5 and figure 2). The lowest neonatal mortality was found in Emilia Romagna (4.0%), Germany-Saxony Anhalt (5.4%), Tuscany (7.8%), UK–Wales (8.7%), Czech Republic (9.6%), Italy- Lombardy (10.9%) and France-Rhone Alpes (11.1%). In these programmes, TOPFA proportions are comparatively high (table 2).

## Discussion

In this retrospective cohort study of more than 18 000 CCHD cases from 15 birth defect surveillance programmes from Europe, North and South America and Asia, we observed several remarkable patterns and trends in the occurrence and prenatal diagnosis of CCHD.

First, CCHDs are common regardless of geography and ascertainment programme. The median total prevalence was 19 per 10 000 births, or approximately 1 in 500 births, similar to prior reports.^1 3^ However, total prevalence varied threefold among regions and programmes (figure 1). At least some and perhaps most of such variation is likely related to methodology, that is, the local capacity to detect and report these conditions. Such methodological factors include the ascertainment period after birth, ranging from days to years in the different programmes (table 1), and the ability to obtain a detailed diagnosis, both for the cardiac anomaly and extracardiac findings. For example, programmes reporting the lowest prevalence rates (Slovak Republic and Argentina) have a short postnatal ascertainment period (at birth/hospital discharge). Also, with few exceptions, programmes with low prevalence rates tend to report few syndromic CCHD cases (table 2). A further factor is a programme’s ability to ascertain and record terminations of affected pregnancies (table 2). In countries where terminations of pregnancy are illegal, no terminations are recorded. However, in countries where terminations are legal, a reliable surveillance system may not be able to include these events, and they will be under-reported in these data. Part of the variation in prevalence could reflect true geographical differences in CCHD occurrence due to either genetic predisposition or the frequency of risk factors such as pre-existing maternal diabetes, maternal obesity, use of teratogenic drugs and smoking.^18–22^

A second finding was that, whereas the total prevalence varied considerably among programmes, the relative distribution of CCHD types was similar. For example, HLHS, CoA, TOF and DTGA were consistently among the most prevalent CCHD (online supplementary table S2). The exception was India-Chennai, which deviated from the other programmes likely because of the exclusively prenatal nature of that programme.

A third notable finding was the variation and patterns of prenatal detection (table 5). Although in all regions second trimester scans are offered as part of standard obstetric care, prenatal detection by programme varied from 13% in Slovak Republic to 87% in France-Rhone Alpes, suggesting a role of policies, technical expertise, scanning protocols and practice related to prenatal screening. Even the two programmes in the southeastern USA, Arkansas and Atlanta, Georgia, had widely disparate prenatal detection proportions. The difference in prenatal detection of CCHD between these two programmes is consistent with previous reports, which have shown geographic variations in the USA, ranging from 11.8% to 53.4%.^23^ Prenatal detection was more frequent for clinically complex cases (eg, those with a syndrome or multiple congenital anomalies). This finding, reported also in other studies,^15 24^ likely reflects a greater intensity of fetal examination when any anomaly is identified prenatally. Prenatal detection was also higher for CCHD with primary or significant involvement of the ventricles, such as HLHS and SV, compared with CCHD in which either additional outflow tract views on fetal ultrasound are required (eg, DTGA) or the defects are objectively harder to identify (eg, TAPVR, CoA and AoS). In addition, other studies have suggested that a postnatal diagnosis is more common for CCHD that require a view on fetal ultrasound other than a four-chamber view, lesions that are isolated (eg, absence of another organ system anomaly) or in a setting of poverty or lower population density community.^25^ These findings taken together highlight the crucial role of policies, training and access in driving the rates of prenatal diagnosis in the population.

The proportion of prenatally detected CCHD cases significantly increased over time in most programmes (table 4). The specific patterns varied among programmes. For example, in the Northern Netherlands, a sharp increase in prenatal detection coincided with the introduction of the prenatal screening programme in 2007 (including a 20 week anomaly scan),^26 27^ whereas in other programmes, the increase was more gradual. Increasing trends in prenatal diagnosis were also observed in other studies^23 28–31^ and have been variably attributed to improvements in ultrasound technology as well as policies and recommendations pertaining to examination of the fetal anatomy.^32–34^ For example, in 2006, the International Society for Ultrasound in Obstetrics and Gynaecology issued a guideline that recommended adding the outflow tract view to the basic four-chamber view.^35^

We examined the patterns of prenatal diagnosis in relation to TOPFA proportions. In programmes where such terminations are legal, TOPFA occurred in less than 1%–35% of CCHD cases. Two patterns seemed to emerge. In some programmes such as USA-Arkansas and Slovak Republic, low TOPFA proportions co-occur with a low proportion of prenatal diagnosis, and second, clinically complex cases (eg, associated with other extracardiac anomalies or syndromic cases) seemed to be prenatally diagnosed more often (figure 3), though the relation between clinical complexity and TOPFA was less clear. Pregnancy outcome is not a direct function of prenatal diagnosis. For example, factors that can influence the TOPFA proportion after prenatal diagnosis may be social or cultural (for instance acceptance of TOPFA) and include the legal gestational age limit for pregnancy termination and the extent to which TOPFA are reported to or captured in the healthcare databases.

Finally, neonatal mortality also varied regionally. The study did not specifically assess the system or personal factors potentially associated with such variation, such as gestational age at birth or birth weight. However, we noted that the neonatal mortality was highest in Malta and Argentina where termination of pregnancy is not allowed and prenatal detection of CCHD is low. The lowest neonatal mortality was found in countries where the TOPFA proportions were highest. These findings, though not conclusive, suggest two possibilities. First, prenatal detection might help improve the care of babies with CCHD by allowing for a better plan of care at birth when compared with the unanticipated urgency at birth if no prenatal diagnosis was made.^12 13^ Second, terminations of pregnancy may disproportionately include the anatomically more severe cases (even within the same CCHD type), such that the overall survival is skewed towards what might be only an apparent improvement in outcomes.^36^

### Strengths and weaknesses of the study

The study has several strengths, including the large sample of the CCHD cohort (>18 000 cases) and the systematic nature of case ascertainment whether through population-based or hospital-based programmes. Including programmes from areas with different policies and healthcare systems allowed us a wider view of the inter-related factors that can influence reported prevalence, ascertainment and prenatal diagnosis. Programmes submitted individual case records that were centrally reviewed by clinicians with expertise in genetics (LDB and JEHB) and paediatric cardiology (LDB). This review aimed at harmonising the CCHD diagnoses (eg, cases with more than one CCHD code were systematically assigned a primary diagnosis) as well as the clinical classification as isolated, MCA or syndromic case. The study also has limitations. The quality and completeness of the data submitted centrally depends on the programme’s methods related to data collection, coding and classification (eg, the degree to which clinical staff is involved in these processes). Also, we did not have details on the severity of each CCHD case, which may have contributed to variation across programmes. For example, the clinical presentation of lesions such as AoS and COA can range from mild (eg, not readily identifiable prenatally or clinically at birth) to severe (eg, a truly critical condition in the neonatal period). These variations would influence a programme’s ability to detect these conditions early in life or prenatally and would therefore affect findings such as the total prevalence and the proportion of cases prenatally diagnosed. A last limitation is the challenge and variability in ascertainment of pregnancies that ended in a termination.

### Meaning of the study: possible mechanisms and implications for clinicians or policy makers

Ultimately, these findings, together with prior reports from the literature, have both public health and clinical implications for the care and prevention of CCHD. First, the high prevalence (1 in 500 births) underscores the universal need to address primary prevention and care of CCHD aggressively. Care in particular could be enhanced with earlier diagnosis. In this regard, prenatal diagnosis can complement pulse oximetry newborn screening, and compared with the latter, allow for more time and hence more thoughtful management decisions by well-informed families and clinicians.^37 38^

The increasing trends in prenatal diagnosis rates also highlight the potential for significant changes in the epidemiology and clinical outcomes of CCHD. Although the magnitude of these trends vary in the included programmes, the potential implications are vast. Prenatal diagnosis may continue to influence the reported prevalence at birth as well as the outcomes (eg, morbidity, survival) by a combination of more complete and timely detection and, to a varying extent, its influence on rates of TOPFA. The results of this study demonstrate the value of ongoing surveillance of CCHD in this changing environment.

Tracking and evaluating the patterns of CCHD occurrence is also important in the quest to discover the causes of these severe conditions. For example, in aetiological studies, it is particularly important to include all affected fetuses, as SBs and terminations of pregnancy are more likely to be over-represented in more severe cases. Failing to include such cases would limit the range and possibly skew the findings.

Finally, ongoing monitoring of the CCHD cohort, from pregnancy onwards, is important for researchers to appropriately evaluate long-term outcomes and track the burden of disease on population health.

Important questions remain. Is prenatal diagnosis improving population health? In an era of improving (and often more costly) diagnostic technology, are current systems increasing rather than eliminating potential health disparities? Are we providing the most current information about occurrence and outcomes to clinicians and families for appropriate counselling in the presence of a prenatally detected CCHD? Answering such questions requires a joint effort of epidemiologists and clinicians generating high-quality information and tracking such data over time. Leveraging existing programmes, data sharing and central clinical review and analysis may enhance efficiencies and inform these questions. International networks such as the ICBDSR, the National Birth Defects Prevention Network, and EUROCAT European surveillance of congenital anomalies can help provide the data, the analytic capacity and a long-term vision for sustained, accurate and timely monitoring of the health impact of CCHD, as a basis for interventions aimed at improving primary prevention and care.

## Supplementary Material

Reviewer comments

Author's manuscript
