# Supplementary material for: Prenatal diagnosis and prevalence of critical congenital heart defects: an international retrospective cohort study
Source: BMJ Open. 2019 Jul 2;9(7):e028139. doi: 10.1136/bmjopen-2018-028139 (PMC6609145; doi:10.1136/bmjopen-2018-028139)
Supplement: Supplementary data [file bmjopen-2018-028139supp001.pdf]

Supplementary Table S1. Total prevalence per 10,000 births per year per program for selected CCHD, , International Clearinghouse for Birth Defects Surveillance and Research (ICBDSR) Critical Congenital Heart Defects (CCHD) Prenatal Diagnosis study 2000-2014.[1,2]

Program by geographic region	2000	2001	2002	2003	2004	2005	2006	2007	2008	2009	2010	2011	2012	2013	2014	**Totals**	Trend
Czech Republic	33.0	34.9	32.5	33.7	37.4	29.7	25.6	37.9	33.1	28.8	33.4	30.3	27.3	16.0		**30.9**	\
UK-Wales		23.6	23.9	30.2	21.4	24.9	23.0	30.2	25.8	24.6	22.3	23.1	23.3			**24.7**	-
Malta		30.3	15.3	7.4	28.2	25.9	20.6	35.9	23.7	26.3	9.9	25.5	25.8			**22.9**	-
Netherlands-Northern		19.0	19.6	22.4	30.8	14.6	20.9	20.9	18.3	20.9	27.2	16.8	26.4			**21.4**	~
France-Rhone Alpes							18.9	16.5	16.9	19.6	17.7	23.0	26.6			**20.0**	/
Canada					19.3	20.9	19.2	19.6	19.4	18.8	18.6	20.6	20.4	19.2	19.2	**20.1**	-
Italy-Lombardy										22.1	16.4					**19.3**	nc
Germany-Saxony Anhalt		13.7	19.2	17.0	16.0	25.4	18.2	14.3	28.0	15.1	17.2	19.5	21.8			**18.8**	~
USA-Atlanta		18.9	24.1	19.0	18.0	20.5	18.0	17.0	14.9							**18.7**	\
USA-Arkansas		15.8	20.7	17.1	15.2	13.4	19.6	19.5	18.5	21.5	21.8					**18.3**	/
Italy-Emilia Romagna		18.3	16.5	18.4	18.7	18.5	16.2	15.6	14.3	17.6	17.2	24.5	18.9			**17.9**	-
Italy-Tuscany		15.1	9.0	12.2	12.7	9.8	15.5	11.6	13.8	13.0	18.0	12.8	8.6			**12.7**	-
Slovak Republic		8.9	12.5	10.2	11.9	10.2	10.0	7.3	7.8	10.2	12.7	9.5	12.0			**10.3**	-
Argentina														9.8	10.4	**10.1**	nc

[1]ICBDSR Programs contributed data for different years within this time period, see table 1.

[2]India Chennai program is not included in prevalence estimates because for this exclusively prenatal program the denominator data (total births, total livebirths) are unavailable.

/ significant increasing trend, \ significant decreasing trend, ~heterogeneous prevalence, - no trend, nc not calculated because of too few years

Supplementary Table S2 Distribution of CCHD types per program (%). The proportions add to 100% per program, , International Clearinghouse for Birth Defects Surveillance and Research (ICBDSR) Critical Congenital Heart Defects (CCHD) Prenatal Diagnosis study 2000-2014.[1]

	LVOTO			Conotruncal					RVOTO			
Program by geographic region	HLH	COA	AoS	TOF	DTGA	DORV	PTA	IAA	PulmA	TriA/HRH	SV	TAPVR
UK-Wales	13.5	20.1	10.2	14.4	13.7	5.2	4.5	3.3	5.2	2.1	3.5	4.5
Germany-Saxony Anhalt	14.3	23.7	6.9	17.3	17.6	3.8	3.3	1.3	4.6	1.8	2.3	3.1
Netherlands-Northern	15.5	17.0	10.3	15.5	17.2	5.2	1.9	2.1	6.1	2.1	4.0	3.1
France-Rhone Alpes	22.9	10.9	3.8	17.7	19.9	5.1	4.1	0.4	4.0	4.5	5.5	1.2
Italy-Emilia Romagna	14.6	17.5	3.1	21.1	16.1	6.9	4.0	0.9	4.4	4.7	4.4	2.3
Italy-Lombardy	16.4	25.5	5.5	25.5	7.3	7.3	1.8	0.0	3.6	3.6	3.6	0.0
Italy-Tuscany	18.4	16.0	3.8	19.5	21.1	6.2	2.4	0.4	3.5	3.1	4.2	1.3
Malta	18.0	18.0	5.4	14.4	20.7	2.7	1.8	1.8	5.4	3.6	7.2	0.9
Czech Republic	10.6	18.5	15.8	12.4	11.8	11.0	4.4	2.5	6.0	1.9	2.8	2.5
Slovak Republic	22.1	11.2	7.6	17.9	10.0	7.0	9.0	1.6	4.5	2.5	4.4	2.2
Canada	9.6	25.2	7.5	20.0	15.1	6.2	2.5	0.5	3.9	2.3	1.9	5.2
USA-Arkansas	17.5	25.6	10.7	4.7	13.3	6.0	3.5	2.9	3.7	2.5	4.3	5.4
USA-Atlanta	11.9	21.4	5.7	25.8	10.9	2.8	4.5	1.8	3.1	3.4	4.9	3.9
Argentina	18.6	15.1	2.6	14.8	14.4	4.9	4.4	3.0	3.4	2.5	11.3	4.9
India-Chennai	15.4	1.5	4.0	25.4	10.2	11.5	4.2	0.0	0.8	6.0	19.5	1.5

[1]ICBDSR programs contributed data for different years within this time period, see table 1.
Abbreviations: LVOTO left ventricular outflow tract obstruction, RVOTO right ventricular outflow tract obstruction, SV single ventricle, TAPVR total anomalous pulmonary venous return, CCHD critical congenital heart defects, HLHS hypoplastic left heart syndrome, COA coarctation of the aorta, AoS aortic valve stenosis, TOF tetralogy of Fallot, DTGA d-transposition of great arteries, DORV double outlet right ventricle, PTA persistent truncus arteriosus, IAA interrupted aortic arch, PulmA pulmonary valve atresia with intact ventricular septum, TriA/HRH tricuspid valve atresia / hypoplastic right heart, SV single ventricle, TAPVR total anomalous pulmonary venous return

Appendix. Assigning a main diagnosis of critical congenital heart defects (CCHD)

In this study, programs submitted cases with at least one of 12 diagnoses considered to be consistent with CCHD. These diagnoses (identified by their ICD9 or ICD10 codes) were hypoplastic left heart syndrome (HLHS), coarctation of the aorta (COA), aortic valve stenosis (AoS), tetralogy of Fallot (TOF), d-transposition of great arteries (DTGA), double outlet right ventricle (DORV), persistent truncus arteriosus (PTA), interrupted aortic arch (IAA), pulmonary valve atresia with intact ventricular septum (PulmA), tricuspid valve atresia / hypoplastic right heart (TriA/HRH), single ventricle (SV) and total anomalous pulmonary venous return (TAPVR).

Most cases had just one of these diagnoses ('#CCHD dx' in table below), as noted in the column '# cases'. For the few cases with more than one CCHD code, a single CCHD code was assigned, using the system below for consistency. The rationale for the algorithm was as follows:

a) assign where possible the more severe diagnosis within the same spectrum. For example, in the case of left sided obstructive anomalies, the hierarchy was HLHS > CoA > AoS
b) assign the more dominant condition when diagnoses were not in the same spectrum. For example, in the case of IAA and several other types of CCHD (see below), the diagnosis of IAA prevailed. In the case of HLHS, a CCHD that is both severe clinically as well as easily identifiable at prenatal ultrasound examination, this diagnosis took precedence over several other types of CCHD (see table below). In the case of SV, some CCHD combinations were especially complex, so that the SV group ended up including fairly straightforward conditions such as double inlet left ventricle as well as more complex conditions, in which the SV morphology was joined by several other CCHD lesions.

Two points are worth noting. First, the approach used here was developed by the study's clinical team with expertise in medical genetics and pediatric cardiology. However, some combinations of CCHD codes, there could be disagreements among experts as to which main diagnosis to assign.. Examples include the placement of phenotypes that include tricuspid and pulmonary atresia, or the more complex forms of hypoplastic left heart. Ideally, a more granular approach might be preferable, to avoid grouping somewhat heterogeneous lesions. However, too many small groups would make the analysis unmanageable and a reasonable balance between 'splitting' and 'lumping' had to be achieved. In this case, the decision was made to be systematic (assignment based on specific code combinations) and explicit (full assignment table provided), to improve the clarity and reproducibility of the study. Second, as it is clear from the table, the cases with multiple CCHD codes, and particularly those with more complex combinations, accounted each for very few cases , so any disagreement on the assignment of such cases would likely have a minimal effect of the overall findings of the study.

# CCHD dx	AoS	COA	IAA	DORV	SV	HLHS	PulmA	TriA	TAPVR	DTGA	TOF	PTA	# cases	Final Assignment
1	1	0	0	0	0	0	0	0	0	0	0	0	1792	01.AoS
1	0	1	0	0	0	0	0	0	0	0	0	0	3945	02.COA
2	1	1	0	0	0	0	0	0	0	0	0	0	67	02.COA
1	0	0	1	0	0	0	0	0	0	0	0	0	249	03.IAA
2	0	1	1	0	0	0	0	0	0	0	0	0	7	03.IAA
2	1	0	1	0	0	0	0	0	0	0	0	0	4	03.IAA
2	0	0	1	0	0	0	0	1	0	0	0	0	3	03.IAA
2	0	0	1	0	0	0	0	0	1	0	0	0	2	03.IAA
2	0	0	1	1	0	0	0	0	0	0	0	0	2	03.IAA
3	1	0	1	1	0	0	0	0	0	0	0	0	2	03.IAA
2	0	0	1	0	0	0	0	0	0	0	1	0	1	03.IAA
2	0	0	1	0	0	0	1	0	0	0	0	0	1	03.IAA
3	0	1	1	1	0	0	0	0	0	0	0	0	1	03.IAA
1	0	0	0	1	0	0	0	0	0	0	0	0	1361	04.DORV
2	0	1	0	1	0	0	0	0	0	0	0	0	24	04.DORV
2	0	0	0	1	0	0	0	0	0	0	1	0	9	04.DORV
2	1	0	0	1	0	0	0	0	0	0	0	0	2	04.DORV
1	0	0	0	0	1	0	0	0	0	0	0	0	632	05.SV
2	0	0	0	0	1	0	0	0	0	1	0	0	29	05.SV
2	0	1	0	0	1	0	0	0	0	0	0	0	11	05.SV
2	0	0	0	0	1	0	0	1	0	0	0	0	10	05.SV
2	0	0	0	0	1	0	1	0	0	0	0	0	7	05.SV
3	0	1	0	0	1	0	0	0	0	1	0	0	7	05.SV
2	0	0	0	1	1	0	0	0	0	0	0	0	6	05.SV
2	0	0	0	0	1	0	0	0	0	0	0	1	4	05.SV
2	0	0	0	0	1	0	0	0	1	0	0	0	4	05.SV
2	0	0	1	0	1	0	0	0	0	0	0	0	4	05.SV
2	1	0	0	0	1	0	0	0	0	0	0	0	4	05.SV
3	0	0	0	1	1	0	0	0	0	1	0	0	4	05.SV
3	0	0	0	0	1	0	0	0	1	1	0	0	2	05.SV

2	0	0	0	0	1	0	0	0	0	0	1	0	1	05.SV
3	0	0	0	0	1	0	0	0	0	1	1	0	1	05.SV
3	0	0	0	0	1	0	0	1	0	1	0	0	1	05.SV
3	0	0	0	0	1	0	1	0	0	0	0	1	1	05.SV
3	0	0	0	0	1	0	1	0	0	1	0	0	1	05.SV
3	0	0	0	1	1	0	0	0	1	0	0	0	1	05.SV
3	0	0	0	1	1	0	1	0	0	0	0	0	1	05.SV
3	0	0	1	0	1	0	0	0	0	1	0	0	1	05.SV
3	0	1	0	0	1	0	0	1	0	0	0	0	1	05.SV
3	0	1	0	1	1	0	0	0	0	0	0	0	1	05.SV
3	1	0	0	0	1	0	0	0	0	1	0	0	1	05.SV
4	0	0	0	1	1	0	1	1	0	0	0	0	1	05.SV
4	1	1	0	0	1	0	0	0	0	1	0	0	1	05.SV
1	0	0	0	0	0	1	0	0	0	0	0	0	2386	06.HLH
2	0	1	0	0	0	1	0	0	0	0	0	0	44	06.HLH
2	1	0	0	0	0	1	0	0	0	0	0	0	26	06.HLH
2	0	0	0	1	0	1	0	0	0	0	0	0	18	06.HLH
2	0	0	0	0	0	1	0	0	1	0	0	0	8	06.HLH
2	0	0	0	0	0	1	0	0	0	1	0	0	7	06.HLH
2	0	0	1	0	0	1	0	0	0	0	0	0	6	06.HLH
2	0	0	0	0	1	1	0	0	0	0	0	0	4	06.HLH
3	0	1	0	1	0	1	0	0	0	0	0	0	4	06.HLH
4	0	1	0	1	1	1	0	0	0	0	0	0	3	06.HLH
2	0	0	0	0	0	1	0	0	0	0	0	1	2	06.HLH
2	0	0	0	0	0	1	1	0	0	0	0	0	2	06.HLH
3	0	0	0	1	0	1	0	0	1	0	0	0	2	06.HLH
3	1	1	0	0	0	1	0	0	0	0	0	0	2	06.HLH
2	0	0	0	0	0	1	0	1	0	0	0	0	1	06.HLH
3	0	0	0	1	0	1	0	0	0	1	0	0	1	06.HLH
3	0	0	1	1	0	1	0	0	0	0	0	0	1	06.HLH
3	0	1	0	0	0	1	0	0	1	0	0	0	1	06.HLH
3	0	1	1	0	0	1	0	0	0	0	0	0	1	06.HLH

3	1	0	0	0	1	1	0	0	0	0	0	0	1	06.HLH
4	0	0	0	1	0	1	1	0	0	1	0	0	1	06.HLH
4	0	1	0	0	1	1	0	0	1	0	0	0	1	06.HLH
4	1	1	0	0	0	1	0	1	0	0	0	0	1	06.HLH
4	1	1	0	1	0	1	0	0	0	0	0	0	1	06.HLH
1	0	0	0	0	0	0	1	0	0	0	0	0	1021	07.PulmA
2	0	0	0	1	0	0	1	0	0	0	0	0	9	07.PulmA
2	0	0	0	0	0	0	1	0	1	0	0	0	4	07.PulmA
2	1	0	0	0	0	0	1	0	0	0	0	0	2	07.PulmA
3	0	0	0	1	0	0	1	0	1	0	0	0	1	07.PulmA
4	1	1	0	0	0	0	1	0	1	0	0	0	1	07.PulmA
1	0	0	0	0	0	0	0	1	0	0	0	0	494	08.TriA
2	0	0	0	0	0	0	1	1	0	0	0	0	11	08.TriA
2	1	0	0	0	0	0	0	1	0	0	0	0	4	08.TriA
2	0	0	0	1	0	0	0	1	0	0	0	0	2	08.TriA
2	0	1	0	0	0	0	0	1	0	0	0	0	2	08.TriA
3	0	0	0	0	0	0	1	1	1	0	0	0	1	08.TriA
1	0	0	0	0	0	0	0	0	1	0	0	0	754	09.TAPVR
2	0	0	0	1	0	0	0	0	1	0	0	0	11	09.TAPVR
2	0	1	0	0	0	0	0	0	1	0	0	0	2	09.TAPVR
2	1	0	0	0	0	0	0	0	1	0	0	0	1	09.TAPVR
1	0	0	0	0	0	0	0	0	0	1	0	0	2711	10.DTGA
2	0	1	0	0	0	0	0	0	0	1	0	0	29	10.DTGA
2	0	0	0	1	0	0	0	0	0	1	0	0	15	10.DTGA
2	0	0	0	0	0	0	0	1	0	1	0	0	12	10.DTGA
2	0	0	0	0	0	0	1	0	0	1	0	0	9	10.DTGA
3	0	1	0	1	0	0	0	0	0	1	0	0	8	10.DTGA
3	0	1	0	0	0	0	0	1	0	1	0	0	6	10.DTGA
3	0	0	0	1	0	0	1	0	0	1	0	0	4	10.DTGA
2	0	0	0	0	0	0	0	0	1	1	0	0	3	10.DTGA
3	0	0	0	0	0	0	1	0	1	1	0	0	2	10.DTGA
3	0	0	0	1	0	0	0	0	1	1	0	0	2	10.DTGA

2	1	0	0	0	0	0	0	0	0	1	0	0	1	10.DTGA
3	1	0	0	0	0	0	0	0	0	1	1	0	1	10.DTGA
4	0	1	0	1	0	0	0	1	0	1	0	0	1	10.DTGA
1	0	0	0	0	0	0	0	0	0	0	1	0	3415	11.TOF
2	0	0	0	0	0	0	1	0	0	0	1	0	4	11.TOF
2	0	0	0	0	0	0	0	0	1	0	1	0	3	11.TOF
2	0	1	0	0	0	0	0	0	0	0	1	0	2	11.TOF
2	1	0	0	0	0	0	0	0	0	0	1	0	2	11.TOF
3	0	0	0	1	0	0	0	0	1	0	1	0	2	11.TOF
2	0	0	0	0	0	0	0	1	0	0	1	0	1	11.TOF
1	0	0	0	0	0	0	0	0	0	0	0	1	713	12.PTA
2	0	0	1	0	0	0	0	0	0	0	0	1	13	12.PTA
2	1	0	0	0	0	0	0	0	0	0	0	1	3	12.PTA
2	0	0	0	0	0	0	1	0	0	0	0	1	2	12.PTA
2	0	0	0	0	0	0	0	0	0	1	0	1	1	12.PTA
2	0	0	0	0	0	0	0	0	1	0	0	1	1	12.PTA
3	0	0	0	1	0	0	0	0	1	0	0	1	1	12.PTA
3	0	0	1	0	0	0	0	0	0	0	1	1	1	12.PTA
