# Supplementary material for: Prenatal diagnosis and prevalence of critical congenital heart defects: an international retrospective cohort study
Source: BMJ Open. 2019 Jul 2;9(7):e028139. doi: 10.1136/bmjopen-2018-028139 (PMC6609145; doi:10.1136/bmjopen-2018-028139)
Supplement: Author's manuscript [file bmjopen-2018-028139.draft_revisions.pdf]

BMJ Open

BMJ Open is committed to open peer review. As part of this commitment we make the peer review history of every article we publish publicly available.

When an article is published we post the peer reviewers' comments and the authors' responses online. We also post the versions of the paper that were used during peer review. These are the versions that the peer review comments apply to.

The versions of the paper that follow are the versions that were submitted during the peer review process. They are not the versions of record or the final published versions. They should not be cited or distributed as the published version of this manuscript.

BMJ Open is an open access journal and the full, final, typeset and author-corrected version of record of the manuscript is available on our site with no access controls, subscription charges or pay-per-view fees (http://bmjopen.bmj.com).

If you have any questions on BMJ Open's open peer review process please email info.bmjopen@bmj.com

BMJ Open

Prenatal Diagnosis and Prevalence of Critical Congenital Heart Defects: an International Retrospective Cohort Study

Journal:	*BMJ Open*
Manuscript ID	bmjopen-2018-028139
Article Type:	Research
Date Submitted by the Author:	05-Dec-2018

Complete List of Authors:	Bakker, Marian; University of Groningen,, Department of Genetics, Faculty of Medical Sciences<br>Bergman, Jorieke; University of Groningen,, Department of Genetics, Faculty of Medical Sciences<br>Krikov, Sergey; University of Utah, Division Medical Genetics<br>Amar, Emmanuelle; Registre Des Malformations en Rhone Alpes, REMERA<br>Cocchi, Guido; Neonatology Unit, S.Orsola-Malpighi Hospital, Department of Medical and Surgical Sciences, University of Bologna<br>Cragan, Janet; Metropolitan Atlanta Congenital Defects Program, National Center on Birth Defects and Developmental Disabilities, Centers for Disease Control and Prevention<br>de Walle, Hermien; University of Groningen,, Department of Genetics, Faculty of Medical Sciences<br>Gatt, Miriam; Malta Congenital Anomalies Registry, Directorate for Health Information and Research<br>Groisman, Boris; National Network of Congenital Anomalies of Argentina (RENAC), National Center of Medical Genetics, National Ministry of Health<br>Liu, Shiliang; University of Ottawa/Public Health Agency of Canada,<br>Nembhard, WN; University of Arkansas for Medical Sciences,<br>Pierini, Anna; Institute of Clinical Physiology, National Research Council and Fondazione Toscana Gabriele Monasterio, Tuscany Registry of Congenital Defects<br>Rissmann, Anke; Malformation Monitoring Centre, Medical Faculty, Otto-von-Guericke University<br>Chidambarathanu, S; Birth Defects Registry of India, Mediscan Systems<br>Sipek Jr, Antonin; Institute of Medical Biology and Genetics First Faculty of Medicine Charles University and General University Hospital<br>Szabova, Elena; Slovak Teratologic Information Centre (FPH), Slovak Medical University<br>Tagliabue, Giovanna; Lombardy Birth Defects Registry, Fondazione IRCCS Instituto Nazionale Tumori<br>Tucker, David; Congenital Anomaly Register and Information Service for Wales, Public Health Wales<br>Mastroiacovo, Pierpaolo; International Centre on Birth Defects<br>Botto, Lorenzo; University of Utah, Pediatrics; International Centre on Birth Defects

Keywords:	critical congenital heart defects, Prenatal diagnosis < OBSTETRICS, EPIDEMIOLOGY

SCHOLARONE™
Manuscripts

Prenatal Diagnosis and Prevalence of Critical Congenital Heart Defects: an International Retrospective Cohort Study

MK Bakker[1], JEH Bergman[1], S. Krikov[2], E Amar[3], G Cocchi[4], J Cragan[5], HEK de Walle[1], M Gatt[6], B Groisman[7], S Liu[8], WN Nembhard[9], A Pierini[10], A Rissmann[11], S Chidambarathanu[12], A Sipek Jr[13], E Szabova[14], G Tagliabue[15], D Tucker[16], P Mastroiacovo[17], LD Botto[2,17]

[1]University of Groningen, University Medical Center Groningen, Department of Genetics, Eurocat registration Northern Netherlands, Groningen, The Netherlands
[2] Division of Medical Genetics, Department of Pediatrics, University of Utah, Salt Lake City, USA
[3]Registre Des Malformations en Rhone Alpes, REMERA, Lyon, France
[4] Neonatology Unit, S.Orsola-Malpighi Hospital, Department of Medical and Surgical Sciences, University of Bologna, Bologna, Italy. IMER Registry, Italy
[5] Metropolitan Atlanta Congenital Defects Program, National Center on Birth Defects and Developmental Disabilities, Centers for Disease Control and Prevention, Atlanta, Georgia, USA
[6] Malta Congenital Anomalies Registry, Directorate for Health Information and Research, Malta
[7] National Network of Congenital Anomalies of Argentina (RENAC), National Center of Medical Genetics, National Ministry of Health, Argentina
[8]Maternal, Child and Youth Health Division, Public Health Agency of Canada, Ottawa, Canada
[9] University of Arkansas for Medical Sciences, Fay W Boozman College of Public Health and the Arkansas Children's Research Institute, Arkansas Reproductive Health Monitoring System, Little Rock, Arkansas, USA
[10] Institute of Clinical Physiology, National Research Council and Fondazione Toscana Gabriele Monasterio, Tuscany Registry of Congenital Defects, Pisa, Italy
[11] Malformation Monitoring Centre, Medical Faculty, Otto-von-Guericke University, Magdeburg, Germany
[12] Birth Defects Registry of India, Mediscan Systems, Chennai, India
[13] Institute of Medical Biology and Genetics First Faculty of Medicine Charles University and General University Hospital, Prague, Czech Republic
[14] Slovak Teratologic Information Centre (FPH), Slovak Medical University, Bratislava, Slovak Republic
[15] Lombardy Birth Defects Registry, Fondazione IRCCS Instituto Nazionale Tumori, Milan, Italy
[16] Congenital Anomaly Register and Information Service for Wales, Public Health Wales, Swansea, Wales
[17] International Centre on Birth Defects, Rome, Italy

Corresponding author

Marian K Bakker, University Medical Center Groningen, Department of Genetics, Eurocat Northern Netherlands, PO Box 30001, 9700 RB Groningen, The Netherlands;
m.k.bakker@umcg.nl; +31 50-3617114

For peer review only

For peer review only - http://bmjopen.bmj.com/site/about/guidelines.xhtml

Abstract

Objectives: To assess international trends and patterns of prenatal diagnosis of critical congenital heart defects (CCHD) and their relation to total and live birth CCHD prevalence and mortality

Setting: Fifteen birth defect surveillance programs that participate in the International Clearinghouse for Birth Defect Surveillance and Research (ICBDSR), from 12 countries in Europe, North and South America and Asia.

Participants: Live births, stillbirths, and elective terminations of pregnancy for fetal anomaly diagnosed with one of 12 selected CCHD, ascertained by the 15 programs for delivery years 2000 to 2014.

Results: 18,243 CCHD cases were reported among 8,847,081 births. The median total prevalence was 19.1 per 10,000 births but varied three-fold between programs from 10.1 to 31.0 per 10,000. CCHD were prenatally detected for at least 50% of the cases in one-third of the programs. However, prenatal detection varied from 13% in Slovak Republic to 87% in some areas in France. Prenatal detection was consistently high for hypoplastic left heart syndrome (64% overall), and was lowest for total anomalous pulmonary venous return (28% overall). Surveillance programs in countries that do not legally permit terminations of pregnancy tended to have higher live birth prevalence of CCHD. Most programs showed an increasing trend in prenatally diagnosed CCHD cases.

Discussion and Conclusions: Prenatal detection already accounts for 50% or more of CCHD detected in many programs and is increasing. Local policies and access likely account for the wide variability of reported occurrence and prenatal diagnosis. Detection rates are high especially for CCHD that are more easily diagnosed on a standard obstetric 4-chamber ultrasound, or for fetuses that have extracardiac anomalies. These ongoing trends in prenatal diagnosis, potentially in combination with newborn pulse oximetry, are likely to modify the epidemiology and clinical outcomes of CCHD in the near future.

Trial registration: -

For peer review only - http://bmjopen.bmj.com/site/about/guidelines.xhtml
For peer review only

Strengths and limitations of this study

- This retrospective cohort study includes a large sample of more than 18,000 cases with critical congenital heart defects from 15 birth defect surveillance programs from Europe, North and South America and Asia.
- The programs come from areas with different policies regarding prenatal screening and diagnosis and therefore allow a wider view of factors related to prevalence, ascertainment, and prenatal diagnosis.
- The individual case records were centrally reviewed by clinicians with expertise in genetics and pediatric cardiology in order to harmonize diagnoses and clinical classification.
- The quality and completeness of the data depends on the program's methods related to data collection, coding, and classification.
- Details on the severity of each case were not available.

For peer review only

For peer review only - http://bmjopen.bmj.com/site/about/guidelines.xhtml

Introduction

Congenital heart defects (CHD) are among the most common birth defects, affecting approximately 1 in 100 births [1,2]. About 20 to 25% of CHD, or about 1 in 500 births, have been described as critical congenital heart defects (CCHD) because they require urgent and significant medical and surgical care to ensure survival [1,3]. CCHD represent a significant clinical and public health challenge. In lower-income countries, where complex health resources are the scarcest, CCHD are associated with very high mortality. In high income countries, including North America and Europe, CCHD are associated with lifelong morbidities and, for healthcare systems, with some of the leading drivers for pediatric in-hospital care costs [4,5].

Treatment and outcomes of CCHD have improved dramatically over the last decades [6-9]. A major part of the treatment strategy is to identify CCHD as early as possible, so that a management plan can be agreed upon and put in place prior to the baby presenting acutely and often in cardiac failure [10-14]. Prenatal diagnosis and newborn screening are two such early detection strategies, with prenatal diagnosis allowing for more deliberate management planning with family and care providers.

Prenatal detection of CCHD depends on several factors, including technology (the availability of adequate equipment), sonographer skills (CCHD detection requires more experience than the standard prenatal anatomic scan), screening policies and access to prenatal screening services (location and costs) [15,16]. Because these factors vary by country, within a country, and over time, as services and policies evolve, so will the rate and impact of prenatal diagnosis of CCHD. In turn, the rate of prenatal diagnosis can have multiple consequences on the pattern, trends, and outcomes of CCHD in a given population. Through earlier detection, prenatal diagnosis will improve overall ascertainment of CCHD by the time of birth, which could be reflected in more accurate estimates of prevalence at birth, by birth registries. This in turn can improve longitudinal population-based surveillance of CCHD-related outcomes through registry or linkage studies. Prenatal detection may also be associated with elective terminations of pregnancy for fetal anomaly (TOPFA), possibly reducing the live birth prevalence of CCHD and changing the overall pattern of CHD in the population [17]. Thus, prenatal diagnosis of CCHD has the potential of changing the epidemiology and public health impact of CCHD in complex ways. In this study we examined the changing trends of prenatal diagnosis of CCHD and their impact on CCHD birth prevalence and mortality in a geographically diverse set of programs that participate in the International Clearinghouse for Birth Defect Surveillance and Research (ICBDSR).

For peer review only - http://bmjopen.bmj.com/site/about/guidelines.xhtml

Methods

Study Design and Contributing Programs. This retrospective cohort study is based on data from 15 birth defect surveillance programs (Table 1) that are members of the ICBDSR. The ICBDSR is an international network of birth defects surveillance and research programs, whose mission is collaborative surveillance of birth defects and research into their causes and outcomes [www.icbdsr.org]. The 15 programs represent 12 countries from Europe, North America, South America and Asia**.** Participating programs had to be able to provide case-level data with specific diagnoses for CHD and extracardiac malformations for at least two birth years. Most contributing programs are population-based; the remainder are hospital-based. The program from India is hospital-based and a solely prenatal program, meaning that only cases that are prenatally diagnosed within the contributing hospitals are registered within the program. The other programs include both prenatally and postnatally diagnosed cases.

Data contributed. The study included cases (live births, stillbirths, and TOPFAs, depending on program) with one of 12 types of CCHD: hypoplastic left heart syndrome (HLHS), coarctation of the aorta (COA), aortic valve stenosis (AoS), tetralogy of Fallot (TOF), d-transposition of great arteries (DTGA), double outlet right ventricle (DORV), persistent truncus arteriosus (PTA), interrupted aortic arch (IAA), pulmonary valve atresia with intact ventricular septum (PulmA), tricuspid valve atresia / hypoplastic right heart (TriA/HRH), single ventricle (SV) and total anomalous pulmonary venous return (TAPVR). These CCHD are identifiable prenatally through ultrasound either by a 4-chamber view or an outflow tract view. For each case with one of the 12 selected CCHD, programs provided the following key information: type of CCHD (International Classification of Disease (ICD) 9th revision-Clinical Modification (CM) or ICD-10-CM code plus verbatim description if available), timing of diagnosis (prenatal versus postnatal), pregnancy outcome (live birth (LB), stillbirths (SB), termination of pregnancy for fetal anomaly (TOPFA)), presence of extracardiac anomalies (structural malformations or syndrome diagnoses, as ICD code plus verbatim description), and, for live births, survival up to one year of age. Cases with an end-of-pregnancy date (delivery or termination of pregnancy) between 2000 and 2014 were included in the study. Most programs provided data for the time period from 2001 to 2012. Italy–Lombardy provided data on 2009 and 2010 and Argentina provided data on birth years 2013-2014. For the years for which they provided cases, programs also provided corresponding yearly denominator data, including total number of live births and total number of stillbirths.

For cases with more than one CCHD diagnosis, one clinical geneticist with specific expertise in pediatric cardiology (LDB) developed a structured hierarchical process to assign a single main CCHD

For peer review only - http://bmjopen.bmj.com/site/about/guidelines.xhtml

diagnosis. In addition, two clinical geneticists (LDB and JEHB) reviewed all cases with extracardiac or syndrome diagnoses to classify the case either as isolated, affected with multiple congenital anomalies that are not related (MCA) or genetic/syndromic.

Along with case data, programs also completed a questionnaire on local practices and policies related to prenatal diagnosis and pregnancy termination. With the exception of Argentina and Malta, termination of pregnancy was legal in the areas covered by contributing programs (Table 1). In most regions covered by the programs, a routine prenatal screening program is offered. In the Netherlands a routine screening program is offered since 2007, in Argentina screening is available but not offered routinely and depends on availability of trained staff.

Analyses. The analyses focused on prevalence, time of detection, clinical presentation, and survival. Because some programs contributed considerably more cases than others, and because a main goal of the study was to examine variations across programs and countries, the findings are presented primarily by program rather than in the aggregate. We calculated total prevalence and live birth prevalence, with 95% confidence interval (CI) computed based on the normal distribution. Total prevalence was calculated as total cases (LB + SB + TOPFA) divided by births (LB+SB), expressed per 10,000 births. Live birth prevalence was calculated as number of live born cases divided by total number of live births per 10,000 births. For programs that contributed more than two years of data, we examined time trends in total prevalence, and used the X^2 test for trend. Timing of detection of the CCHD (prenatal versus postnatal) was examined by program, by CCHD type and by clinical presentation (isolated, MCA, genetic/syndromic). The proportion prenatally diagnosed over time was also examined for trends (X^2 test for trend). Analyses were performed in Excel (Microsoft Office Professional plus 2010) and IBM SPSS Statistics for Windows, version 23.0 (Armonk, NY: IBM Corp). Each program has local approved procedures for ethics approval, and because this study was done using de-identified data no additional ethics committee approval was required.

For peer review only - http://bmjopen.bmj.com/site/about/guidelines.xhtml

Table 1. Selected geographic, registration procedure, and policy characteristics of participating surveillance programs, , International Clearinghouse for Birth Defects Surveillance and Research (ICBDSR) Critical Congenital Heart Defects (CCHD) Prenatal Diagnosis study 2000-2014.

Country	Area	Type of program[1]	Ascertainment period	TOPFA legal	Stillbirth definition for study	Birth Years contributed to study
UK	Wales	P	18 years	Yes	$\geq$ 24 WGA	2001-2012
Germany	Saxony Anhalt	P	1 year	Yes	$\geq$ 500g	2001-2012
Netherlands	Northern	P	10 years	Yes	$\geq$ 24 WGA	2001-2012
France	Rhone Alpes	P	18 years	Yes	$\geq$ 20 WGA	2006-2012
Italy	Emilia Romagna	P	1 year	Yes	$\geq$ 20 WGA	2001-2012
Italy	Lombardy	P	6 years	Yes	$\geq$ 23 WGA	2009-2010
Italy	Tuscany	P	1 year	Yes	$\geq$ 20 WGA	2001-2012
Malta	National	P	1 year	**No**	$\geq$ 22 WGA or >500g	2001-2012
Czech Republic	National	P	15 years	Yes	$\geq$ 28 WGA or $\geq$1000g	2000-2013
Slovak Republic	National	P	hospital discharge	Yes	$\geq$1000g	2001-2012
Canada	National	P	1 year	Yes	$\geq$ 20 WGA or >500g (or $\geq$22 WGA if birth weight is unknown)	2004-2014
USA	Arkansas	P	2 years	Yes	$\geq$ 20 WGA or >350g	2001-2010
USA	Atlanta	P	6 years	Yes	$\geq$ 20 WGA	2001-2008
Argentina	National	H	hospital discharge	**No**	$\geq$500g	2013-2014
India	Chennai	H	prenatal only	Yes	n.a.	2008-2012

[1]Type of program: H –hospital-based, P-population based

[2]Data for Quebec not included (not available)

Abbreviations: CCHD-- critical congenital heart defects, TOPFA-- termination of pregnancy for fetal anomaly, WGA-- weeks of gestational age, n.a.-- not applicable (live fetuses only, prenatal screening program)

For peer review only

For peer review only - http://bmjopen.bmj.com/site/about/guidelines.xhtml

Results

Prevalence. Programs ascertained 18,243 CCHD cases among 8,847,081 births. The median prevalence was 19.1 per 10,000 births or 1 in 524 births (inter quartile range (IQR): 18.2-22.2 per 10,000 births). The highest total prevalence was observed in the Czech Republic (30.9 per 10,000 births) and the lowest in Slovak Republic and Argentina (10.3 and 10.1 per 10,000 births respectively, Table 2 and Figure 1). The highest live birth prevalence among all programs was observed in Malta (22.4 per 10,000). During the study period, CCHD showed an increasing trend in total prevalence in France-Rhone Alpes and USA-Arkansas, a decreasing trend in the Czech Republic and USA-Atlanta, and more complex trends in Northern Netherlands and Germany-Saxony Anhalt (Supplementary Table S1).

The difference between total and live birth prevalence of CCHD reflected the proportion of TOPFA cases (Table 3). The proportion of TOPFA cases varied several-fold in programs in which TOPFA were legal, from <1% in USA-Arkansas to 24% in the Czech Republic and 35% in France-Rhone Alpes. In Malta and Argentina termination of pregnancy is not allowed. In India Chennai, information on the outcome of pregnancy was unavailable in the majority of cases. The proportion of stillbirth CCHD cases was small, on average 2% of total cases, with minor differences among programs (highest SB proportion of 4% in Northern Netherlands).

Patterns and distribution of the 12 CCHD types. The total prevalence by CCHD type is presented per program in Table 3. Although the prevalence varied, the proportion of CCHD types was similar among programs. Five CCHD types - HLHS, CoA and AoS (left ventricular outflow tract obstruction anomalies), TOF, and DTGA - accounted for 71% of cases, with some variations among programs (80% in Lombardy and 56% in India, Supplementary Table S2).

Prenatal diagnosis. There was considerable variation in proportion of CCHD identified via prenatal diagnosis among programs (Figure 2), from 87% in France-Rhone Alpes to 13% in Malta and Slovak Republic. In India-Chennai, an exclusively prenatal diagnosis program, all cases by design were prenatally diagnosed. In programs with a high proportion of prenatally diagnosed CCHD cases, the proportion of live births tended to be lower and the proportion of TOPFA higher. The converse was also true: the proportion of live birth cases was higher in programs with a low fraction of prenatally diagnosed cases.

In most programs, the proportion of CCHD cases prenatally diagnosed increased considerably during the study period, in some cases several-fold (Table 4). The proportion prenatally diagnosed also varied by type of CCHD. Such proportion was higher for HLHS and SV, which markedly affect

For peer review only - http://bmjopen.bmj.com/site/about/guidelines.xhtml

ventricular morphology, and lower for dTGA, TAPVR, and AoS, which affect ventricular morphology less markedly or frequently, thereby making prenatal detection more difficult. Among CCHD types, the fraction prenatally diagnosed varied considerably between programs but the rank order was similar (Table 5). For example, the proportion of HLHS cases prenatally diagnosed varied from 24% in Slovak Republic to 95% in France-Rhone Alpes and 100% in Italy-Lombardy, but within each program HLHS was the CCHD diagnosed prenatally most frequently.

Clinical presentation. The proportion of prenatally detected cases was higher in syndromic and MCA CCHD cases compared to isolated cases, and the difference was more pronounced in programs with lower overall prenatal detection proportion (Figure 3). Overall, most CCHD present as isolated (80%), with variations between programs. In Italy-Tuscany and Czech Republic 90% of the CCHD cases presented as isolated whereas in USA-Arkansas and USA-Atlanta 68% presented as isolated (Table 2). Some CCHD types were more commonly reported as isolated (AoS, DTGA, TRiA/HRH, HLHS and COA in >80% of the cases) compared to others such as PTA and IAA, which had a higher proportion of syndromic cases (> 17% of the cases, data not shown).

Mortality in first month of life. Because of the variations in follow-up period among programs, we focused the analysis on neonatal mortality (mortality by the first month of life in live births). The highest neonatal mortality was found in Argentina (25.5%) and Malta (24.1%) (Figure 4). In these countries, termination of pregnancy is not allowed and prenatal detection for CCHD is relatively low (Table 5 and Figure 2). The lowest neonatal mortality was found in Emilia Romagna (4.0%), Germany-Saxony Anhalt (5.4%), Tuscany (7.8%), UK–Wales (8.7%), Czech Republic (9.6%), Italy- Lombardy (10.9%) and France-Rhone Alpes (11.1%). In these programs, TOPFA proportions are comparatively high (Table 2).

For peer review only

For peer review only - http://bmjopen.bmj.com/site/about/guidelines.xhtml

Table 2 – Total prevalence of CCHD types per 10,000 births, , International Clearinghouse for Birth Defects Surveillance and Research (ICBDSR) Critical Congenital Heart Defects (CCHD) Prenatal Diagnosis study 2000-2014.[1]

Program (by geographic region)	HLHS	COA	AoS	TOF	DTGA	DORV	PTA	IAA	PulmA	TriA/ HRH	SV	TAPVR	Total prevalence
UK-Wales	3.3	5.0	2.5	3.5	3.4	1.3	1.1	0.8	1.3	0.5	0.9	1.1	24.7
Germany-Saxony Anhalt	2.7	4.5	1.3	3.3	3.3	0.7	0.6	0.2	0.9	0.3	0.4	0.6	18.8
Netherlands-Northern	3.3	3.6	2.2	3.3	3.7	1.1	0.4	0.4	1.3	0.4	0.9	0.7	21.4
France-Rhone Alpes	4.6	2.2	0.8	3.5	4.0	1.0	0.8	0.1	0.8	0.9	1.1	0.2	20.0
Italy-Lombardy	3.2	4.9	1.1	4.9	1.4	1.4	0.4	0.0	0.7	0.7	0.7	0.0	19.3
Italy-Emilia Romagna	2.6	3.1	0.6	3.8	2.9	1.2	0.7	0.2	0.8	0.8	0.8	0.4	17.9
Italy-Tuscany	2.3	2.0	0.5	2.5	2.7	0.8	0.3	0.1	0.5	0.4	0.5	0.2	12.7
Malta	4.1	4.1	1.2	3.3	4.7	0.6	0.4	0.4	1.2	0.8	1.6	0.2	22.9
Czech Republic	3.3	5.7	4.9	3.8	3.6	3.4	1.3	0.8	1.8	0.6	0.9	0.8	30.9
Slovak Republic	2.3	1.2	0.8	1.8	1.0	0.7	0.9	0.2	0.5	0.3	0.4	0.2	10.3
Canada	1.9	4.9	1.5	3.9	3.0	1.2	0.5	0.1	0.8	0.5	0.4	1.0	19.5
USA-Arkansas	3.2	4.7	2.0	0.9	2.4	1.1	0.6	0.5	0.7	0.5	0.8	1.0	18.3
USA-Atlanta	2.2	4.0	1.1	4.8	2.0	0.5	0.8	0.3	0.6	0.6	0.9	0.7	18.7
Argentina	1.9	1.5	0.3	1.5	1.5	0.5	0.4	0.3	0.3	0.2	1.1	0.5	10.1

[1] ICBDSR programs contributed data for different years within this time period, see table 1.

Abbreviations: CCHD-- critical congenital heart defects, HLHS-- hypoplastic left heart syndrome, COA-- coarctation of the aorta, AoS-- aortic valve stenosis, TOF-- tetralogy of Fallot, DTGA-- d-transposition of great arteries, DORV-- double outlet right ventricle, PTA-- persistent truncus arteriosus, IAA-- interrupted aortic arch, PulmA-- pulmonary valve atresia with intact ventricular septum, TriA/HRH-- tricuspid valve atresia / hypoplastic right heart, SV-- single ventricle TAPVR-- total anomalous pulmonary venous return

For peer review only

For peer review only - http://bmjopen.bmj.com/site/about/guidelines.xhtml

Table 3 Cases of CCHD by program and by pregnancy outcome and clinical presentation, , International Clearinghouse for Birth Defects Surveillance and Research (ICBDSR) Critical Congenital Heart Defects (CCHD) Prenatal Diagnosis study 2000-2014.[1]

		Pregnancy outcome				Clinical presentation		
**Program - region**	**Total cases**	**LB**	**SB**	**TOPFA**	**Unknown**	**Isolated**	**MCA**	**syndromic**
UK-Wales	1 003	81.2%	2.5%	16.4%	0	71.6%	15.5%	13.0%
Germany-Saxony Anhalt	392	84.7%	2.0%	13.3%	0	74.7%	14.0%	11.2%
Netherlands-Northern	477	82.4%	4.2%	13.4%	0	74.8%	11.9%	13.2%
France-Rhone Alps	820	61.7%	3.2%	35.1%	0	70.0%	17.6%	12.4%
Italy-Emilia Romagna	795	79.5%	0.1%	20.4%	0	81.3%	9.4%	9.3%
Italy-Lombardy	55	83.6%	3.6%	12.7%	0	85.5%	7.3%	7.3%
Italy-Tuscany	451	77.2%	2.2%	20.6%	0	90.5%	4.7%	4.9%
Malta	111	97.3%	2.7%	na	0	79.3%	9.0%	11.7%
Czech Republic	4 569	68.4%	0.8%	23.6%	7.3%	89.6%	5.8%	4.6%
Slovak Republic	687	98.1%	0.4%	1.2%	0.3%	83.6%	10.9%	5.5%
Canada	6 157	95.2%	1.7%	3.1%	0	79.2%	11.6%	9.1%
USA-Arkansas	722	97.4%	2.1%	0.4%	0.1%	67.6%	20.2%	12.2%
USA-Atlanta	796	92.8%	2.9%	3.4%	0.9%	67.5%	13.7%	18.8%
Argentina	609	98.4%	1.5%	na	0.2%	75.5%	18.4%	6.1%
India-Chennai[2]	599	6.8%	0.7%	35.2%	57.3%	82.8%	15.4%	1.8%

[1] ICBDSR programs contributed data for different years within this time period, see Table 1.

[2] India-Chennai is a prenatal program, and only includes congenital heart defects that are prenatally diagnosed

Abbreviations: CCHD critical congenital heart defects, LB live births, SB stillbirths, TOPFA termination of pregnancy for fetal anomaly, MCA multiple congenital anomalies, na not available

For peer review only - http://bmjopen.bmj.com/site/about/guidelines.xhtml

Table 4 Proportion (%) of prenatally diagnosed CCHD cases by year and result of trend analyses, , International Clearinghouse for Birth Defects Surveillance and Research (ICBDSR) Critical Congenital Heart Defects (CCHD) Prenatal Diagnosis study 2000-2014.[1]

Program by geographic region	2000	2001	2002	2003	2004	2005	2006	2007	2008	2009	2010	2011	2012	2013	2014	Trend
Czech Republic	32.5	35.4	38.8	38.1	40.7	40.8	32.6	51.3	42.9	49.0	50.4	39.8	41.5	20.9		/
UK-Wales		21.9	30.1	32.6	32.9	42.7	38.5	37.1	55.9	40.2	55.6	55.4	50.6			/
Netherlands-Northern		5.3	10.0	17.8	13.6	18.5	31.6	32.4	33.3	44.7	58.3	51.7	65.9			/
France-Rhone Alpes							88.8	85.1	81.0	93.1	92.4	85.2	88.3			-
Canada					43.7	42.5	45.1	43.5	46.7	48.0	45.4	43.5	47.6	46.4	48.1	/
Germany-Saxony Anhalt		40.9	50.0	50.0	39.1	52.6	40.7	55.0	40.0	41.7	40.7	38.7	40.0			-
USA-Atlanta		42.2	41.2	38.1	38.7	48.1	76.7	75.0	66.7							/
USA-Arkansas		23.7	10.3	13.8	10.2	1.9	16.0	18.5	28.9	25.6	19.0					/
Italy-Emilia Romagna		51.1	60.9	64.7	69.6	64.3	67.7	40.6	60.7	53.9	43.8	55.0	61.3			-
Italy-Tuscany		40.0	20.8	35.3	48.6	46.4	50.0	52.8	59.5	55.0	62.5	74.4	73.1			/
Slovac republic		4.3	4.7	7.7	4.7	17.9	14.8	7.5	4.4	23.8	10.8	33.3	20.9			/
Malta				33.3	9.1		12.5	21.4	30.0	36.4		20.0				nc
Italy-Lombardy										75.0	78.3					nc
Argentina														33.5	47.0	nc
India-Chennai									100.0	100.0	100.0	100.0	100.0			nc

[1] ICBDSR programs contributed data for different years within this time period, see table 1.

/ significant increasing trend, - no trend, nc denotes not calculated because of too few data

Abbreviations: CCHD critical congenital heart defects

For peer review only

For peer review only - http://bmjopen.bmj.com/site/about/guidelines.xhtml

Table 5 – Proportion (%) of CCHD prenatally diagnosed, by CCHD type and program, , International Clearinghouse for Birth Defects Surveillance and Research (ICBDSR) Critical Congenital Heart Defects (CCHD) Prenatal Diagnosis study 2000-2014.[1,2]

	Selected CCHD												
**ICBDSR Program by geographic region**	**HLHS**	**SV**	**PulmA**	**TriA/ HRH**	**TOF**	**DTGA**	**DORV**	**PTA**	**IAA**	**COA**	**AoS**	**TAPVR**	**overall**
France- Rhone Alpes	95.2	100.0	100.0	94.6	84.1	90.2	95.2	70.6	100.0	66.3	61.3	50.0	**86.7**
Italy-Lombardy	100.0	50.0	100.0	100.0	85.7	50.0	75.0	100.0		57.1	66.7		**76.4**
Italy-Emilia Romagna	81.9	68.6	48.6	64.9	50.6	58.6	74.5	75.0	28.6	42.4	28.0	27.8	**57.6**
Italy-Tuscany	84.3	78.9	56.3	71.4	48.9	36.8	82.1	54.5	50.0	25.0	35.3	0.0	**52.3**
USA-Atlanta	77.9	76.9	60.0	70.4	50.7	43.7	63.6	61.1	50.0	34.7	33.3	16.1	**50.5**
Canada	57.8	38.3	52.5	42.0	48.6	34.1	56.0	52.3	50.0	42.4	42.4	46.4	**45.5**
Czech Republic	72.2	59.5	61.4	37.9	29.0	29.9	55.3	53.3	80.7	23.3	30.9	19.5	**40.9**
UK-Wales	88.1	77.1	48.1	66.7	36.8	37.2	57.7	71.1	27.3	17.8	11.8	17.8	**41.5**
Argentina	54.0	55.1	38.1	40.0	36.7	21.6	53.3	29.6	38.9	31.5	25.0	20.0	**38.6**
Germany-Saxony Anhalt	66.1	66.7	50.0	71.4	30.9	33.3	40.0	46.2	40.0	28.0	18.5	25.0	**38.0**
Netherlands-Northern	71.6	63.2	41.4	30.0	24.3	25.6	68.0	11.1	20.0	11.1	4.1	6.7	**31.7**
USA-Arkansas	42.1	32.3	14.8	38.9	14.7	10.4	25.6	36.0	14.3	5.4	2.6	5.1	**17.5**
Malta	25.0	25.0	16.7	25.0	12.5	13.0	33.3	0.0	0.0	0.0	0.0	0.0	**13.5**
Slovak Republic	24.3	10.0	3.2	11.8	6.5	5.8	18.8	21.0	9.1	13.0	5.8	0.0	**13.2**

Note: programs are ordered vertically by overall proportion of cases prenatally diagnosed (from high to low), whereas CCHD types are arranged horizontally left to right by approximate ease of prenatal diagnosis by fetal ultrasound (from more easily to less easily detectable on standard 4-chamber view).

[1] ICBDSR programs contributed data for different years within this time period, see table 1.

[2] India Chennai, an exclusively prenatal diagnosis program, is not included in the table because all cases by design were prenatally diagnosed.

Abbreviations: CCHD-- critical congenital heart defects, HLHS-- hypoplastic left heart syndrome, COA-- coarctation of the aorta, AoS-- aortic valve stenosis, TOF-- tetralogy of Fallot, DTGA-- d-transposition of great arteries, DORV-- double outlet right ventricle, PTA-- persistent truncus arteriosus, IAA-- interrupted aortic arch, PulmA-- pulmonary valve atresia with intact ventricular septum, TriA/HRH-- tricuspid valve atresia / hypoplastic right heart, SV-- single ventricle TAPVR-- total anomalous pulmonary venous return

For peer review only

For peer review only - http://bmjopen.bmj.com/site/about/guidelines.xhtml

Discussion

In this retrospective cohort study of more than 18,000 CCHD cases from 15 birth defect surveillance programs from Europe, North and South America and Asia, we observed several remarkable patterns and trends in the occurrence and prenatal diagnosis of CCHD.

First, CCHD are common regardless of geography and ascertainment program. The median total prevalence was 19 per 10,000 births, or approximately 1 in 500 births, similar to prior reports [1,3]. However, total prevalence varied three-fold among regions and programs (Figure 1). At least some and perhaps most of such variation is likely related to methodology, i.e., the local capacity to detect and report these conditions. Such methodologic factors include the ascertainment period after birth, ranging from days to years in the different programs (Table 1), and the ability to obtain a detailed diagnosis, both for the cardiac anomaly and extracardiac findings. For example, programs reporting the lowest prevalence rates (Slovak Republic and Argentina) have a short postnatal ascertainment period (at birth/ hospital discharge). Also, with few exceptions, programs with low prevalence rates tend to report few syndromic CCHD cases (Table 2). A further factor is a program's ability to ascertain and record terminations of affected pregnancies (Table 2). In countries where terminations of pregnancy are illegal, no terminations are recorded. However, in countries where terminations are legal, a reliable surveillance system may not be able to include these events and they will be underreported in these data. Part of the variation in prevalence could reflect true geographical differences in CCHD occurrence due to either genetic predisposition or the frequency of risk factors such as pre-existing maternal diabetes, maternal obesity, use of teratogenic drugs and smoking [18-22]

A second finding was that, whereas the total prevalence varied considerably among programs, the relative distribution of CCHD types was similar. For example, HLHS, CoA, TOF and DTGA were consistently among the most prevalent CCHD (Supplementary Table S2). The exception was India-Chennai, which deviated from the other programs likely because of the exclusively prenatal nature of that program.

A third notable finding was the variation and patterns of prenatal detection (Table 5). Regionally, prenatal detection by program varied from 13% in Slovak Republic to 87% in France- Rhone Alpes, suggesting a role of policies, technical expertise, and practice related to prenatal screening. Even the two programs in the southeastern U.S., Arkansas and Atlanta, Georgia, had widely disparate prenatal detection proportions. The difference in prenatal detection of CCHD between these two programs is consistent with previous reports, which have shown geographic variations in the Unites States, ranging from 11.8% to 53.4% [23]. Prenatal detection was more frequent for clinically complex cases

For peer review only - http://bmjopen.bmj.com/site/about/guidelines.xhtml

(e.g., those with a syndrome or multiple congenital anomalies). This finding, reported also in other studies [15,24], likely reflects a greater intensity of fetal examination when any anomaly is identified prenatally. Prenatal detection was also higher for CCHD with primary or significant involvement of the ventricles, such as HLHS and SV, compared to CCHD in which either additional outflow tract views on fetal ultrasound are required (e.g., DTGA), or the defects are objectively harder to identify (e.g., TAPVR, CoA, and AoS). In addition, other studies have suggested that a postnatal diagnosis is more common for CCHD that require a view on fetal ultrasound other than a 4-chamber view, lesions that are isolated (e.g., absence of another organ system anomaly) or in a setting of poverty or lower population density community [25]. These findings taken together highlight the crucial role of policies, training, and access in driving the rates of prenatal diagnosis in the population.

The proportion of prenatally detected CCHD cases significantly increased over time in most programs (Table 4). The specific patterns varied among programs. For example, in the Northern Netherlands a sharp increase in prenatal detection coincided with the introduction of the prenatal screening program in 2007 (including a 20 week anomaly scan) [26,27], whereas in other programs the increase was more gradual. Increasing trends in prenatal diagnosis were also observed in other studies [23,28-30] and have been variably attributed to improvements in ultrasound technology as well as policies and recommendations pertaining to examination of the fetal anatomy [31-33]. For example, in 2006 the International Society for Ultrasound in Obstetrics and Gynaecology issued a guideline that recommended adding the outflow tract view to the basic 4-chamber view [34].

We examined the patterns of prenatal diagnosis in relation to TOPFA proportions. In programs where such terminations are legal, TOPFA occurred in less than 1 to 35% of CCHD cases. Two patterns seemed to emerge. In some programs such as USA-Arkansas and Slovak Republic, low TOPFA proportions co-occur with a low proportion of prenatal diagnosis. And second, clinically complex cases (e.g., associated with other extracardiac anomalies or syndromic cases) seemed to be prenatally diagnosed more often (Figure 3) – though the relation between clinical complexity and TOPFA was less clear.

Finally, neonatal mortality also varied regionally. The study did not assess in depth the system or personal factors associated with such variation. However, we noted that the neonatal mortality was highest in Malta and Argentina where termination of pregnancy is not allowed and prenatal detection of CCHD is low. The lowest neonatal mortality was found in countries where the TOPFA proportions were highest. These findings, though not conclusive, suggest two possibilities. First, prenatal detection might help improve the care of babies with CCHD, by allowing for a better plan of care at birth when compared to the unanticipated urgency at birth if no prenatal diagnosis was made

For peer review only - http://bmjopen.bmj.com/site/about/guidelines.xhtml
For peer review only

[12,13]. Second, terminations of pregnancy may disproportionately include the anatomically more severe cases (even within the same CCHD type), such that the overall survival is skewed towards what might be only an apparent improvement in outcomes [35].

Strengths and weaknesses of the study

The study has several strengths, including the large sample of the CCHD cohort (>18,000 cases), and the systematic nature of case ascertainment whether through population-based or hospital-based programs. Including programs from areas with different policies and health care systems allowed us a wider view of the interrelated factors that can influence reported prevalence, ascertainment, and prenatal diagnosis. Programs submitted individual case records that were centrally reviewed by clinicians with expertise in genetics (LDB, JEHB) and pediatric cardiology (LDB). This review aimed at harmonizing the CCHD diagnoses (e.g., cases with more than one CCHD code were systematically assigned a primary diagnosis) as well as the clinical classification as isolated, MCA, or syndromic case. The study also has limitations. The quality and completeness of the data submitted centrally depends on the program's methods related to data collection, coding, and classification (e.g., the degree to which clinical staff is involved in these processes). Also, we did not have details on the severity of each CCHD case, which may have contributed to variation across programs. For example, the clinical presentation of lesions such as AoS and COA can range from mild (e.g., not readily identifiable prenatally, or clinically at birth) to severe (e.g., a truly critical condition in the neonatal period). These variations would influence a program's ability to detect these conditions early in life or prenatally and would therefore affect findings such as the total prevalence and the proportion of cases prenatally diagnosed. A last limitation is the challenge and variability in ascertainment of pregnancies that ended in a termination.

Meaning of the study: possible mechanisms and implications for clinicians or policymakers

Ultimately, these findings, together with prior reports from the literature, have both public health and clinical implications for the care and prevention of CCHD. First, the high prevalence (1 in 500 births) underscores the universal need to address prevention and care of CCHD aggressively. Care in particular could be enhanced with earlier diagnosis. In this regard, prenatal diagnosis can complement pulse oximetry newborn screening, and compared to the latter, allow for more time and hence more thoughtful management decisions by well-informed families and clinicians [36,37].

The increasing trends in prenatal diagnosis rates also highlight the potential for significant changes in the epidemiology and clinical outcomes of CCHD. Although the magnitude of these trends vary in the included programs, the potential implications are vast. Prenatal diagnosis may continue to influence

For peer review only - http://bmjopen.bmj.com/site/about/guidelines.xhtml
For peer review only

the reported prevalence at birth as well as the outcomes (e.g., morbidity, survival) by a combination of more complete and timely detection and, to a varying extent, its influence on rates of terminations of pregnancy for fetal anomaly. The results of this study demonstrate the value of ongoing surveillance of CCHD in this changing environment.

Tracking and evaluating the patterns of CCHD occurrence is also important in the quest to discover the causes of these severe conditions. For example, in etiological studies, it is particularly important to include all affected fetuses, as stillbirths and terminations of pregnancy are more likely to be overrepresented in more severe cases. Failing to include such cases would limit the range and possibly skew the findings.

Finally, ongoing monitoring of the CCHD cohort, from pregnancy onwards, is important for researchers to appropriately evaluate long-term outcomes and track the burden of disease on population health.

Important questions remain. Is prenatal diagnosis improving population health? In an era of improving (and often more costly) diagnostic technology, are current systems increasing rather than eliminating potential health disparities? Are we providing the most current information about occurrence and outcomes to clinicians and families for appropriate counseling in the presence of a prenatally detected CCHD? Answering such questions requires a joint effort of epidemiologists and clinicians generating high quality information and tracking such data over time. Leveraging existing programs, data sharing and central clinical review and analysis may enhance efficiencies and inform these questions. International networks such as the ICBDSR, the National Birth Defects Prevention Network, and EUROCAT European surveillance of congenital anomalies can help provide the data, the analytic capacity, and a long term vision for a sustained and timely monitoring of the diagnostics, occurrence, and health impact of CCHD, as a tool for better prevention and care.

For peer review only

For peer review only - http://bmjopen.bmj.com/site/about/guidelines.xhtml

Footnotes

Contributors LDB conceived the study. MKB and LDB developed the protocol and supervised the study. MKB, LDB, and SK conducted the data analysis. LDB and JEHB reviewed the clinical classification of cases. MKB and LDB wrote the first draft of the article. EA, GC, JC, HEKdW, MG, BG, SL, WNN, AP, AR, SC, AS, ES, GT, DT curated and submitted the case data from their programs. All coauthors made substantial contributions to the conduct of the study, interpretation of results and critical revisions of the manuscript. Program directors are guarantors of the integrity of the data submitted for central analysis. MKB and LDB are overall guarantors.

Funding This research received no specific grant from any funding agency in the public, commercial or not-for-profit sectors. The programs participating in this study may receive local funding. The program in Czech Republic is supported by Ministry of Health of the Czech Republic, grant nr. AZV 17-29622A.

Competing interests none declared. All authors have completed the ICMJE uniform disclosure form at www.icmje.org/coi_disclosure.pdf

Patient consent not required. This was a pooled analysis of de-identified data from public health surveillance programs.

Provenance and peer review not commissioned; externally peer reviewed.

Data sharing statement No additional data are available.

Acknowledgements We thank each ICBDSR member program's staff for providing and processing the information on the cases with critical congenital heart defects and on the characteristics of their program.

Disclaimer The findings and conclusions in this report are those of the authors and do not necessarily represent the official position of the Centers for Disease Control and Prevention.

For peer review only

For peer review only - http://bmjopen.bmj.com/site/about/guidelines.xhtml

References

1 Dolk H, Loane M, Garne E, et al. Congenital heart defects in Europe: prevalence and perinatal mortality, 2000 to 2005, *Circulation* 2011;123:841-9.

2 Reller MD, Strickland MJ, Riehle-Colarusso T, et al. Prevalence of congenital heart defects in metropolitan Atlanta, 1998-2005, *J Pediatr* 2008;153:807-13

3 Oster ME, Lee KA, Honein MA, et al. Temporal trends in survival among infants with critical congenital heart defects, *Pediatrics* 2013;131:1502

4 Mackie AS, Tran DT, Marelli AJ, et al. Cost of congenital heart disease hospitalizations in Canada: A population-based study, *Can J Cardiol* 2017;33:792-8

5 Arth AC, Tinker SC, Simeone RM, et al. Inpatient hospitalization costs associated with birth defects among persons of all ages - United States, 2013, *MMWR Morb Mortal Wkly Rep* 2017;66:41-6

6 Boneva RS, Botto LD, Moore CA, et al. Mortality associated with congenital heart defects in the United States: trends and racial disparities, 1979-1997, *Circulation* 2001;103:2376-81

7 Gilboa SM, Salemi JL, Nembhard WN, et al. Mortality resulting from congenital heart disease among children and adults in the United States, 1999 to 2006, *Circulation* 2010;122:2254-63

8 Jortveit J, Eskedal L, Hirth A, et al. Sudden unexpected death in children with congenital heart defects, *Eur Heart J* 2016;37:621-6

9 Knowles RL, Bull C, Wren C, et al. Mortality with congenital heart defects in England and Wales, 1959-2009: exploring technological change through period and birth cohort analysis, *Arch Dis Child* 2012;97:861-5

10 Blyth M, Howe D, Gnanapragasam J, et al. The hidden mortality of transposition of the great arteries and survival advantage provided by prenatal diagnosis, *BJOG* 2008;115:1096-100

11 Bonnet D, Coltri A, Butera G, et al. Detection of transposition of the great arteries in fetuses reduces neonatal morbidity and mortality, *Circulation* 1999;99:916-8

12 Holland BJ, Myers JA, Woods CR. Prenatal diagnosis of critical congenital heart disease reduces risk of death from cardiovascular compromise prior to planned neonatal cardiac surgery: a meta-analysis, *Ultrasound Obstet Gynecol* 2015;45:631-8

For peer review only - http://bmjopen.bmj.com/site/about/guidelines.xhtml

13 Morris SA, Ethen MK, Penny DJ, et al. Prenatal diagnosis, birth location, surgical center, and neonatal mortality in infants with hypoplastic left heart syndrome, *Circulation* 2014;129:285-92.

14 Tworetzky W, McElhinney DB, Reddy VM, et al. Improved surgical outcome after fetal diagnosis of hypoplastic left heart syndrome, *Circulation* 2001;103:1269-73

15 Pinto NM, Keenan HT, Minich LL, et al. Barriers to prenatal detection of congenital heart disease: a population-based study, *Ultrasound Obstet Gynecol* 2012;40:418-25.

16 Clur SA, Bilardo CM. Early detection of fetal cardiac abnormalities: how effective is it and how should we manage these patients? *Prenat Diagn* 2014;34:1235-45

17 Egbe A, Uppu S, Lee S, et al. Changing prevalence of severe congenital heart disease: a population-based study, *Pediatr Cardiol* 2014;35:1232-8

18 Gilboa SM, Correa A, Botto LD, et al. Association between prepregnancy body mass index and congenital heart defects, *Am J Obstet Gynecol* 2010;202:51.e1-e10.

19 Baardman ME, Kerstjens-Frederikse WS, Corpeleijn E, et al. Combined adverse effects of maternal smoking and high body mass index on heart development in offspring: evidence for interaction? *Heart* 2012;98:474-9

20 Hoang TT, Marengo LK, Mitchell LE, et al. Original findings and updated meta-analysis for the association between maternal diabetes and risk for congenital heart disease phenotypes, *Am J Epidemiol* 2017;186:118-28

21 Jenkins KJ, Correa A, Feinstein JA, et al. Noninherited risk factors and congenital cardiovascular defects: current knowledge: a scientific statement from the American Heart Association Council on Cardiovascular Disease in the Young: endorsed by the American Academy of Pediatrics, *Circulation* 2007;115:2995-3014

22 Mills JL, Troendle J, Conley MR, et al. Maternal obesity and congenital heart defects: a population-based study, *Am J Clin Nutr* 2010;91:1543-9

23 Quartermain MD, Pasquali SK, Hill KD, et al. Variation in prenatal diagnosis of congenital heart disease in infants, *Pediatrics* 2015;136:e378-85

24 Wong SF, Chan FY, Cincotta RB, et al. Factors influencing the prenatal detection of structural congenital heart diseases, *Ultrasound Obstet Gynecol* 2003;21:19-25

For peer review only

For peer review only - http://bmjopen.bmj.com/site/about/guidelines.xhtml

25 Hill GD, Block JR, Tanem JB, et al. Disparities in the prenatal detection of critical congenital heart disease, *Prenat Diagn* 2015;35:859-63

26 Baardman ME, du Marchie Sarvaas, G J, de Walle HE, et al. Impact of introduction of 20-week ultrasound scan on prevalence and fetal and neonatal outcomes in cases of selected severe congenital heart defects in The Netherlands, *Ultrasound Obstet Gynecol* 2014;44:58-63

27 van Velzen CL, Clur SA, Rijlaarsdam ME, et al. Prenatal detection of congenital heart disease--results of a national screening programme, *BJOG* 2016;123:400-7

28 Chew C, Stone S, Donath SM, et al. Impact of antenatal screening on the presentation of infants with congenital heart disease to a cardiology unit, *J Paediatr Child Health* 2006;42:704-8

29 Corcoran S, Briggs K, O'Connor H, et al. Prenatal detection of major congenital heart disease - optimising resources to improve outcomes, *Eur J Obstet Gynecol Reprod Biol* 2016;203:260-3

30 van Velzen CL, Ket JCF, van de Ven, P M, et al. Systematic review and meta-analysis of the performance of second-trimester screening for prenatal detection of congenital heart defects, *Int J Gynaecol Obstet* 2018;140:137-45

31 Komisar J, Srivastava S, Geiger M, et al. Impact of changing indications and increased utilization of fetal echocardiography on prenatal detection of congenital heart disease, *Congenit Heart Dis* 2017;12:67-73

32 Ogge G, Gaglioti P, Maccanti S, et al. Prenatal screening for congenital heart disease with four-chamber and outflow-tract views: a multicenter study, *Ultrasound Obstet Gynecol* 2006;28:779-84

33 Sklansky MS, Berman DP, Pruetz JD, et al. Prenatal screening for major congenital heart disease: superiority of outflow tracts over the 4-chamber view*, J Ultrasound Med* 2009;28:889-99

34 Anonymous. Cardiac screening examination of the fetus: guidelines for performing the 'basic' and 'extended basic' cardiac scan, *Ultrasound Obstet Gynecol* 2006;27:107-13

35 Boyle B, Addor MC, Arriola L, et al. Estimating global burden of disease due to congenital anomaly: an analysis of European data, *Arch Dis Child Fetal Neonatal Ed* 2018;103:F22-F28

36 Bratt EL, Jarvholm S, Ekman-Joelsson BM, et al. Parent's experiences of counseling and their need for support following a prenatal diagnosis of congenital heart disease--a qualitative study in a Swedish context, *BMC Pregnancy Childbirth* 2015;15:171

For peer review only - http://bmjopen.bmj.com/site/about/guidelines.xhtml

For peer review only

37 Thakur V, Dutil N, Schwartz SM, et al. Impact of prenatal diagnosis on the management and early outcome of critical duct-dependent cardiac lesions, *Cardiol Young* 2018;28:548-53

For peer review only

For peer review only - http://bmjopen.bmj.com/site/about/guidelines.xhtml

Figures

Figure 1. Total prevalence and live birth prevalence (per 10,000 births) with 95% confidence intervals for 12 CCHD types, by program, International Clearinghouse for Birth Defects Surveillance and Research (ICBDSR) Critical Congenital Heart Defects (CCHD) Prenatal Diagnosis study 2000-2014.

[1] ICBDSR Programs, ordered by descending total prevalence, contributed data for different years within this time period, see table 1.
[2] India Chennai program is not included in prevalence estimates because for this exclusively prenatal program the denominator data (total births, total live births) are unavailable.

Figure 2. Proportion prenatally diagnosed and proportion of live births among all CCHD cases by program, International Clearinghouse for Birth Defects Surveillance and Research (ICBDSR) Critical Congenital Heart Defects (CCHD) Prenatal Diagnosis study 2000-2014.

[1] ICBDSR Programs, (ordered by descending prenatal diagnosis proportion), contributed data for different years within this time period, see table 1.
[2] India Chennai is not included in the figure because as an exclusively prenatal diagnosis program, all cases by design were prenatally diagnosed, and information on outcome of pregnancy is missing in the majority of cases.

Figure 3. Proportion of prenatally diagnosed CCHD cases according to clinical presentation and by program International Clearinghouse for Birth Defects Surveillance and Research (ICBDSR) Critical Congenital Heart Defects (CCHD) Prenatal Diagnosis study 2000-2014.

[1] Programs (ordered by descending prenatal detection proportion) contributed data for different years within this time period, see table 1. India Chennai is a prenatal diagnosis only program.
Abbreviations: MCA multiple congenital anomalies

Figure 4. First month mortality in live birth cases with selected CCHD by program, International Clearinghouse for Birth Defects Surveillance and Research (ICBDSR) Critical Congenital Heart Defects (CCHD) Prenatal Diagnosis study 2000-2014.

[1] ICBDSR programs (ordered by descending first month mortality) contributed data for different years within this time period, see table 1.
[2]India-Chennai and Canada are not included in the graph: Pregnancy outcomes in India-Chennai are poorly reported and Canada reported on mortality one year after birth, not specified in first week or first month mortality.

For peer review only

For peer review only - http://bmjopen.bmj.com/site/about/guidelines.xhtml

Supplementary Tables and Appendix

Table S1. Total prevalence per 10,000 births per year per program for selected CCHD, International Clearinghouse for Birth Defects Surveillance and Research (ICBDSR) Critical Congenital Heart Defects (CCHD) Prenatal Diagnosis study 2000-2014.

Table S2. Distribution of CCHD types per program (%). The proportions add to 100% per program, International Clearinghouse for Birth Defects Surveillance and Research (ICBDSR) Critical Congenital Heart Defects (CCHD) Prenatal Diagnosis study 2000-2014.

Appendix. Assigning a main diagnosis of critical congenital heart defects (CCHD).

For peer review only

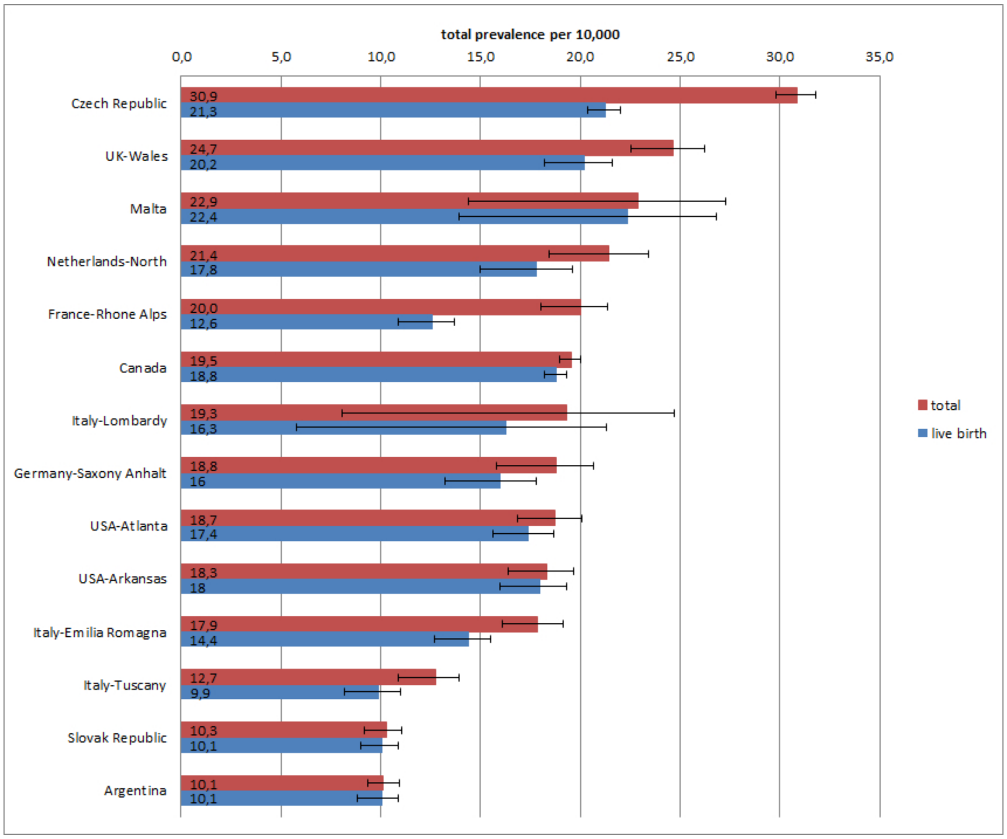

Figure 1. Total prevalence and live birth prevalence (per 10,000 births) with 95% confidence intervals for 12 CCHD types, by program, International Clearinghouse for Birth Defects Surveillance and Research (ICBDSR) Critical Congenital Heart Defects (CCHD) Prenatal Diagnosis study 2000-2014.
1 ICBDSR Programs, ordered by descending total prevalence, contributed data for different years within this time period, see table 1.
2 India Chennai program is not included in prevalence estimates because for this exclusively prenatal program the denominator data (total births, total live births) are unavailable.

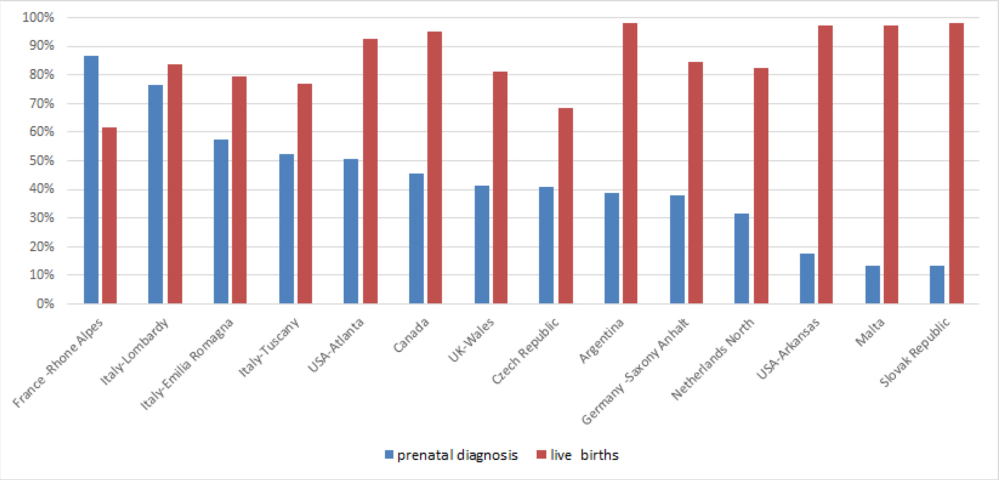

Figure 2. Proportion prenatally diagnosed and proportion of live births among all CCHD cases by program, International Clearinghouse for Birth Defects Surveillance and Research (ICBDSR) Critical Congenital Heart Defects (CCHD) Prenatal Diagnosis study 2000-2014.

1 ICBDSR Programs, (ordered by descending prenatal diagnosis proportion), contributed data for different years within this time period, see table 1.
2 India Chennai is not included in the figure because as an exclusively prenatal diagnosis program, all cases by design were prenatally diagnosed, and information on outcome of pregnancy is missing in the majority of cases.

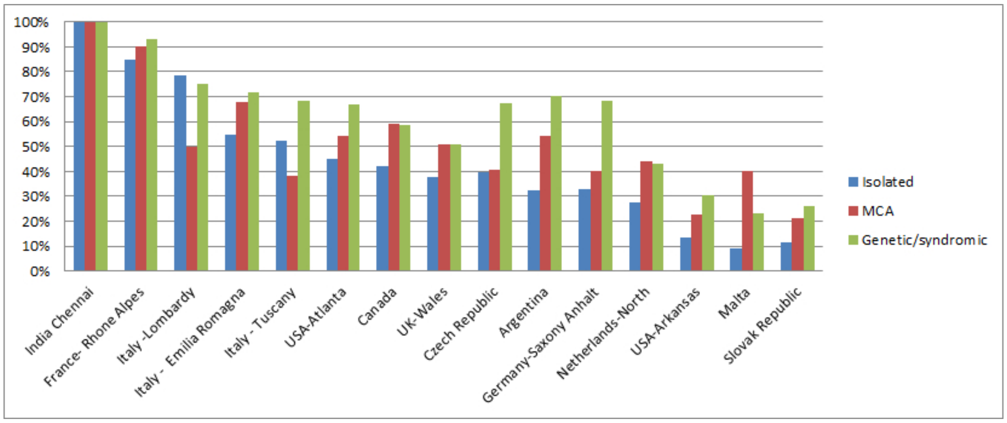

Figure 3. Proportion of prenatally diagnosed CCHD cases according to clinical presentation and by program International Clearinghouse for Birth Defects Surveillance and Research (ICBDSR) Critical Congenital Heart Defects (CCHD) Prenatal Diagnosis study 2000-2014.
1 Programs (ordered by descending prenatal detection proportion) contributed data for different years within this time period, see table 1. India Chennai is a prenatal diagnosis only program.
Abbreviations: MCA multiple congenital anomalies

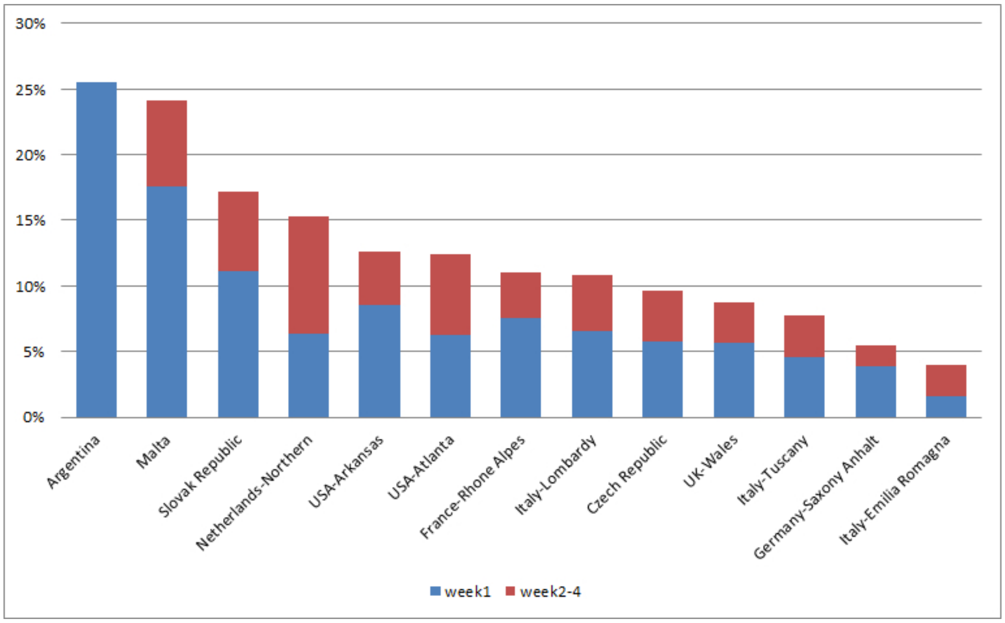

Figure 4. First month mortality in live birth cases with selected CCHD by program, International Clearinghouse for Birth Defects Surveillance and Research (ICBDSR) Critical Congenital Heart Defects (CCHD) Prenatal Diagnosis study 2000-2014.
1 ICBDSR programs (ordered by descending first month mortality) contributed data for different years within this time period, see table 1.
2India-Chennai and Canada are not included in the graph: Pregnancy outcomes in India-Chennai are poorly reported and Canada reported on mortality one year after birth, not specified in first week or first month mortality.

Supplementary Table S1. Total prevalence per 10,000 births per year per program for selected CCHD, , International Clearinghouse for Birth Defects Surveillance and Research (ICBDSR) Critical Congenital Heart Defects (CCHD) Prenatal Diagnosis study 2000-2014.[1,2]

Program by geographic region	2000	2001	2002	2003	2004	2005	2006	2007	2008	2009	2010	2011	2012	2013	2014	**Totals**	Trend
Czech Republic	33.0	34.9	32.5	33.7	37.4	29.7	25.6	37.9	33.1	28.8	33.4	30.3	27.3	16.0		**30.9**	\
UK-Wales		23.6	23.9	30.2	21.4	24.9	23.0	30.2	25.8	24.6	22.3	23.1	23.3			**24.7**	-
Malta		30.3	15.3	7.4	28.2	25.9	20.6	35.9	23.7	26.3	9.9	25.5	25.8			**22.9**	-
Netherlands-Northern		19.0	19.6	22.4	30.8	14.6	20.9	20.9	18.3	20.9	27.2	16.8	26.4			**21.4**	~
France-Rhone Alpes							18.9	16.5	16.9	19.6	17.7	23.0	26.6			**20.0**	/
Canada					19.3	20.9	19.2	19.6	19.4	18.8	18.6	20.6	20.4	19.2	19.2	**20.1**	-
Italy-Lombardy										22.1	16.4					**19.3**	nc
Germany-Saxony Anhalt		13.7	19.2	17.0	16.0	25.4	18.2	14.3	28.0	15.1	17.2	19.5	21.8			**18.8**	~
USA-Atlanta		18.9	24.1	19.0	18.0	20.5	18.0	17.0	14.9							**18.7**	\
USA-Arkansas		15.8	20.7	17.1	15.2	13.4	19.6	19.5	18.5	21.5	21.8					**18.3**	/
Italy-Emilia Romagna		18.3	16.5	18.4	18.7	18.5	16.2	15.6	14.3	17.6	17.2	24.5	18.9			**17.9**	-
Italy-Tuscany		15.1	9.0	12.2	12.7	9.8	15.5	11.6	13.8	13.0	18.0	12.8	8.6			**12.7**	-
Slovak Republic		8.9	12.5	10.2	11.9	10.2	10.0	7.3	7.8	10.2	12.7	9.5	12.0			**10.3**	-
Argentina														9.8	10.4	**10.1**	nc

[1]ICBDSR Programs contributed data for different years within this time period, see table 1.

[2]India Chennai program is not included in prevalence estimates because for this exclusively prenatal program the denominator data (total births, total livebirths) are unavailable.

/ significant increasing trend, \ significant decreasing trend, ~heterogeneous prevalence, - no trend, nc not calculated because of too few years

For peer review only

For peer review only - http://bmjopen.bmj.com/site/about/guidelines.xhtml

Supplementary Table S2 Distribution of CCHD types per program (%). The proportions add to 100% per program, , International Clearinghouse for Birth Defects Surveillance and Research (ICBDSR) Critical Congenital Heart Defects (CCHD) Prenatal Diagnosis study 2000-2014.[1]

	LVOTO			Conotruncal					RVOTO			
Program by geographic region	HLH	COA	AoS	TOF	DTGA	DORV	PTA	IAA	PulmA	TriA/HRH	SV	TAPVR
UK-Wales	13.5	20.1	10.2	14.4	13.7	5.2	4.5	3.3	5.2	2.1	3.5	4.5
Germany-Saxony Anhalt	14.3	23.7	6.9	17.3	17.6	3.8	3.3	1.3	4.6	1.8	2.3	3.1
Netherlands-Northern	15.5	17.0	10.3	15.5	17.2	5.2	1.9	2.1	6.1	2.1	4.0	3.1
France-Rhone Alpes	22.9	10.9	3.8	17.7	19.9	5.1	4.1	0.4	4.0	4.5	5.5	1.2
Italy-Emilia Romagna	14.6	17.5	3.1	21.1	16.1	6.9	4.0	0.9	4.4	4.7	4.4	2.3
Italy-Lombardy	16.4	25.5	5.5	25.5	7.3	7.3	1.8	0.0	3.6	3.6	3.6	0.0
Italy-Tuscany	18.4	16.0	3.8	19.5	21.1	6.2	2.4	0.4	3.5	3.1	4.2	1.3
Malta	18.0	18.0	5.4	14.4	20.7	2.7	1.8	1.8	5.4	3.6	7.2	0.9
Czech Republic	10.6	18.5	15.8	12.4	11.8	11.0	4.4	2.5	6.0	1.9	2.8	2.5
Slovak Republic	22.1	11.2	7.6	17.9	10.0	7.0	9.0	1.6	4.5	2.5	4.4	2.2
Canada	9.6	25.2	7.5	20.0	15.1	6.2	2.5	0.5	3.9	2.3	1.9	5.2
USA-Arkansas	17.5	25.6	10.7	4.7	13.3	6.0	3.5	2.9	3.7	2.5	4.3	5.4
USA-Atlanta	11.9	21.4	5.7	25.8	10.9	2.8	4.5	1.8	3.1	3.4	4.9	3.9
Argentina	18.6	15.1	2.6	14.8	14.4	4.9	4.4	3.0	3.4	2.5	11.3	4.9
India-Chennai	15.4	1.5	4.0	25.4	10.2	11.5	4.2	0.0	0.8	6.0	19.5	1.5

[1]ICBDSR programs contributed data for different years within this time period, see table 1.
Abbreviations: LVOTO left ventricular outflow tract obstruction, RVOTO right ventricular outflow tract obstruction, SV single ventricle, TAPVR total anomalous pulmonary venous return, CCHD critical congenital heart defects, HLHS hypoplastic left heart syndrome, COA coarctation of the aorta, AoS aortic valve stenosis, TOF tetralogy of Fallot, DTGA d-transposition of great arteries, DORV double outlet right ventricle, PTA persistent truncus arteriosus, IAA interrupted aortic arch, PulmA pulmonary valve atresia with intact ventricular septum, TriA/HRH tricuspid valve atresia / hypoplastic right heart, SV single ventricle, TAPVR total anomalous pulmonary venous return

supplementary tables and appendix_ Prenatal Diagnosis and Prevalence of Critical Congenital Heart Defects: an International Retrospective Cohort Study_MK Bakker et al. 2018

For peer review only

Appendix. Assigning a main diagnosis of critical congenital heart defects (CCHD)

In this study, programs submitted cases with at least one of 12 diagnoses considered to be consistent with CCHD. These diagnoses (identified by their ICD9 or ICD10 codes) were hypoplastic left heart syndrome (HLHS), coarctation of the aorta (COA), aortic valve stenosis (AoS), tetralogy of Fallot (TOF), d-transposition of great arteries (DTGA), double outlet right ventricle (DORV), persistent truncus arteriosus (PTA), interrupted aortic arch (IAA), pulmonary valve atresia with intact ventricular septum (PulmA), tricuspid valve atresia / hypoplastic right heart (TriA/HRH), single ventricle (SV) and total anomalous pulmonary venous return (TAPVR).

Most cases had just one of these diagnoses ('#CCHD dx' in table below), as noted in the column '# cases'. For the few cases with more than one CCHD code, a single CCHD code was assigned, using the system below for consistency. The rationale for the algorithm was as follows:

a) assign where possible the more severe diagnosis within the same spectrum. For example, in the case of left sided obstructive anomalies, the hierarchy was HLHS > CoA > AoS
b) assign the more dominant condition when diagnoses were not in the same spectrum. For example, in the case of IAA and several other types of CCHD (see below), the diagnosis of IAA prevailed. In the case of HLHS, a CCHD that is both severe clinically as well as easily identifiable at prenatal ultrasound examination, this diagnosis took precedence over several other types of CCHD (see table below). In the case of SV, some CCHD combinations were especially complex, so that the SV group ended up including fairly straightforward conditions such as double inlet left ventricle as well as more complex conditions, in which the SV morphology was joined by several other CCHD lesions.

Two points are worth noting. First, for some combinations of CCHD codes, there could be disagreements among experts as to which main diagnosis to assign. The approach used here was developed by the study's clinical team with expertise in medical genetics and pediatric cardiology. Ideally, a more granular approach might be preferable, to avoid grouping somewhat heterogeneous lesions. However, too many small groups would make the analysis unmanageable and a reasonable balance between 'splitting' and 'lumping' had to be achieved. In this case, the decision was made to be systematic (assignment based on specific code combinations) and explicit (full assignment table provided), to improve the clarity and reproducibility of the study. Second, the cases with multiple CCHD codes, and particularly those with more complex combinations, were a small fraction of all cases, so any disagreement on the assignment of such cases would likely have a minimal effect of the overall findings of the study.

For peer review only - http://bmjopen.bmj.com/site/about/guidelines.xhtml

# CCHD dx	AoS	COA	IAA	DORV	SV	HLHS	PulmA	TriA	TAPVR	DTGA	TOF	PTA	# cases	Final Assignment
1	1	0	0	0	0	0	0	0	0	0	0	0	1792	01.AoS
1	0	1	0	0	0	0	0	0	0	0	0	0	3945	02.COA
2	1	1	0	0	0	0	0	0	0	0	0	0	67	02.COA
1	0	0	1	0	0	0	0	0	0	0	0	0	249	03.IAA
2	0	1	1	0	0	0	0	0	0	0	0	0	7	03.IAA
2	1	0	1	0	0	0	0	0	0	0	0	0	4	03.IAA
2	0	0	1	0	0	0	0	1	0	0	0	0	3	03.IAA
2	0	0	1	0	0	0	0	0	1	0	0	0	2	03.IAA
2	0	0	1	1	0	0	0	0	0	0	0	0	2	03.IAA
3	1	0	1	1	0	0	0	0	0	0	0	0	2	03.IAA
2	0	0	1	0	0	0	0	0	0	0	1	0	1	03.IAA
2	0	0	1	0	0	0	1	0	0	0	0	0	1	03.IAA
3	0	1	1	1	0	0	0	0	0	0	0	0	1	03.IAA
1	0	0	0	1	0	0	0	0	0	0	0	0	1361	04.DORV
2	0	1	0	1	0	0	0	0	0	0	0	0	24	04.DORV
2	0	0	0	1	0	0	0	0	0	0	1	0	9	04.DORV
2	1	0	0	1	0	0	0	0	0	0	0	0	2	04.DORV
1	0	0	0	0	1	0	0	0	0	0	0	0	632	05.SV
2	0	0	0	0	1	0	0	0	0	1	0	0	29	05.SV
2	0	1	0	0	1	0	0	0	0	0	0	0	11	05.SV
2	0	0	0	0	1	0	0	1	0	0	0	0	10	05.SV
2	0	0	0	0	1	0	1	0	0	0	0	0	7	05.SV
3	0	1	0	0	1	0	0	0	0	1	0	0	7	05.SV
2	0	0	0	1	1	0	0	0	0	0	0	0	6	05.SV
2	0	0	0	0	1	0	0	0	0	0	0	1	4	05.SV
2	0	0	0	0	1	0	0	0	1	0	0	0	4	05.SV
2	0	0	1	0	1	0	0	0	0	0	0	0	4	05.SV
2	1	0	0	0	1	0	0	0	0	0	0	0	4	05.SV
3	0	0	0	1	1	0	0	0	0	1	0	0	4	05.SV
3	0	0	0	0	1	0	0	0	1	1	0	0	2	05.SV

supplementary tables and appendix_ Prenatal Diagnosis and Prevalence of Critical Congenital Heart Defects: an International Retrospective Cohort Study_MK Bakker et al. 2018

2	0	0	0	0	1	0	0	0	0	0	1	0	1	05.SV
3	0	0	0	0	1	0	0	0	0	1	1	0	1	05.SV
3	0	0	0	0	1	0	0	1	0	1	0	0	1	05.SV
3	0	0	0	0	1	0	1	0	0	0	0	1	1	05.SV
3	0	0	0	0	1	0	1	0	0	1	0	0	1	05.SV
3	0	0	0	1	1	0	0	0	1	0	0	0	1	05.SV
3	0	0	0	1	1	0	1	0	0	0	0	0	1	05.SV
3	0	0	1	0	1	0	0	0	0	1	0	0	1	05.SV
3	0	1	0	0	1	0	0	1	0	0	0	0	1	05.SV
3	0	1	0	1	1	0	0	0	0	0	0	0	1	05.SV
3	1	0	0	0	1	0	0	0	0	1	0	0	1	05.SV
4	0	0	0	1	1	0	1	1	0	0	0	0	1	05.SV
4	1	1	0	0	1	0	0	0	0	1	0	0	1	05.SV
1	0	0	0	0	0	1	0	0	0	0	0	0	2386	06.HLH
2	0	1	0	0	0	1	0	0	0	0	0	0	44	06.HLH
2	1	0	0	0	0	1	0	0	0	0	0	0	26	06.HLH
2	0	0	0	1	0	1	0	0	0	0	0	0	18	06.HLH
2	0	0	0	0	0	1	0	0	1	0	0	0	8	06.HLH
2	0	0	0	0	0	1	0	0	0	1	0	0	7	06.HLH
2	0	0	1	0	0	1	0	0	0	0	0	0	6	06.HLH
2	0	0	0	0	1	1	0	0	0	0	0	0	4	06.HLH
3	0	1	0	1	0	1	0	0	0	0	0	0	4	06.HLH
4	0	1	0	1	1	1	0	0	0	0	0	0	3	06.HLH
2	0	0	0	0	0	1	0	0	0	0	0	1	2	06.HLH
2	0	0	0	0	0	1	1	0	0	0	0	0	2	06.HLH
3	0	0	0	1	0	1	0	0	1	0	0	0	2	06.HLH
3	1	1	0	0	0	1	0	0	0	0	0	0	2	06.HLH
2	0	0	0	0	0	1	0	1	0	0	0	0	1	06.HLH
3	0	0	0	1	0	1	0	0	0	1	0	0	1	06.HLH
3	0	0	1	1	0	1	0	0	0	0	0	0	1	06.HLH
3	0	1	0	0	0	1	0	0	1	0	0	0	1	06.HLH
3	0	1	1	0	0	1	0	0	0	0	0	0	1	06.HLH

supplementary tables and appendix_ Prenatal Diagnosis and Prevalence of Critical Congenital Heart Defects: an International Retrospective Cohort Study_MK Bakker et al. 2018

3	1	0	0	0	1	1	0	0	0	0	0	0	1	06.HLH
4	0	0	0	1	0	1	1	0	0	1	0	0	1	06.HLH
4	0	1	0	0	1	1	0	0	1	0	0	0	1	06.HLH
4	1	1	0	0	0	1	0	1	0	0	0	0	1	06.HLH
4	1	1	0	1	0	1	0	0	0	0	0	0	1	06.HLH
1	0	0	0	0	0	0	1	0	0	0	0	0	1021	07.PulmA
2	0	0	0	1	0	0	1	0	0	0	0	0	9	07.PulmA
2	0	0	0	0	0	0	1	0	1	0	0	0	4	07.PulmA
2	1	0	0	0	0	0	1	0	0	0	0	0	2	07.PulmA
3	0	0	0	1	0	0	1	0	1	0	0	0	1	07.PulmA
4	1	1	0	0	0	0	1	0	1	0	0	0	1	07.PulmA
1	0	0	0	0	0	0	0	1	0	0	0	0	494	08.TriA
2	0	0	0	0	0	0	1	1	0	0	0	0	11	08.TriA
2	1	0	0	0	0	0	0	1	0	0	0	0	4	08.TriA
2	0	0	0	1	0	0	0	1	0	0	0	0	2	08.TriA
2	0	1	0	0	0	0	0	1	0	0	0	0	2	08.TriA
3	0	0	0	0	0	0	1	1	1	0	0	0	1	08.TriA
1	0	0	0	0	0	0	0	0	1	0	0	0	754	09.TAPVR
2	0	0	0	1	0	0	0	0	1	0	0	0	11	09.TAPVR
2	0	1	0	0	0	0	0	0	1	0	0	0	2	09.TAPVR
2	1	0	0	0	0	0	0	0	1	0	0	0	1	09.TAPVR
1	0	0	0	0	0	0	0	0	0	1	0	0	2711	10.DTGA
2	0	1	0	0	0	0	0	0	0	1	0	0	29	10.DTGA
2	0	0	0	1	0	0	0	0	0	1	0	0	15	10.DTGA
2	0	0	0	0	0	0	0	1	0	1	0	0	12	10.DTGA
2	0	0	0	0	0	0	1	0	0	1	0	0	9	10.DTGA
3	0	1	0	1	0	0	0	0	0	1	0	0	8	10.DTGA
3	0	1	0	0	0	0	0	1	0	1	0	0	6	10.DTGA
3	0	0	0	1	0	0	1	0	0	1	0	0	4	10.DTGA
2	0	0	0	0	0	0	0	0	1	1	0	0	3	10.DTGA
3	0	0	0	0	0	0	1	0	1	1	0	0	2	10.DTGA
3	0	0	0	1	0	0	0	0	1	1	0	0	2	10.DTGA

For peer review only - http://bmjopen.bmj.com/site/about/guidelines.xhtml

2	1	0	0	0	0	0	0	0	0	1	0	0	1	10.DTGA
3	1	0	0	0	0	0	0	0	0	1	1	0	1	10.DTGA
4	0	1	0	1	0	0	0	1	0	1	0	0	1	10.DTGA
1	0	0	0	0	0	0	0	0	0	0	1	0	3415	11.TOF
2	0	0	0	0	0	0	1	0	0	0	1	0	4	11.TOF
2	0	0	0	0	0	0	0	0	1	0	1	0	3	11.TOF
2	0	1	0	0	0	0	0	0	0	0	1	0	2	11.TOF
2	1	0	0	0	0	0	0	0	0	0	1	0	2	11.TOF
3	0	0	0	1	0	0	0	0	1	0	1	0	2	11.TOF
2	0	0	0	0	0	0	0	1	0	0	1	0	1	11.TOF
1	0	0	0	0	0	0	0	0	0	0	0	1	713	12.PTA
2	0	0	1	0	0	0	0	0	0	0	0	1	13	12.PTA
2	1	0	0	0	0	0	0	0	0	0	0	1	3	12.PTA
2	0	0	0	0	0	0	1	0	0	0	0	1	2	12.PTA
2	0	0	0	0	0	0	0	0	0	1	0	1	1	12.PTA
2	0	0	0	0	0	0	0	0	1	0	0	1	1	12.PTA
3	0	0	0	1	0	0	0	0	1	0	0	1	1	12.PTA
3	0	0	1	0	0	0	0	0	0	0	1	1	1	12.PTA

For peer review only - http://bmjopen.bmj.com/site/about/guidelines.xhtml

STROBE 2007 (v4) checklist of items to be included in reports of observational studies in epidemiology*
Checklist for cohort, case-control, and cross-sectional studies (combined)

Section/Topic	Item #	Recommendation	Reported on page #
**Title and abstract**	1	(*a*) Indicate the study's design with a commonly used term in the title or the abstract	1
		(*b*) Provide in the abstract an informative and balanced summary of what was done and what was found	2
**Introduction**			
Background/rationale	2	Explain the scientific background and rationale for the investigation being reported	4
Objectives	3	State specific objectives, including any pre-specified hypotheses	4
**Methods**			
Study design	4	Present key elements of study design early in the paper	5
Setting	5	Describe the setting, locations, and relevant dates, including periods of recruitment, exposure, follow-up, and data collection	5
Participants	6	(*a*) *Cohort study*—Give the eligibility criteria, and the sources and methods of selection of participants. Describe methods of follow-up<br>*Case-control study*—Give the eligibility criteria, and the sources and methods of case ascertainment and control selection. Give the rationale for the choice of cases and controls<br>*Cross-sectional study*—Give the eligibility criteria, and the sources and methods of selection of participants	5
		(*b*) *Cohort study*—For matched studies, give matching criteria and number of exposed and unexposed<br>*Case-control study*—For matched studies, give matching criteria and the number of controls per case	na
Variables	7	Clearly define all outcomes, exposures, predictors, potential confounders, and effect modifiers. Give diagnostic criteria, if applicable	5
Data sources/ measurement	8*	For each variable of interest, give sources of data and details of methods of assessment (measurement). Describe comparability of assessment methods if there is more than one group	5
Bias	9	Describe any efforts to address potential sources of bias	5/6
Study size	10	Explain how the study size was arrived at	5
Quantitative variables	11	Explain how quantitative variables were handled in the analyses. If applicable, describe which groupings were chosen and why	5
Statistical methods	12	(*a*) Describe all statistical methods, including those used to control for confounding	6
		(*b*) Describe any methods used to examine subgroups and interactions	6
		(*c*) Explain how missing data were addressed	na
		(*d*) *Cohort study*—If applicable, explain how loss to follow-up was addressed<br>*Case-control study*—If applicable, explain how matching of cases and controls was addressed	na

For peer review only

		Cross-sectional study—If applicable, describe analytical methods taking account of sampling strategy	
		(*e*) Describe any sensitivity analyses	na
**Results**			
Participants	13*	(a) Report numbers of individuals at each stage of study—eg numbers potentially eligible, examined for eligibility, confirmed eligible, included in the study, completing follow-up, and analysed	7
		(b) Give reasons for non-participation at each stage	na
		(c) Consider use of a flow diagram	na
Descriptive data	14*	(a) Give characteristics of study participants (eg demographic, clinical, social) and information on exposures and potential confounders	7, tables
		(b) Indicate number of participants with missing data for each variable of interest	table 3
		(c) *Cohort study*—Summarise follow-up time (eg, average and total amount)	na
Outcome data	15*	*Cohort study*—Report numbers of outcome events or summary measures over time	na
		Case-control study—Report numbers in each exposure category, or summary measures of exposure	na
		Cross-sectional study—Report numbers of outcome events or summary measures	7/8
Main results	16	(*a*) Give unadjusted estimates and, if applicable, confounder-adjusted estimates and their precision (eg, 95% confidence interval). Make clear which confounders were adjusted for and why they were included	7, tables and figures
		(*b*) Report category boundaries when continuous variables were categorized	na
		(*c*) If relevant, consider translating estimates of relative risk into absolute risk for a meaningful time period	na
Other analyses	17	Report other analyses done—eg analyses of subgroups and interactions, and sensitivity analyses	na
**Discussion**			
Key results	18	Summarise key results with reference to study objectives	9/10
Limitations	19	Discuss limitations of the study, taking into account sources of potential bias or imprecision. Discuss both direction and magnitude of any potential bias	11
Interpretation	20	Give a cautious overall interpretation of results considering objectives, limitations, multiplicity of analyses, results from similar studies, and other relevant evidence	11
Generalisability	21	Discuss the generalisability (external validity) of the study results	11
**Other information**			
Funding	22	Give the source of funding and the role of the funders for the present study and, if applicable, for the original study on which the present article is based	13

*Give information separately for cases and controls in case-control studies and, if applicable, for exposed and unexposed groups in cohort and cross-sectional studies.
Note: An Explanation and Elaboration article discusses each checklist item and gives methodological background and published examples of transparent reporting. The STROBE checklist is best used in conjunction with this article (freely available on the Web sites of PLoS Medicine at http://www.plosmedicine.org/, Annals of Internal Medicine at http://www.annals.org/, and Epidemiology at http://www.epidem.com/). Information on the STROBE Initiative is available at www.strobe-statement.org.

For peer review only

BMJ Open

Prenatal Diagnosis and Prevalence of Critical Congenital Heart Defects: an International Retrospective Cohort Study

Journal:	*BMJ Open*
Manuscript ID	bmjopen-2018-028139.R1
Article Type:	Research
Date Submitted by the Author:	10-May-2019
Complete List of Authors:	Bakker, Marian; University of Groningen,, Department of Genetics, Faculty of Medical Sciences<br>Bergman, Jorieke; University of Groningen,, Department of Genetics, Faculty of Medical Sciences<br>Krikov, Sergey; University of Utah, Division Medical Genetics<br>Amar, Emmanuelle; Registre Des Malformations en Rhone Alpes, REMERA<br>Cocchi, Guido; Neonatology Unit, S.Orsola-Malpighi Hospital, Department of Medical and Surgical Sciences, University of Bologna<br>Cragan, Janet; Metropolitan Atlanta Congenital Defects Program, National Center on Birth Defects and Developmental Disabilities, Centers for Disease Control and Prevention<br>de Walle, Hermien; University of Groningen,, Department of Genetics, Faculty of Medical Sciences<br>Gatt, Miriam; Malta Congenital Anomalies Registry, Directorate for Health Information and Research<br>Groisman, Boris; National Network of Congenital Anomalies of Argentina (RENAC), National Center of Medical Genetics, National Ministry of Health<br>Liu, Shiliang; University of Ottawa/Public Health Agency of Canada,<br>Nembhard, WN; University of Arkansas for Medical Sciences,<br>Pierini, Anna; Institute of Clinical Physiology, National Research Council and Fondazione Toscana Gabriele Monasterio, Tuscany Registry of Congenital Defects<br>Rissmann, Anke; Malformation Monitoring Centre, Medical Faculty, Otto-von-Guericke University<br>Chidambarathanu, S; Birth Defects Registry of India, Mediscan Systems<br>Sipek Jr, Antonin; Institute of Medical Biology and Genetics First Faculty of Medicine Charles University and General University Hospital<br>Szabova, Elena; Slovak Teratologic Information Centre (FPH), Slovak Medical University<br>Tagliabue, Giovanna; Lombardy Birth Defects Registry, Fondazione IRCCS Instituto Nazionale Tumori<br>Tucker, David; Congenital Anomaly Register and Information Service for Wales, Public Health Wales<br>Mastroiacovo, Pierpaolo; International Centre on Birth Defects<br>Botto, Lorenzo; University of Utah, Pediatrics; International Centre on Birth Defects
**Primary Subject Heading**:	Obstetrics and gynaecology

Secondary Subject Heading:	Public health, Epidemiology, Paediatrics
Keywords:	critical congenital heart defects, Prenatal diagnosis < OBSTETRICS, EPIDEMIOLOGY

SCHOLARONE™
Manuscripts

Prenatal Diagnosis and Prevalence of Critical Congenital Heart Defects: an International Retrospective Cohort Study

MK Bakker[1], JEH Bergman[1], S. Krikov[2], E Amar[3], G Cocchi[4], J Cragan[5], HEK de Walle[1], M Gatt[6], B Groisman[7], S Liu[8], WN Nembhard[9], A Pierini[10], A Rissmann[11], S Chidambarathanu[12], A Sipek Jr[13], E Szabova[14], G Tagliabue[15], D Tucker[16], P Mastroiacovo[17], LD Botto[2,17]

[1]University of Groningen, University Medical Center Groningen, Department of Genetics, Eurocat registration Northern Netherlands, Groningen, The Netherlands
[2] Division of Medical Genetics, Department of Pediatrics, University of Utah, Salt Lake City, USA
[3]Registre Des Malformations en Rhone Alpes, REMERA, Lyon, France
[4] Neonatology Unit, S.Orsola-Malpighi Hospital, Department of Medical and Surgical Sciences, University of Bologna, Bologna, Italy. IMER Registry, Italy
[5] Metropolitan Atlanta Congenital Defects Program, National Center on Birth Defects and Developmental Disabilities, Centers for Disease Control and Prevention, Atlanta, Georgia, USA
[6] Malta Congenital Anomalies Registry, Directorate for Health Information and Research, Malta
[7] National Network of Congenital Anomalies of Argentina (RENAC), National Center of Medical Genetics, National Ministry of Health, Argentina
[8]Maternal, Child and Youth Health Division, Public Health Agency of Canada, Ottawa, Canada
[9] University of Arkansas for Medical Sciences, Fay W Boozman College of Public Health and the Arkansas Children's Research Institute, Arkansas Reproductive Health Monitoring System, Little Rock, Arkansas, USA
[10] Institute of Clinical Physiology, National Research Council and Fondazione Toscana Gabriele Monasterio, Tuscany Registry of Congenital Defects, Pisa, Italy
[11] Malformation Monitoring Centre, Medical Faculty, Otto-von-Guericke University, Magdeburg, Germany
[12] Birth Defects Registry of India, Mediscan Systems, Chennai, India
[13] Institute of Medical Biology and Genetics First Faculty of Medicine Charles University and General University Hospital, Prague, Czech Republic
[14] Slovak Teratologic Information Centre (FPH), Slovak Medical University, Bratislava, Slovak Republic
[15] Lombardy Birth Defects Registry, Fondazione IRCCS Instituto Nazionale Tumori, Milan, Italy
[16] Congenital Anomaly Register and Information Service for Wales, Public Health Wales, Swansea, Wales
[17] International Centre on Birth Defects, University of Utah, Utah, USA

Corresponding author

Marian K Bakker, University Medical Center Groningen, Department of Genetics, Eurocat Northern Netherlands, PO Box 30001, 9700 RB Groningen, The Netherlands;
m.k.bakker@umcg.nl; +31 50-3617114

For peer review only - http://bmjopen.bmj.com/site/about/guidelines.xhtml

Abstract

Objectives: To assess international trends and patterns of prenatal diagnosis of critical congenital heart defects (CCHD) and their relation to total and live birth CCHD prevalence and mortality

Setting: Fifteen birth defect surveillance programs that participate in the International Clearinghouse for Birth Defect Surveillance and Research (ICBDSR), from 12 countries in Europe, North and South America and Asia.

Participants: Live births, stillbirths, and elective terminations of pregnancy for fetal anomaly diagnosed with one of 12 selected CCHD, ascertained by the 15 programs for delivery years 2000 to 2014.

Results: 18,243 CCHD cases were reported among 8,847,081 births. The median total prevalence was 19.1 per 10,000 births but varied three-fold between programs from 10.1 to 31.0 per 10,000. CCHD were prenatally detected for at least 50% of the cases in one-third of the programs. However, prenatal detection varied from 13% in Slovak Republic to 87% in some areas in France. Prenatal detection was consistently high for hypoplastic left heart syndrome (64% overall), and was lowest for total anomalous pulmonary venous return (28% overall). Surveillance programs in countries that do not legally permit terminations of pregnancy tended to have higher live birth prevalence of CCHD. Most programs showed an increasing trend in prenatally diagnosed CCHD cases.

Discussion and Conclusions: Prenatal detection already accounts for 50% or more of CCHD detected in many programs and is increasing. Local policies and access likely account for the wide variability of reported occurrence and prenatal diagnosis. Detection rates are high especially for CCHD that are more easily diagnosed on a standard obstetric 4-chamber ultrasound, or for fetuses that have extracardiac anomalies. These ongoing trends in prenatal diagnosis, potentially in combination with newborn pulse oximetry, are likely to modify the epidemiology and clinical outcomes of CCHD in the near future.

Trial registration: -

For peer review only

For peer review only - http://bmjopen.bmj.com/site/about/guidelines.xhtml

Strengths and limitations of this study

- This retrospective cohort study includes a large sample of more than 18,000 cases with critical congenital heart defects from 15 birth defect surveillance programs from Europe, North and South America and Asia.
- The programs come from areas with different policies regarding prenatal screening and diagnosis and therefore allow a wider view of factors related to prevalence, ascertainment, and prenatal diagnosis.
- The individual case records were centrally reviewed by clinicians with expertise in genetics and pediatric cardiology in order to harmonize diagnoses and clinical classification.
- The quality and completeness of the data depends on the program's methods related to data collection, coding, and classification.
- Details on the severity of each case were not available.

For peer review only

For peer review only - http://bmjopen.bmj.com/site/about/guidelines.xhtml

Introduction

Congenital heart defects (CHD) are among the most common birth defects, affecting approximately 1 in 100 births [1,2]. About 20 to 25% of CHD, or about 1 in 500 births, have been described as critical congenital heart defects (CCHD) because they require urgent and significant medical and surgical care to ensure survival [1,3]. CCHD represent a significant clinical and public health challenge. In lower-income countries, where complex health resources are the scarcest, CCHD are associated with very high mortality. In high income countries, including North America and Europe, CCHD are associated with lifelong morbidities and, for healthcare systems, with some of the leading drivers for pediatric in-hospital care costs [4,5].

Treatment and outcomes of CCHD have improved dramatically over the last decades [6-9]. A major part of the treatment strategy is to identify CCHD as early as possible, so that a management plan can be agreed upon and put in place prior to the baby presenting acutely and often in cardiac failure [10-14]. Prenatal diagnosis and newborn screening are two such early detection strategies, with prenatal diagnosis allowing for more deliberate management planning with family and care providers.

Prenatal detection of CCHD depends on several factors, including technology (the availability of adequate equipment), sonographer skills (CCHD detection requires more experience than the standard prenatal anatomic scan), screening policies and access to prenatal screening services (location and costs) [15,16]. Because these factors vary by country, within a country, and over time, as services and policies evolve, so will the rate and impact of prenatal diagnosis of CCHD. In turn, the rate of prenatal diagnosis can have multiple consequences on the pattern, trends, and outcomes of CCHD in a given population. Through earlier detection, prenatal diagnosis will improve overall ascertainment of CCHD by the time of birth, which could be reflected in more accurate estimates of prevalence at birth, by birth registries. This in turn can improve longitudinal population-based surveillance of CCHD-related outcomes through registry or linkage studies. Prenatal detection may also be associated with elective terminations of pregnancy for fetal anomaly (TOPFA), possibly reducing the live birth prevalence of CCHD and changing the overall pattern of CHD in the population [17]. Thus, prenatal diagnosis of CCHD has the potential of changing the epidemiology and public health impact of CCHD in complex ways. In this study we examined the changing trends of prenatal diagnosis of CCHD and their impact on CCHD birth prevalence and mortality in a geographically diverse set of programs that participate in the International Clearinghouse for Birth Defect Surveillance and Research (ICBDSR).

For peer review only - http://bmjopen.bmj.com/site/about/guidelines.xhtml
For peer review only

Methods

Study Design and Contributing Programs. This retrospective cohort study is based on data from 15 birth defect surveillance programs (Table 1) that are members of the ICBDSR. The ICBDSR is an international network of birth defects surveillance and research programs, whose mission is collaborative surveillance of birth defects and research into their causes and outcomes [www.icbdsr.org]. The 15 programs represent 12 countries from Europe, North America, South America and Asia**.** Participating programs had to be able to provide case-level data with specific diagnoses for CHD and extracardiac malformations for at least two birth years. Most contributing programs are population-based; the remainder are hospital-based. The program from India is hospital-based and a solely prenatal program, meaning that only cases that are prenatally diagnosed within the contributing hospitals are registered within the program. The other programs include both prenatally and postnatally diagnosed cases.

Data contributed. The study included cases (live births, stillbirths, and TOPFAs, depending on program) with one of 12 types of CCHD: hypoplastic left heart syndrome (HLHS), coarctation of the aorta (COA), aortic valve stenosis (AoS), tetralogy of Fallot (TOF), d-transposition of great arteries (DTGA), double outlet right ventricle (DORV), persistent truncus arteriosus (PTA), interrupted aortic arch (IAA), pulmonary valve atresia with intact ventricular septum (PulmA), tricuspid valve atresia / hypoplastic right heart (TriA/HRH), single ventricle (SV) and total anomalous pulmonary venous return (TAPVR). These CCHD are identifiable prenatally through ultrasound either by a 4-chamber view or an outflow tract view. The programs review medical records and abstract clinical information including the diagnoses, which, depending on local practices, are made by obstetricians or pediatric cardiologist dependings. The diagnoses are coded and classified by trained registry staff. For each case with one of the 12 selected CCHD, programs provided the following key information: type of CCHD (International Classification of Disease (ICD) 9th revision-Clinical Modification (CM) or ICD-10-CM code plus verbatim description (if available), timing of diagnosis (prenatal versus postnatal), pregnancy outcome (live birth (LB), stillbirths (SB), termination of pregnancy for fetal anomaly (TOPFA)), presence of extracardiac anomalies (structural malformations or syndrome diagnoses, as ICD code plus verbatim description), and, for live births, survival up to one year of age. Cases with an end-of-pregnancy date (delivery or termination of pregnancy) between 2000 and 2014 were included in the study. Most programs provided data for the time period from 2001 to 2012. Italy–Lombardy provided data on 2009 and 2010 and Argentina provided data on birth years 2013-2014. For the years for which they provided cases, programs also provided corresponding yearly denominator data, including total number of live births and total number of stillbirths.

For peer review only - http://bmjopen.bmj.com/site/about/guidelines.xhtml

For cases with more than one CCHD diagnosis, one clinical geneticist with specific expertise in pediatric cardiology (LDB) developed a structured hierarchical process to assign a single main CCHD diagnosis (for details see the Appendix in the supplementary material). In addition, two clinical geneticists (LDB and JEHB) reviewed all cases with extracardiac or syndrome diagnoses to classify the case either as isolated, with multiple congenital anomalies (MCA)or genetic/syndromic. MCA was defined as any combination of congenital anomalies (cardiac plus one or more extracardiac anomalies) without a recognized underlying cause (genetic or teratogenic) and not constituting a sequence.

Along with case data, programs also completed a short questionnaire on local practices and policies related to prenatal diagnosis and pregnancy termination. With the exception of Argentina and Malta, termination of pregnancy was legal in the areas covered by contributing programs (Table 1). In all regions covered by the programs, ultrasound scans are performed as part of standard obstetric care, including a scan around 18-20 weeks. These scans, depending on local health care systems, can be free of charge. . In the Netherlands a routine screening program for congenital anomalies is offered since 2007, in Argentina screening is part of standard obstetric care but depends on availability of technology.

Analyses. The analyses focused on prevalence, time of detection, clinical presentation, and survival. Because some programs contributed considerably more cases than others, and because a main goal of the study was to examine variations across programs and countries, the findings are presented primarily by program rather than in the aggregate. We calculated total prevalence and live birth prevalence, with 95% confidence interval (CI) computed based on the normal distribution. Total prevalence was calculated as total cases (LB+SB+TOPFA) divided by births (LB+SB), expressed per 10,000 births. Live birth prevalence was calculated as number of live born cases divided by total number of live births per 10,000 births. For programs that contributed more than two years of data, we examined time trends in total prevalence, and used the X^2 test for trend. Timing of detection of the CCHD (prenatal versus postnatal) was examined by program, by CCHD type and by clinical presentation (isolated, MCA, genetic/syndromic). The proportion prenatally diagnosed over time was also examined for trends (X^2 test for trend). Analyses were performed in Excel (Microsoft Office Professional plus 2010) and IBM SPSS Statistics for Windows, version 23.0 (Armonk, NY: IBM Corp). Each program has local approved procedures for ethics approval, and because this study was done using de-identified data no additional ethics committee approval was required.

Patient and Public involvement. No patients were involved in setting the research question or the outcome measures, nor were they involved in developing plans for implementation of the study. No

For peer review only - http://bmjopen.bmj.com/site/about/guidelines.xhtml

patients were asked to advise on interpretation or writing up of results. There are no plans to disseminate the results of the research to study participants or the relevant patient community.

For peer review only

For peer review only - http://bmjopen.bmj.com/site/about/guidelines.xhtml

Table 1. Selected geographic, registration procedure, and policy characteristics of participating surveillance programs, International Clearinghouse for Birth Defects Surveillance and Research (ICBDSR) Critical Congenital Heart Defects (CCHD) Prenatal Diagnosis study 2000-2014.

Country	Area	Type of program[1]	Ascertainment period	TOPFA legal	Stillbirth definition for study	Birth Years contributed to study
UK	Wales	P	18 years	Yes	≥ 24 WGA	2001-2012
Germany	Saxony Anhalt	P	1 year	Yes	≥ 500g	2001-2012
Netherlands	Northern	P	10 years	Yes	≥ 24 WGA	2001-2012
France	Rhone Alpes	P	18 years	Yes	≥ 20 WGA	2006-2012
Italy	Emilia Romagna	P	1 year	Yes	≥ 20 WGA	2001-2012
Italy	Lombardy	P	6 years	Yes	≥ 23 WGA	2009-2010
Italy	Tuscany	P	1 year	Yes	≥ 20 WGA	2001-2012
Malta	National	P	1 year	**No**	≥ 22 WGA or >500g	2001-2012
Czech Republic	National	P	15 years	Yes	≥ 28 WGA or ≥1000g	2000-2013
Slovak Republic	National	P	hospital discharge	Yes	≥1000g	2001-2012
Canada	National	P	1 year	Yes	≥ 20 WGA or >500g (or ≥22 WGA if birth weight is unknown)	2004-2014
USA	Arkansas	P	2 years	Yes	≥ 20 WGA or >350g	2001-2010
USA	Atlanta	P	6 years	Yes	≥ 20 WGA	2001-2008
Argentina	National	H	hospital discharge	**No**	≥500g	2013-2014
India	Chennai	H	prenatal only	Yes	n.a.	2008-2012

[1]Type of program: H –hospital-based, P-population based

[2]Data for Quebec not included (not available)

Abbreviations: CCHD-- critical congenital heart defects, TOPFA-- termination of pregnancy for fetal anomaly, WGA-- weeks of gestational age, n.a.-- not applicable (live fetuses only, prenatal screening program)

For peer review only - http://bmjopen.bmj.com/site/about/guidelines.xhtml

Results

Prevalence. Programs ascertained 18,243 CCHD cases among 8,847,081 births. The median prevalence was 19.1 per 10,000 births or 1 in 524 births (inter quartile range (IQR): 18.2-22.2 per 10,000 births). The highest total prevalence was observed in the Czech Republic (30.9 per 10,000 births) and the lowest in Slovak Republic and Argentina (10.3 and 10.1 per 10,000 births respectively, Table 2 and Figure 1). The highest live birth prevalence among all programs was observed in Malta (22.4 per 10,000). During the study period, CCHD showed an increasing trend in total prevalence in France-Rhone Alpes and USA-Arkansas, a decreasing trend in the Czech Republic and USA-Atlanta, and more complex trends in Northern Netherlands and Germany-Saxony Anhalt (Supplementary Table S1).

The difference between total and live birth prevalence of CCHD (Figure 1) reflected the proportion of TOPFA cases (Table 3). The proportion of TOPFA cases varied several-fold in programs in which TOPFA were legal, from <1% in USA-Arkansas to 24% in the Czech Republic and 35% in France-Rhone Alpes. In Malta and Argentina termination of pregnancy is not allowed. In India Chennai, information on the outcome of pregnancy was unavailable in the majority of cases. The proportion of stillbirth CCHD cases was small, on average 2% of total cases, with minor differences among programs (highest SB proportion of 4% in Northern Netherlands).

Patterns and distribution of the 12 CCHD types. The total prevalence by CCHD type is presented by program in Table 2. Although the prevalence varied, the proportion of CCHD types was similar among programs. Five CCHD types - HLHS, CoA and AoS (left ventricular outflow tract obstruction anomalies), TOF, and DTGA - accounted for 71% of cases, with some variations among programs (80% in Lombardy and 56% in India, Supplementary Table S2).

Prenatal diagnosis. There was considerable variation in proportion of CCHD identified via prenatal diagnosis among programs (Figure 2), from 87% in France-Rhone Alpes to 13% in Malta and Slovak Republic. In India-Chennai, an exclusively prenatal diagnosis program, all cases by design were prenatally diagnosed. In programs with a high proportion of prenatally diagnosed CCHD cases, the proportion of live births tended to be lower and the proportion of TOPFA higher. The converse was also true: the proportion of live birth cases was higher in programs with a low fraction of prenatally diagnosed cases.

In most programs, the proportion of CCHD cases prenatally diagnosed increased considerably during the study period, in some cases several-fold (Table 4). The proportion prenatally diagnosed also varied by type of CCHD. Such proportion was higher for HLHS and SV, which markedly affect

For peer review only - http://bmjopen.bmj.com/site/about/guidelines.xhtml

ventricular morphology, and lower for dTGA, TAPVR, and AoS, which affect ventricular morphology less markedly or frequently, thereby making prenatal detection more difficult. Among CCHD types, the fraction prenatally diagnosed varied considerably between programs but the rank order was similar (Table 5). For example, the proportion of HLHS cases prenatally diagnosed varied from 24% in Slovak Republic to 95% in France-Rhone Alpes and 100% in Italy-Lombardy, but within each program HLHS was the CCHD diagnosed prenatally most frequently.

Clinical presentation. The proportion of prenatally detected cases was higher in syndromic and MCA CCHD cases compared to isolated cases, and the difference was more pronounced in programs with lower overall prenatal detection proportion (Figure 3). Overall, most CCHD present as isolated (80%), with variations between programs. In Italy-Tuscany and Czech Republic 90% of the CCHD cases presented as isolated whereas in USA-Arkansas and USA-Atlanta 68% presented as isolated (Table 2). Some CCHD types were more commonly reported as isolated (AoS, DTGA, TRiA/HRH, HLHS and COA in >80% of the cases) compared to others such as PTA and IAA, which had a higher proportion of syndromic cases (> 17% of the cases, data not shown).

Mortality in first month of life. Because of the variations in follow-up period among programs, we focused the analysis on neonatal mortality (mortality by the first month of life in live births). The highest neonatal mortality was found in Argentina (25.5%) and Malta (24.1%) (Figure 4). In these countries, termination of pregnancy is not allowed and prenatal detection for CCHD is relatively low (Table 5 and Figure 2). The lowest neonatal mortality was found in Emilia Romagna (4.0%), Germany-Saxony Anhalt (5.4%), Tuscany (7.8%), UK–Wales (8.7%), Czech Republic (9.6%), Italy- Lombardy (10.9%) and France-Rhone Alpes (11.1%). In these programs, TOPFA proportions are comparatively high (Table 2).

For peer review only

For peer review only - http://bmjopen.bmj.com/site/about/guidelines.xhtml

Table 2 – Total prevalence of CCHD types per 10,000 births, , International Clearinghouse for Birth Defects Surveillance and Research (ICBDSR) Critical Congenital Heart Defects (CCHD) Prenatal Diagnosis study 2000-2014.[1]

Program (by geographic region)	HLHS	COA	AoS	TOF	DTGA	DORV	PTA	IAA	PulmA	TriA/ HRH	SV	TAPVR	Total prevalence
UK-Wales	3.3	5.0	2.5	3.5	3.4	1.3	1.1	0.8	1.3	0.5	0.9	1.1	24.7
Germany-Saxony Anhalt	2.7	4.5	1.3	3.3	3.3	0.7	0.6	0.2	0.9	0.3	0.4	0.6	18.8
Netherlands-Northern	3.3	3.6	2.2	3.3	3.7	1.1	0.4	0.4	1.3	0.4	0.9	0.7	21.4
France-Rhone Alpes	4.6	2.2	0.8	3.5	4.0	1.0	0.8	0.1	0.8	0.9	1.1	0.2	20.0
Italy-Lombardy	3.2	4.9	1.1	4.9	1.4	1.4	0.4	0.0	0.7	0.7	0.7	0.0	19.3
Italy-Emilia Romagna	2.6	3.1	0.6	3.8	2.9	1.2	0.7	0.2	0.8	0.8	0.8	0.4	17.9
Italy-Tuscany	2.3	2.0	0.5	2.5	2.7	0.8	0.3	0.1	0.5	0.4	0.5	0.2	12.7
Malta	4.1	4.1	1.2	3.3	4.7	0.6	0.4	0.4	1.2	0.8	1.6	0.2	22.9
Czech Republic	3.3	5.7	4.9	3.8	3.6	3.4	1.3	0.8	1.8	0.6	0.9	0.8	30.9
Slovak Republic	2.3	1.2	0.8	1.8	1.0	0.7	0.9	0.2	0.5	0.3	0.4	0.2	10.3
Canada	1.9	4.9	1.5	3.9	3.0	1.2	0.5	0.1	0.8	0.5	0.4	1.0	19.5
USA-Arkansas	3.2	4.7	2.0	0.9	2.4	1.1	0.6	0.5	0.7	0.5	0.8	1.0	18.3
USA-Atlanta	2.2	4.0	1.1	4.8	2.0	0.5	0.8	0.3	0.6	0.6	0.9	0.7	18.7
Argentina	1.9	1.5	0.3	1.5	1.5	0.5	0.4	0.3	0.3	0.2	1.1	0.5	10.1

[1] ICBDSR programs contributed data for different years within this time period, see table 1.

Abbreviations: CCHD-- critical congenital heart defects, HLHS-- hypoplastic left heart syndrome, COA-- coarctation of the aorta, AoS-- aortic valve stenosis, TOF-- tetralogy of Fallot, DTGA-- d-transposition of great arteries, DORV-- double outlet right ventricle, PTA-- persistent truncus arteriosus, IAA-- interrupted aortic arch, PulmA-- pulmonary valve atresia with intact ventricular septum, TriA/HRH-- tricuspid valve atresia / hypoplastic right heart, SV-- single ventricle TAPVR-- total anomalous pulmonary venous return

For peer review only

For peer review only - http://bmjopen.bmj.com/site/about/guidelines.xhtml

Table 3 Cases of CCHD by program and by pregnancy outcome and clinical presentation, International Clearinghouse for Birth Defects Surveillance and Research (ICBDSR) Critical Congenital Heart Defects (CCHD) Prenatal Diagnosis study 2000-2014.[1]

		Pregnancy outcome				Clinical presentation		
**Program - region**	**Total cases**	**LB**	**SB**	**TOPFA**	**Unknown**	**Isolated**	**MCA**	**syndromic**
UK-Wales	1 003	81.2%	2.5%	16.4%	0	71.6%	15.5%	13.0%
Germany-Saxony Anhalt	392	84.7%	2.0%	13.3%	0	74.7%	14.0%	11.2%
Netherlands-Northern	477	82.4%	4.2%	13.4%	0	74.8%	11.9%	13.2%
France-Rhone Alps	820	61.7%	3.2%	35.1%	0	70.0%	17.6%	12.4%
Italy-Emilia Romagna	795	79.5%	0.1%	20.4%	0	81.3%	9.4%	9.3%
Italy-Lombardy	55	83.6%	3.6%	12.7%	0	85.5%	7.3%	7.3%
Italy-Tuscany	451	77.2%	2.2%	20.6%	0	90.5%	4.7%	4.9%
Malta	111	97.3%	2.7%	na	0	79.3%	9.0%	11.7%
Czech Republic	4 569	68.4%	0.8%	23.6%	7.3%	89.6%	5.8%	4.6%
Slovak Republic	687	98.1%	0.4%	1.2%	0.3%	83.6%	10.9%	5.5%
Canada	6 157	95.2%	1.7%	3.1%	0	79.2%	11.6%	9.1%
USA-Arkansas	722	97.4%	2.1%	0.4%	0.1%	67.6%	20.2%	12.2%
USA-Atlanta	796	92.8%	2.9%	3.4%	0.9%	67.5%	13.7%	18.8%
Argentina	609	98.4%	1.5%	na	0.2%	75.5%	18.4%	6.1%
India-Chennai[2]	599	6.8%	0.7%	35.2%	57.3%	82.8%	15.4%	1.8%

[1] ICBDSR programs contributed data for different years within this time period, see Table 1.

[2] India-Chennai is a prenatal program, and only includes congenital heart defects that are prenatally diagnosed

Abbreviations: CCHD critical congenital heart defects, LB live births, SB stillbirths, TOPFA termination of pregnancy for fetal anomaly, MCA multiple congenital anomalies, na not available

For peer review only

For peer review only - http://bmjopen.bmj.com/site/about/guidelines.xhtml

Table 4 Proportion (%) of prenatally diagnosed CCHD cases by year and result of trend analyses, , International Clearinghouse for Birth Defects Surveillance and Research (ICBDSR) Critical Congenital Heart Defects (CCHD) Prenatal Diagnosis study 2000-2014.[1]

Program by geographic region	2000	2001	2002	2003	2004	2005	2006	2007	2008	2009	2010	2011	2012	2013	2014	Trend
Czech Republic	32.5	35.4	38.8	38.1	40.7	40.8	32.6	51.3	42.9	49.0	50.4	39.8	41.5	20.9		/
UK-Wales		21.9	30.1	32.6	32.9	42.7	38.5	37.1	55.9	40.2	55.6	55.4	50.6			/
Netherlands-Northern		5.3	10.0	17.8	13.6	18.5	31.6	32.4	33.3	44.7	58.3	51.7	65.9			/
France-Rhone Alpes							88.8	85.1	81.0	93.1	92.4	85.2	88.3			-
Canada					43.7	42.5	45.1	43.5	46.7	48.0	45.4	43.5	47.6	46.4	48.1	/
Germany-Saxony Anhalt		40.9	50.0	50.0	39.1	52.6	40.7	55.0	40.0	41.7	40.7	38.7	40.0			-
USA-Atlanta		42.2	41.2	38.1	38.7	48.1	76.7	75.0	66.7							/
USA-Arkansas		23.7	10.3	13.8	10.2	1.9	16.0	18.5	28.9	25.6	19.0					/
Italy-Emilia Romagna		51.1	60.9	64.7	69.6	64.3	67.7	40.6	60.7	53.9	43.8	55.0	61.3			-
Italy-Tuscany		40.0	20.8	35.3	48.6	46.4	50.0	52.8	59.5	55.0	62.5	74.4	73.1			/
Slovac republic		4.3	4.7	7.7	4.7	17.9	14.8	7.5	4.4	23.8	10.8	33.3	20.9			/
Malta				33.3	9.1		12.5	21.4	30.0	36.4		20.0				nc
Italy-Lombardy										75.0	78.3					nc
Argentina														33.5	47.0	nc
India-Chennai									100.0	100.0	100.0	100.0	100.0			nc

[1] ICBDSR programs contributed data for different years within this time period, see table 1.

/ significant increasing trend, - no trend, nc denotes not calculated because of too few data

Abbreviations: CCHD critical congenital heart defects

For peer review only - http://bmjopen.bmj.com/site/about/guidelines.xhtml

Table 5 – Proportion (%) of CCHD prenatally diagnosed, by CCHD type and program, , International Clearinghouse for Birth Defects Surveillance and Research (ICBDSR) Critical Congenital Heart Defects (CCHD) Prenatal Diagnosis study 2000-2014.[1,2]

	Selected CCHD												
**ICBDSR Program by geographic region**	**HLHS**	**SV**	**PulmA**	**TriA/ HRH**	**TOF**	**DTGA**	**DORV**	**PTA**	**IAA**	**COA**	**AoS**	**TAPVR**	**overall**
France- Rhone Alpes	95.2	100.0	100.0	94.6	84.1	90.2	95.2	70.6	100.0	66.3	61.3	50.0	**86.7**
Italy-Lombardy	100.0	50.0	100.0	100.0	85.7	50.0	75.0	100.0		57.1	66.7		**76.4**
Italy-Emilia Romagna	81.9	68.6	48.6	64.9	50.6	58.6	74.5	75.0	28.6	42.4	28.0	27.8	**57.6**
Italy-Tuscany	84.3	78.9	56.3	71.4	48.9	36.8	82.1	54.5	50.0	25.0	35.3	0.0	**52.3**
USA-Atlanta	77.9	76.9	60.0	70.4	50.7	43.7	63.6	61.1	50.0	34.7	33.3	16.1	**50.5**
Canada	57.8	38.3	52.5	42.0	48.6	34.1	56.0	52.3	50.0	42.4	42.4	46.4	**45.5**
Czech Republic	72.2	59.5	61.4	37.9	29.0	29.9	55.3	53.3	80.7	23.3	30.9	19.5	**40.9**
UK-Wales	88.1	77.1	48.1	66.7	36.8	37.2	57.7	71.1	27.3	17.8	11.8	17.8	**41.5**
Argentina	54.0	55.1	38.1	40.0	36.7	21.6	53.3	29.6	38.9	31.5	25.0	20.0	**38.6**
Germany-Saxony Anhalt	66.1	66.7	50.0	71.4	30.9	33.3	40.0	46.2	40.0	28.0	18.5	25.0	**38.0**
Netherlands-Northern	71.6	63.2	41.4	30.0	24.3	25.6	68.0	11.1	20.0	11.1	4.1	6.7	**31.7**
USA-Arkansas	42.1	32.3	14.8	38.9	14.7	10.4	25.6	36.0	14.3	5.4	2.6	5.1	**17.5**
Malta	25.0	25.0	16.7	25.0	12.5	13.0	33.3	0.0	0.0	0.0	0.0	0.0	**13.5**
Slovak Republic	24.3	10.0	3.2	11.8	6.5	5.8	18.8	21.0	9.1	13.0	5.8	0.0	**13.2**

Note: programs are ordered vertically by overall proportion of cases prenatally diagnosed (from high to low), whereas CCHD types are arranged horizontally left to right by approximate ease of prenatal diagnosis by fetal ultrasound (from more easily to less easily detectable on standard 4-chamber view).

[1] ICBDSR programs contributed data for different years within this time period, see table 1.

[2] India Chennai, an exclusively prenatal diagnosis program, is not included in the table because all cases by design were prenatally diagnosed.

Abbreviations: CCHD-- critical congenital heart defects, HLHS-- hypoplastic left heart syndrome, COA-- coarctation of the aorta, AoS-- aortic valve stenosis, TOF-- tetralogy of Fallot, DTGA-- d-transposition of great arteries, DORV-- double outlet right ventricle, PTA-- persistent truncus arteriosus, IAA-- interrupted aortic arch, PulmA-- pulmonary valve atresia with intact ventricular septum, TriA/HRH-- tricuspid valve atresia / hypoplastic right heart, SV-- single ventricle TAPVR-- total anomalous pulmonary venous return

For peer review only - http://bmjopen.bmj.com/site/about/guidelines.xhtml
For peer review only

Discussion

In this retrospective cohort study of more than 18,000 CCHD cases from 15 birth defect surveillance programs from Europe, North and South America and Asia, we observed several remarkable patterns and trends in the occurrence and prenatal diagnosis of CCHD.

First, CCHD are common regardless of geography and ascertainment program. The median total prevalence was 19 per 10,000 births, or approximately 1 in 500 births, similar to prior reports [1,3]. However, total prevalence varied three-fold among regions and programs (Figure 1). At least some and perhaps most of such variation is likely related to methodology, i.e., the local capacity to detect and report these conditions. Such methodologic factors include the ascertainment period after birth, ranging from days to years in the different programs (Table 1), and the ability to obtain a detailed diagnosis, both for the cardiac anomaly and extracardiac findings. For example, programs reporting the lowest prevalence rates (Slovak Republic and Argentina) have a short postnatal ascertainment period (at birth/ hospital discharge). Also, with few exceptions, programs with low prevalence rates tend to report few syndromic CCHD cases (Table 2). A further factor is a program's ability to ascertain and record terminations of affected pregnancies (Table 2). In countries where terminations of pregnancy are illegal, no terminations are recorded. However, in countries where terminations are legal, a reliable surveillance system may not be able to include these events and they will be underreported in these data. Part of the variation in prevalence could reflect true geographical differences in CCHD occurrence due to either genetic predisposition or the frequency of risk factors such as pre-existing maternal diabetes, maternal obesity, use of teratogenic drugs and smoking [18-22]

A second finding was that, whereas the total prevalence varied considerably among programs, the relative distribution of CCHD types was similar. For example, HLHS, CoA, TOF and DTGA were consistently among the most prevalent CCHD (Supplementary Table S2). The exception was India-Chennai, which deviated from the other programs likely because of the exclusively prenatal nature of that program.

A third notable finding was the variation and patterns of prenatal detection (Table 5). Although in all regions second trimester scans are offered as part of standard obstetric care, prenatal detection by program varied from 13% in Slovak Republic to 87% in France- Rhone Alpes, suggesting a role of policies, technical expertise, scanning protocols and practice related to prenatal screening. Even the two programs in the southeastern U.S., Arkansas and Atlanta, Georgia, had widely disparate prenatal detection proportions. The difference in prenatal detection of CCHD between these two programs is consistent with previous reports, which have shown geographic variations in the Unites States,

For peer review only - http://bmjopen.bmj.com/site/about/guidelines.xhtml

ranging from 11.8% to 53.4% [23]. Prenatal detection was more frequent for clinically complex cases (e.g., those with a syndrome or multiple congenital anomalies). This finding, reported also in other studies [15,24], likely reflects a greater intensity of fetal examination when any anomaly is identified prenatally. Prenatal detection was also higher for CCHD with primary or significant involvement of the ventricles, such as HLHS and SV, compared to CCHD in which either additional outflow tract views on fetal ultrasound are required (e.g., DTGA), or the defects are objectively harder to identify (e.g., TAPVR, CoA, and AoS). In addition, other studies have suggested that a postnatal diagnosis is more common for CCHD that require a view on fetal ultrasound other than a 4-chamber view, lesions that are isolated (e.g., absence of another organ system anomaly) or in a setting of poverty or lower population density community [25]. These findings taken together highlight the crucial role of policies, training, and access in driving the rates of prenatal diagnosis in the population.

The proportion of prenatally detected CCHD cases significantly increased over time in most programs (Table 4). The specific patterns varied among programs. For example, in the Northern Netherlands a sharp increase in prenatal detection coincided with the introduction of the prenatal screening program in 2007 (including a 20 week anomaly scan) [26,27], whereas in other programs the increase was more gradual. Increasing trends in prenatal diagnosis were also observed in other studies [23,28-31] and have been variably attributed to improvements in ultrasound technology as well as policies and recommendations pertaining to examination of the fetal anatomy [32-34]. For example, in 2006 the International Society for Ultrasound in Obstetrics and Gynaecology issued a guideline that recommended adding the outflow tract view to the basic 4-chamber view [35].

We examined the patterns of prenatal diagnosis in relation to TOPFA proportions. In programs where such terminations are legal, TOPFA occurred in less than 1 to 35% of CCHD cases. Two patterns seemed to emerge. In some programs such as USA-Arkansas and Slovak Republic, low TOPFA proportions co-occur with a low proportion of prenatal diagnosis. And second, clinically complex cases (e.g., associated with other extracardiac anomalies or syndromic cases) seemed to be prenatally diagnosed more often (Figure 3) – though the relation between clinical complexity and TOPFA was less clear. Pregnancy outcome is not a direct function of prenatal diagnosis. For example, factors that can influence the TOPFA proportion after prenatal diagnosis may not only be social or cultural (for instance acceptance of TOPFA), but also include the legal gestational age limit for pregnancy termination and the extent to which TOPFA are reported to or captured in the health care databases.

Finally, neonatal mortality also varied regionally. The study did not specifically assess the system or personal factors potentially associated with such variation, such as gestational age at birth or

For peer review only - http://bmjopen.bmj.com/site/about/guidelines.xhtml
For peer review only

birthweight. However, we noted that the neonatal mortality was highest in Malta and Argentina where termination of pregnancy is not allowed and prenatal detection of CCHD is low. The lowest neonatal mortality was found in countries where the TOPFA proportions were highest. These findings, though not conclusive, suggest two possibilities. First, prenatal detection might help improve the care of babies with CCHD, by allowing for a better plan of care at birth when compared to the unanticipated urgency at birth if no prenatal diagnosis was made [12,13]. Second, terminations of pregnancy may disproportionately include the anatomically more severe cases (even within the same CCHD type), such that the overall survival is skewed towards what might be only an apparent improvement in outcomes [36].

Strengths and weaknesses of the study

The study has several strengths, including the large sample of the CCHD cohort (>18,000 cases), and the systematic nature of case ascertainment whether through population-based or hospital-based programs. Including programs from areas with different policies and health care systems allowed us a wider view of the interrelated factors that can influence reported prevalence, ascertainment, and prenatal diagnosis. Programs submitted individual case records that were centrally reviewed by clinicians with expertise in genetics (LDB, JEHB) and pediatric cardiology (LDB). This review aimed at harmonizing the CCHD diagnoses (e.g., cases with more than one CCHD code were systematically assigned a primary diagnosis) as well as the clinical classification as isolated, MCA, or syndromic case. The study also has limitations. The quality and completeness of the data submitted centrally depends on the program's methods related to data collection, coding, and classification (e.g., the degree to which clinical staff is involved in these processes). Also, we did not have details on the severity of each CCHD case, which may have contributed to variation across programs. For example, the clinical presentation of lesions such as AoS and COA can range from mild (e.g., not readily identifiable prenatally, or clinically at birth) to severe (e.g., a truly critical condition in the neonatal period). These variations would influence a program's ability to detect these conditions early in life or prenatally and would therefore affect findings such as the total prevalence and the proportion of cases prenatally diagnosed. A last limitation is the challenge and variability in ascertainment of pregnancies that ended in a termination.

Meaning of the study: possible mechanisms and implications for clinicians or policymakers

Ultimately, these findings, together with prior reports from the literature, have both public health and clinical implications for the care and prevention of CCHD. First, the high prevalence (1 in 500 births) underscores the universal need to address primary prevention and care of CCHD aggressively. Care in particular could be enhanced with earlier diagnosis. In this regard, prenatal diagnosis can

For peer review only - http://bmjopen.bmj.com/site/about/guidelines.xhtml

complement pulse oximetry newborn screening, and compared to the latter, allow for more time and hence more thoughtful management decisions by well-informed families and clinicians [37,38].

The increasing trends in prenatal diagnosis rates also highlight the potential for significant changes in the epidemiology and clinical outcomes of CCHD. Although the magnitude of these trends vary in the included programs, the potential implications are vast. Prenatal diagnosis may continue to influence the reported prevalence at birth as well as the outcomes (e.g., morbidity, survival) by a combination of more complete and timely detection and, to a varying extent, its influence on rates of terminations of pregnancy for fetal anomaly. The results of this study demonstrate the value of ongoing surveillance of CCHD in this changing environment.

Tracking and evaluating the patterns of CCHD occurrence is also important in the quest to discover the causes of these severe conditions. For example, in etiological studies, it is particularly important to include all affected fetuses, as stillbirths and terminations of pregnancy are more likely to be overrepresented in more severe cases. Failing to include such cases would limit the range and possibly skew the findings.

Finally, ongoing monitoring of the CCHD cohort, from pregnancy onwards, is important for researchers to appropriately evaluate long-term outcomes and track the burden of disease on population health.

Important questions remain. Is prenatal diagnosis improving population health? In an era of improving (and often more costly) diagnostic technology, are current systems increasing rather than eliminating potential health disparities? Are we providing the most current information about occurrence and outcomes to clinicians and families for appropriate counseling in the presence of a prenatally detected CCHD? Answering such questions requires a joint effort of epidemiologists and clinicians generating high quality information and tracking such data over time. Leveraging existing programs, data sharing and central clinical review and analysis may enhance efficiencies and inform these questions. International networks such as the ICBDSR, the National Birth Defects Prevention Network, and EUROCAT European surveillance of congenital anomalies can help provide the data, the analytic capacity, and a long term vision for sustained , accurate and timely monitoring of the health impact of CCHD, as a basis for interventions aimed at improving primary prevention and care.

For peer review only

For peer review only - http://bmjopen.bmj.com/site/about/guidelines.xhtml

Footnotes

Contributors LDB conceived the study. MKB and LDB developed the protocol and supervised the study. MKB, LDB, and SK conducted the data analysis. LDB and JEHB reviewed the clinical classification of cases. MKB and LDB wrote the first draft of the article. EA, GC, JC, HEKdW, MG, BG, SL, WNN, AP, AR, SC, AS, ES, GT, DT, PM curated and submitted the case data from their programs. All coauthors made substantial contributions to the conduct of the study, interpretation of results and critical revisions of the manuscript. Program directors are guarantors of the integrity of the data submitted for central analysis. MKB and LDB are overall guarantors.

Funding This research received no specific grant from any funding agency in the public, commercial or not-for-profit sectors. The programs participating in this study may receive local funding. The program in Czech Republic is supported by Ministry of Health of the Czech Republic, grant nr. AZV 17-29622A.

Competing interests none declared. All authors have completed the ICMJE uniform disclosure form at www.icmje.org/coi_disclosure.pdf

Patient consent not required. This was a pooled analysis of de-identified data from public health surveillance programs.

Provenance and peer review not commissioned; externally peer reviewed.

Data sharing statement The data used in this study are proprietary of the respective programs and cannot be shared. Use of data collected by the program members of the ICBDSR can be requested by contacting the ICBDSR through centre@icbdsr.org.

Acknowledgements We thank each ICBDSR member program's staff for providing and processing the information on the cases with critical congenital heart defects and on the characteristics of their program.

Disclaimer The findings and conclusions in this report are those of the authors and do not necessarily represent the official position of the Centers for Disease Control and Prevention.

For peer review only

For peer review only - http://bmjopen.bmj.com/site/about/guidelines.xhtml

References

1 Dolk H, Loane M, Garne E, et al. Congenital heart defects in Europe: prevalence and perinatal mortality, 2000 to 2005, *Circulation* 2011;123:841-9.

2 Reller MD, Strickland MJ, Riehle-Colarusso T, et al. Prevalence of congenital heart defects in metropolitan Atlanta, 1998-2005, *J Pediatr* 2008;153:807-13

3 Oster ME, Lee KA, Honein MA, et al. Temporal trends in survival among infants with critical congenital heart defects, *Pediatrics* 2013;131:1502

4 Mackie AS, Tran DT, Marelli AJ, et al. Cost of congenital heart disease hospitalizations in Canada: A population-based study, *Can J Cardiol* 2017;33:792-8

5 Arth AC, Tinker SC, Simeone RM, et al. Inpatient hospitalization costs associated with birth defects among persons of all ages - United States, 2013, *MMWR Morb Mortal Wkly Rep* 2017;66:41-6

6 Boneva RS, Botto LD, Moore CA, et al. Mortality associated with congenital heart defects in the United States: trends and racial disparities, 1979-1997, *Circulation* 2001;103:2376-81

7 Gilboa SM, Salemi JL, Nembhard WN, et al. Mortality resulting from congenital heart disease among children and adults in the United States, 1999 to 2006, *Circulation* 2010;122:2254-63

8 Jortveit J, Eskedal L, Hirth A, et al. Sudden unexpected death in children with congenital heart defects, *Eur Heart J* 2016;37:621-6

9 Knowles RL, Bull C, Wren C, et al. Mortality with congenital heart defects in England and Wales, 1959-2009: exploring technological change through period and birth cohort analysis, *Arch Dis Child* 2012;97:861-5

10 Blyth M, Howe D, Gnanapragasam J, et al. The hidden mortality of transposition of the great arteries and survival advantage provided by prenatal diagnosis, *BJOG* 2008;115:1096-100

11 Bonnet D, Coltri A, Butera G, et al. Detection of transposition of the great arteries in fetuses reduces neonatal morbidity and mortality, *Circulation* 1999;99:916-8

12 Holland BJ, Myers JA, Woods CR. Prenatal diagnosis of critical congenital heart disease reduces risk of death from cardiovascular compromise prior to planned neonatal cardiac surgery: a meta-analysis, *Ultrasound Obstet Gynecol* 2015;45:631-8

For peer review only

For peer review only - http://bmjopen.bmj.com/site/about/guidelines.xhtml

13 Morris SA, Ethen MK, Penny DJ, et al. Prenatal diagnosis, birth location, surgical center, and neonatal mortality in infants with hypoplastic left heart syndrome, *Circulation* 2014;129:285-92.

14 Tworetzky W, McElhinney DB, Reddy VM, et al. Improved surgical outcome after fetal diagnosis of hypoplastic left heart syndrome, *Circulation* 2001;103:1269-73

15 Pinto NM, Keenan HT, Minich LL, et al. Barriers to prenatal detection of congenital heart disease: a population-based study, *Ultrasound Obstet Gynecol* 2012;40:418-25.

16 Clur SA, Bilardo CM. Early detection of fetal cardiac abnormalities: how effective is it and how should we manage these patients? *Prenat Diagn* 2014;34:1235-45

17 Egbe A, Uppu S, Lee S, et al. Changing prevalence of severe congenital heart disease: a population-based study, *Pediatr Cardiol* 2014;35:1232-8

18 Gilboa SM, Correa A, Botto LD, et al. Association between prepregnancy body mass index and congenital heart defects, *Am J Obstet Gynecol* 2010;202:51.e1-e10.

19 Baardman ME, Kerstjens-Frederikse WS, Corpeleijn E, et al. Combined adverse effects of maternal smoking and high body mass index on heart development in offspring: evidence for interaction? *Heart* 2012;98:474-9

20 Hoang TT, Marengo LK, Mitchell LE, et al. Original findings and updated meta-analysis for the association between maternal diabetes and risk for congenital heart disease phenotypes, *Am J Epidemiol* 2017;186:118-28

21 Jenkins KJ, Correa A, Feinstein JA, et al. Noninherited risk factors and congenital cardiovascular defects: current knowledge: a scientific statement from the American Heart Association Council on Cardiovascular Disease in the Young: endorsed by the American Academy of Pediatrics, *Circulation* 2007;115:2995-3014

22 Mills JL, Troendle J, Conley MR, et al. Maternal obesity and congenital heart defects: a population-based study, *Am J Clin Nutr* 2010;91:1543-9

23 Quartermain MD, Pasquali SK, Hill KD, et al. Variation in prenatal diagnosis of congenital heart disease in infants, *Pediatrics* 2015;136:e378-85

24 Wong SF, Chan FY, Cincotta RB, et al. Factors influencing the prenatal detection of structural congenital heart diseases, *Ultrasound Obstet Gynecol* 2003;21:19-25

For peer review only

For peer review only - http://bmjopen.bmj.com/site/about/guidelines.xhtml

25 Hill GD, Block JR, Tanem JB, et al. Disparities in the prenatal detection of critical congenital heart disease, *Prenat Diagn* 2015;35:859-63

26 Baardman ME, du Marchie Sarvaas, G J, de Walle HE, et al. Impact of introduction of 20-week ultrasound scan on prevalence and fetal and neonatal outcomes in cases of selected severe congenital heart defects in The Netherlands, *Ultrasound Obstet Gynecol* 2014;44:58-63

27 van Velzen CL, Clur SA, Rijlaarsdam ME, et al. Prenatal detection of congenital heart disease--results of a national screening programme, *BJOG* 2016;123:400-7

28 Chew C, Stone S, Donath SM, et al. Impact of antenatal screening on the presentation of infants with congenital heart disease to a cardiology unit, *J Paediatr Child Health* 2006;42:704-8

29 Corcoran S, Briggs K, O'Connor H, et al. Prenatal detection of major congenital heart disease - optimising resources to improve outcomes, *Eur J Obstet Gynecol Reprod Biol* 2016;203:260-3

30 van Velzen CL, Ket JCF, van de Ven, P M, et al. Systematic review and meta-analysis of the performance of second-trimester screening for prenatal detection of congenital heart defects, *Int J Gynaecol Obstet* 2018;140:137-45

31 Lytzen R, Veijlstrup N, Bjerre J, et al. Live born major congenital heart disease in Denmark; incidence, detection rate and termination of pregnancy rate from 1996-2013, *JAMA Cardiol*. 2018;3(9):829-837

32 Komisar J, Srivastava S, Geiger M, et al. Impact of changing indications and increased utilization of fetal echocardiography on prenatal detection of congenital heart disease, *Congenit Heart Dis* 2017;12:67-73

33 Ogge G, Gaglioti P, Maccanti S, et al. Prenatal screening for congenital heart disease with four-chamber and outflow-tract views: a multicenter study, *Ultrasound Obstet Gynecol* 2006;28:779-84

34 Sklansky MS, Berman DP, Pruetz JD, et al. Prenatal screening for major congenital heart disease: superiority of outflow tracts over the 4-chamber view, *J Ultrasound Med* 2009;28:889-99

35 Anonymous. Cardiac screening examination of the fetus: guidelines for performing the ‘basic’ and ‘extended basic’ cardiac scan, *Ultrasound Obstet Gynecol* 2006;27:107-13

36 Boyle B, Addor MC, Arriola L, et al. Estimating global burden of disease due to congenital anomaly: an analysis of European data, *Arch Dis Child Fetal Neonatal Ed* 2018;103:F22-F28

For peer review only

For peer review only - http://bmjopen.bmj.com/site/about/guidelines.xhtml

37 Bratt EL, Jarvholm S, Ekman-Joelsson BM, et al. Parent's experiences of counseling and their need for support following a prenatal diagnosis of congenital heart disease--a qualitative study in a Swedish context, *BMC Pregnancy Childbirth* 2015;15:171

38 Thakur V, Dutil N, Schwartz SM, et al. Impact of prenatal diagnosis on the management and early outcome of critical duct-dependent cardiac lesions, *Cardiol Young* 2018;28:548-53

For peer review only

For peer review only - http://bmjopen.bmj.com/site/about/guidelines.xhtml

Figures

Figure 1. Total prevalence and live birth prevalence (per 10,000 births) with 95% confidence intervals for 12 CCHD types, by program, International Clearinghouse for Birth Defects Surveillance and Research (ICBDSR) Critical Congenital Heart Defects (CCHD) Prenatal Diagnosis study 2000-2014.

[1] ICBDSR Programs, ordered by descending total prevalence, contributed data for different years within this time period, see table 1.
[2] India Chennai program is not included in prevalence estimates because for this exclusively prenatal program the denominator data (total births, total live births) are unavailable.

Figure 2. Proportion prenatally diagnosed and proportion of live births among all CCHD cases by program, International Clearinghouse for Birth Defects Surveillance and Research (ICBDSR) Critical Congenital Heart Defects (CCHD) Prenatal Diagnosis study 2000-2014.

[1] ICBDSR Programs, (ordered by descending prenatal diagnosis proportion), contributed data for different years within this time period, see table 1.
[2] India Chennai is not included in the figure because as an exclusively prenatal diagnosis program, all cases by design were prenatally diagnosed, and information on outcome of pregnancy is missing in the majority of cases.

Figure 3. Proportion of prenatally diagnosed CCHD cases according to clinical presentation and by program International Clearinghouse for Birth Defects Surveillance and Research (ICBDSR) Critical Congenital Heart Defects (CCHD) Prenatal Diagnosis study 2000-2014.

[1] Programs (ordered by descending prenatal detection proportion) contributed data for different years within this time period, see table 1. India Chennai is a prenatal diagnosis only program.
Abbreviations: MCA multiple congenital anomalies

Figure 4. First month mortality in live birth cases with selected CCHD by program, International Clearinghouse for Birth Defects Surveillance and Research (ICBDSR) Critical Congenital Heart Defects (CCHD) Prenatal Diagnosis study 2000-2014.

[1] ICBDSR programs (ordered by descending first month mortality) contributed data for different years within this time period, see table 1.
[2]India-Chennai and Canada are not included in the graph: Pregnancy outcomes in India-Chennai are poorly reported and Canada reported on mortality one year after birth, not specified in first week or first month mortality.

For peer review only

For peer review only - http://bmjopen.bmj.com/site/about/guidelines.xhtml

Supplementary Tables and Appendix

Table S1. Total prevalence per 10,000 births per year per program for selected CCHD, International Clearinghouse for Birth Defects Surveillance and Research (ICBDSR) Critical Congenital Heart Defects (CCHD) Prenatal Diagnosis study 2000-2014.

Table S2. Distribution of CCHD types per program (%). The proportions add to 100% per program, International Clearinghouse for Birth Defects Surveillance and Research (ICBDSR) Critical Congenital Heart Defects (CCHD) Prenatal Diagnosis study 2000-2014.

Appendix. Assigning a main diagnosis of critical congenital heart defects (CCHD).

For peer review only

For peer review only - http://bmjopen.bmj.com/site/about/guidelines.xhtml

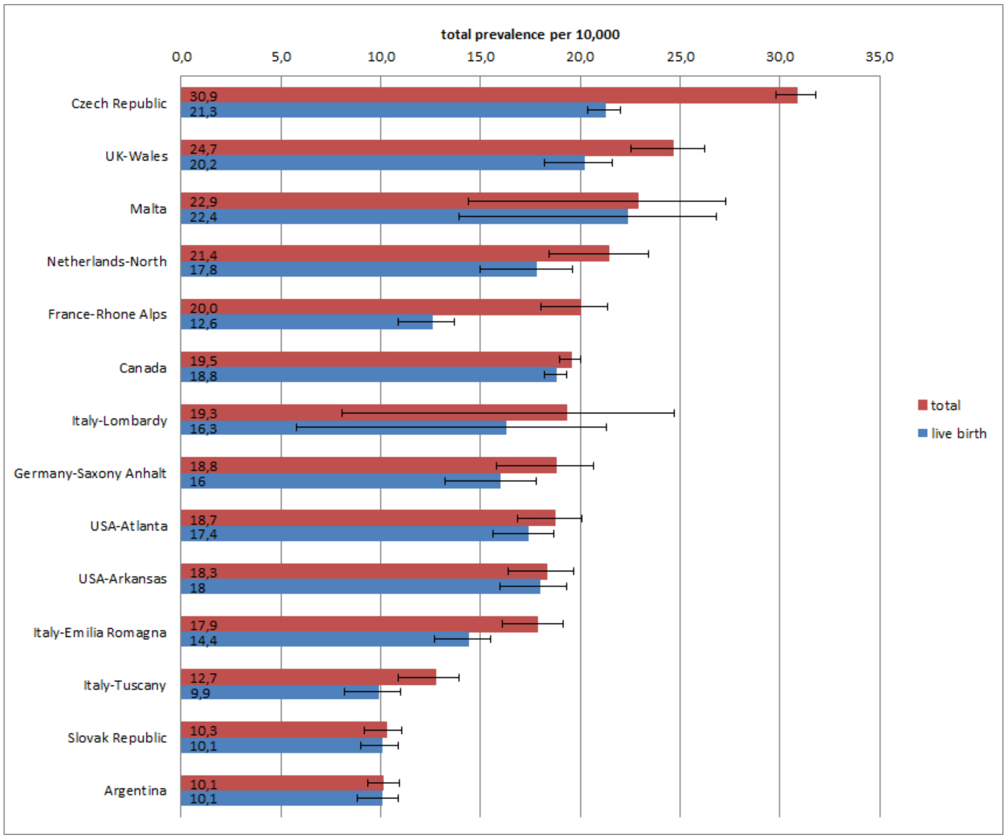

Figure 1. Total prevalence and live birth prevalence (per 10,000 births) with 95% confidence intervals for 12 CCHD types, by program, International Clearinghouse for Birth Defects Surveillance and Research (ICBDSR) Critical Congenital Heart Defects (CCHD) Prenatal Diagnosis study 2000-2014.

1 ICBDSR Programs, ordered by descending total prevalence, contributed data for different years within this time period, see table 1.

2 India Chennai program is not included in prevalence estimates because for this exclusively prenatal program the denominator data (total births, total live births) are unavailable.

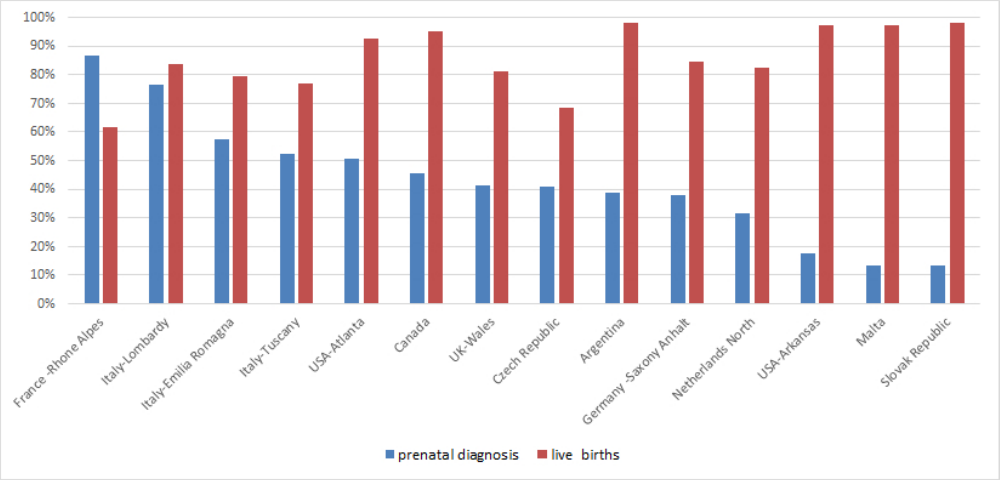

Figure 2. Proportion prenatally diagnosed and proportion of live births among all CCHD cases by program, International Clearinghouse for Birth Defects Surveillance and Research (ICBDSR) Critical Congenital Heart Defects (CCHD) Prenatal Diagnosis study 2000-2014.

1 ICBDSR Programs, (ordered by descending prenatal diagnosis proportion), contributed data for different years within this time period, see table 1.
2 India Chennai is not included in the figure because as an exclusively prenatal diagnosis program, all cases by design were prenatally diagnosed, and information on outcome of pregnancy is missing in the majority of cases.

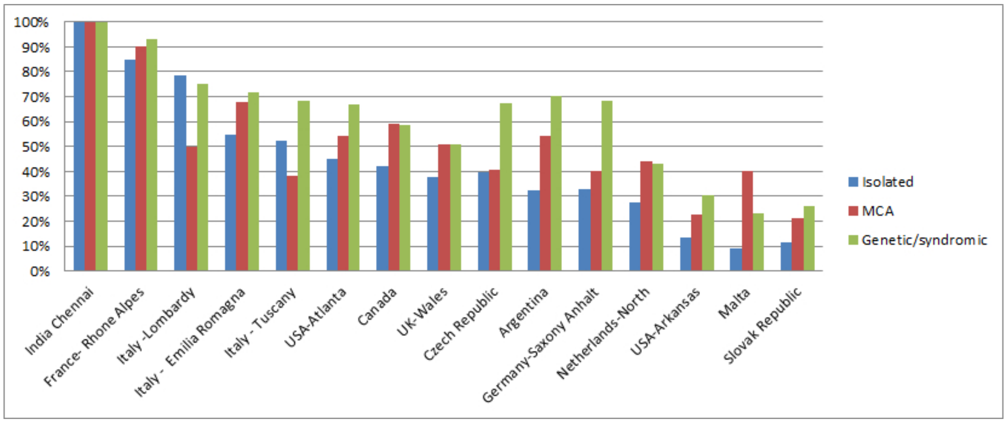

Figure 3. Proportion of prenatally diagnosed CCHD cases according to clinical presentation and by program International Clearinghouse for Birth Defects Surveillance and Research (ICBDSR) Critical Congenital Heart Defects (CCHD) Prenatal Diagnosis study 2000-2014.
1 Programs (ordered by descending prenatal detection proportion) contributed data for different years within this time period, see table 1. India Chennai is a prenatal diagnosis only program.
Abbreviations: MCA multiple congenital anomalies

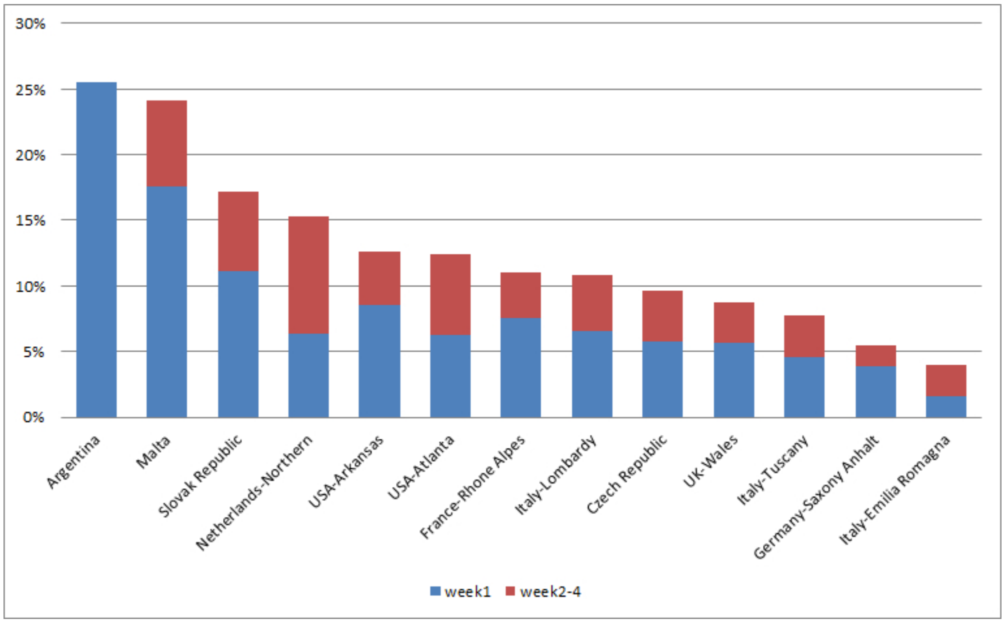

Figure 4. First month mortality in live birth cases with selected CCHD by program, International Clearinghouse for Birth Defects Surveillance and Research (ICBDSR) Critical Congenital Heart Defects (CCHD) Prenatal Diagnosis study 2000-2014.
1 ICBDSR programs (ordered by descending first month mortality) contributed data for different years within this time period, see table 1.
2India-Chennai and Canada are not included in the graph: Pregnancy outcomes in India-Chennai are poorly reported and Canada reported on mortality one year after birth, not specified in first week or first month mortality.

Supplementary Table S1. Total prevalence per 10,000 births per year per program for selected CCHD, , International Clearinghouse for Birth Defects Surveillance and Research (ICBDSR) Critical Congenital Heart Defects (CCHD) Prenatal Diagnosis study 2000-2014.[1,2]

Program by geographic region	2000	2001	2002	2003	2004	2005	2006	2007	2008	2009	2010	2011	2012	2013	2014	**Totals**	Trend
Czech Republic	33.0	34.9	32.5	33.7	37.4	29.7	25.6	37.9	33.1	28.8	33.4	30.3	27.3	16.0		**30.9**	\
UK-Wales		23.6	23.9	30.2	21.4	24.9	23.0	30.2	25.8	24.6	22.3	23.1	23.3			**24.7**	-
Malta		30.3	15.3	7.4	28.2	25.9	20.6	35.9	23.7	26.3	9.9	25.5	25.8			**22.9**	-
Netherlands-Northern		19.0	19.6	22.4	30.8	14.6	20.9	20.9	18.3	20.9	27.2	16.8	26.4			**21.4**	~
France-Rhone Alpes							18.9	16.5	16.9	19.6	17.7	23.0	26.6			**20.0**	/
Canada					19.3	20.9	19.2	19.6	19.4	18.8	18.6	20.6	20.4	19.2	19.2	**20.1**	-
Italy-Lombardy										22.1	16.4					**19.3**	nc
Germany-Saxony Anhalt		13.7	19.2	17.0	16.0	25.4	18.2	14.3	28.0	15.1	17.2	19.5	21.8			**18.8**	~
USA-Atlanta		18.9	24.1	19.0	18.0	20.5	18.0	17.0	14.9							**18.7**	\
USA-Arkansas		15.8	20.7	17.1	15.2	13.4	19.6	19.5	18.5	21.5	21.8					**18.3**	/
Italy-Emilia Romagna		18.3	16.5	18.4	18.7	18.5	16.2	15.6	14.3	17.6	17.2	24.5	18.9			**17.9**	-
Italy-Tuscany		15.1	9.0	12.2	12.7	9.8	15.5	11.6	13.8	13.0	18.0	12.8	8.6			**12.7**	-
Slovak Republic		8.9	12.5	10.2	11.9	10.2	10.0	7.3	7.8	10.2	12.7	9.5	12.0			**10.3**	-
Argentina														9.8	10.4	**10.1**	nc

[1]ICBDSR Programs contributed data for different years within this time period, see table 1.
[2]India Chennai program is not included in prevalence estimates because for this exclusively prenatal program the denominator data (total births, total livebirths) are unavailable.
/ significant increasing trend, \ significant decreasing trend, ~heterogeneous prevalence, - no trend, nc not calculated because of too few years

For peer review only

For peer review only - http://bmjopen.bmj.com/site/about/guidelines.xhtml

Supplementary Table S2 Distribution of CCHD types per program (%). The proportions add to 100% per program, , International Clearinghouse for Birth Defects Surveillance and Research (ICBDSR) Critical Congenital Heart Defects (CCHD) Prenatal Diagnosis study 2000-2014.[1]

	LVOTO			Conotruncal					RVOTO			
Program by geographic region	HLH	COA	AoS	TOF	DTGA	DORV	PTA	IAA	PulmA	TriA/HRH	SV	TAPVR
UK-Wales	13.5	20.1	10.2	14.4	13.7	5.2	4.5	3.3	5.2	2.1	3.5	4.5
Germany-Saxony Anhalt	14.3	23.7	6.9	17.3	17.6	3.8	3.3	1.3	4.6	1.8	2.3	3.1
Netherlands-Northern	15.5	17.0	10.3	15.5	17.2	5.2	1.9	2.1	6.1	2.1	4.0	3.1
France-Rhone Alpes	22.9	10.9	3.8	17.7	19.9	5.1	4.1	0.4	4.0	4.5	5.5	1.2
Italy-Emilia Romagna	14.6	17.5	3.1	21.1	16.1	6.9	4.0	0.9	4.4	4.7	4.4	2.3
Italy-Lombardy	16.4	25.5	5.5	25.5	7.3	7.3	1.8	0.0	3.6	3.6	3.6	0.0
Italy-Tuscany	18.4	16.0	3.8	19.5	21.1	6.2	2.4	0.4	3.5	3.1	4.2	1.3
Malta	18.0	18.0	5.4	14.4	20.7	2.7	1.8	1.8	5.4	3.6	7.2	0.9
Czech Republic	10.6	18.5	15.8	12.4	11.8	11.0	4.4	2.5	6.0	1.9	2.8	2.5
Slovak Republic	22.1	11.2	7.6	17.9	10.0	7.0	9.0	1.6	4.5	2.5	4.4	2.2
Canada	9.6	25.2	7.5	20.0	15.1	6.2	2.5	0.5	3.9	2.3	1.9	5.2
USA-Arkansas	17.5	25.6	10.7	4.7	13.3	6.0	3.5	2.9	3.7	2.5	4.3	5.4
USA-Atlanta	11.9	21.4	5.7	25.8	10.9	2.8	4.5	1.8	3.1	3.4	4.9	3.9
Argentina	18.6	15.1	2.6	14.8	14.4	4.9	4.4	3.0	3.4	2.5	11.3	4.9
India-Chennai	15.4	1.5	4.0	25.4	10.2	11.5	4.2	0.0	0.8	6.0	19.5	1.5

[1]ICBDSR programs contributed data for different years within this time period, see table 1.
Abbreviations: LVOTO left ventricular outflow tract obstruction, RVOTO right ventricular outflow tract obstruction, SV single ventricle, TAPVR total anomalous pulmonary venous return, CCHD critical congenital heart defects, HLHS hypoplastic left heart syndrome, COA coarctation of the aorta, AoS aortic valve stenosis, TOF tetralogy of Fallot, DTGA d-transposition of great arteries, DORV double outlet right ventricle, PTA persistent truncus arteriosus, IAA interrupted aortic arch, PulmA pulmonary valve atresia with intact ventricular septum, TriA/HRH tricuspid valve atresia / hypoplastic right heart, SV single ventricle, TAPVR total anomalous pulmonary venous return

supplementary tables and appendix_ Prenatal Diagnosis and Prevalence of Critical Congenital Heart Defects: an International Retrospective Cohort Study_MK Bakker et al. 2019

For peer review only

Appendix. Assigning a main diagnosis of critical congenital heart defects (CCHD)

In this study, programs submitted cases with at least one of 12 diagnoses considered to be consistent with CCHD. These diagnoses (identified by their ICD9 or ICD10 codes) were hypoplastic left heart syndrome (HLHS), coarctation of the aorta (COA), aortic valve stenosis (AoS), tetralogy of Fallot (TOF), d-transposition of great arteries (DTGA), double outlet right ventricle (DORV), persistent truncus arteriosus (PTA), interrupted aortic arch (IAA), pulmonary valve atresia with intact ventricular septum (PulmA), tricuspid valve atresia / hypoplastic right heart (TriA/HRH), single ventricle (SV) and total anomalous pulmonary venous return (TAPVR).

Most cases had just one of these diagnoses ('#CCHD dx' in table below), as noted in the column '# cases'. For the few cases with more than one CCHD code, a single CCHD code was assigned, using the system below for consistency. The rationale for the algorithm was as follows:

a) assign where possible the more severe diagnosis within the same spectrum. For example, in the case of left sided obstructive anomalies, the hierarchy was HLHS > CoA > AoS
b) assign the more dominant condition when diagnoses were not in the same spectrum. For example, in the case of IAA and several other types of CCHD (see below), the diagnosis of IAA prevailed. In the case of HLHS, a CCHD that is both severe clinically as well as easily identifiable at prenatal ultrasound examination, this diagnosis took precedence over several other types of CCHD (see table below). In the case of SV, some CCHD combinations were especially complex, so that the SV group ended up including fairly straightforward conditions such as double inlet left ventricle as well as more complex conditions, in which the SV morphology was joined by several other CCHD lesions.

Two points are worth noting. First, the approach used here was developed by the study's clinical team with expertise in medical genetics and pediatric cardiology. However, some combinations of CCHD codes, there could be disagreements among experts as to which main diagnosis to assign.. Examples include the placement of phenotypes that include tricuspid and pulmonary atresia, or the more complex forms of hypoplastic left heart. Ideally, a more granular approach might be preferable, to avoid grouping somewhat heterogeneous lesions. However, too many small groups would make the analysis unmanageable and a reasonable balance between 'splitting' and 'lumping' had to be achieved. In this case, the decision was made to be systematic (assignment based on specific code combinations) and explicit (full assignment table provided), to improve the clarity and reproducibility of the study. Second, as it is clear from the table, the cases with multiple CCHD codes, and particularly those with more complex combinations, accounted each for very few cases , so any disagreement on the assignment of such cases would likely have a minimal effect of the overall findings of the study.

For peer review only - http://bmjopen.bmj.com/site/about/guidelines.xhtml

# CCHD dx	AoS	COA	IAA	DORV	SV	HLHS	PulmA	TriA	TAPVR	DTGA	TOF	PTA	# cases	Final Assignment
1	1	0	0	0	0	0	0	0	0	0	0	0	1792	01.AoS
1	0	1	0	0	0	0	0	0	0	0	0	0	3945	02.COA
2	1	1	0	0	0	0	0	0	0	0	0	0	67	02.COA
1	0	0	1	0	0	0	0	0	0	0	0	0	249	03.IAA
2	0	1	1	0	0	0	0	0	0	0	0	0	7	03.IAA
2	1	0	1	0	0	0	0	0	0	0	0	0	4	03.IAA
2	0	0	1	0	0	0	0	1	0	0	0	0	3	03.IAA
2	0	0	1	0	0	0	0	0	1	0	0	0	2	03.IAA
2	0	0	1	1	0	0	0	0	0	0	0	0	2	03.IAA
3	1	0	1	1	0	0	0	0	0	0	0	0	2	03.IAA
2	0	0	1	0	0	0	0	0	0	0	1	0	1	03.IAA
2	0	0	1	0	0	0	1	0	0	0	0	0	1	03.IAA
3	0	1	1	1	0	0	0	0	0	0	0	0	1	03.IAA
1	0	0	0	1	0	0	0	0	0	0	0	0	1361	04.DORV
2	0	1	0	1	0	0	0	0	0	0	0	0	24	04.DORV
2	0	0	0	1	0	0	0	0	0	0	1	0	9	04.DORV
2	1	0	0	1	0	0	0	0	0	0	0	0	2	04.DORV
1	0	0	0	0	1	0	0	0	0	0	0	0	632	05.SV
2	0	0	0	0	1	0	0	0	0	1	0	0	29	05.SV
2	0	1	0	0	1	0	0	0	0	0	0	0	11	05.SV
2	0	0	0	0	1	0	0	1	0	0	0	0	10	05.SV
2	0	0	0	0	1	0	1	0	0	0	0	0	7	05.SV
3	0	1	0	0	1	0	0	0	0	1	0	0	7	05.SV
2	0	0	0	1	1	0	0	0	0	0	0	0	6	05.SV
2	0	0	0	0	1	0	0	0	0	0	0	1	4	05.SV
2	0	0	0	0	1	0	0	0	1	0	0	0	4	05.SV
2	0	0	1	0	1	0	0	0	0	0	0	0	4	05.SV
2	1	0	0	0	1	0	0	0	0	0	0	0	4	05.SV
3	0	0	0	1	1	0	0	0	0	1	0	0	4	05.SV
3	0	0	0	0	1	0	0	0	1	1	0	0	2	05.SV

supplementary tables and appendix_ Prenatal Diagnosis and Prevalence of Critical Congenital Heart Defects: an International Retrospective Cohort Study_MK Bakker et al. 2019

2	0	0	0	0	1	0	0	0	0	0	1	0	1	05.SV
3	0	0	0	0	1	0	0	0	0	1	1	0	1	05.SV
3	0	0	0	0	1	0	0	1	0	1	0	0	1	05.SV
3	0	0	0	0	1	0	1	0	0	0	0	1	1	05.SV
3	0	0	0	0	1	0	1	0	0	1	0	0	1	05.SV
3	0	0	0	1	1	0	0	0	1	0	0	0	1	05.SV
3	0	0	0	1	1	0	1	0	0	0	0	0	1	05.SV
3	0	0	1	0	1	0	0	0	0	1	0	0	1	05.SV
3	0	1	0	0	1	0	0	1	0	0	0	0	1	05.SV
3	0	1	0	1	1	0	0	0	0	0	0	0	1	05.SV
3	1	0	0	0	1	0	0	0	0	1	0	0	1	05.SV
4	0	0	0	1	1	0	1	1	0	0	0	0	1	05.SV
4	1	1	0	0	1	0	0	0	0	1	0	0	1	05.SV
1	0	0	0	0	0	1	0	0	0	0	0	0	2386	06.HLH
2	0	1	0	0	0	1	0	0	0	0	0	0	44	06.HLH
2	1	0	0	0	0	1	0	0	0	0	0	0	26	06.HLH
2	0	0	0	1	0	1	0	0	0	0	0	0	18	06.HLH
2	0	0	0	0	0	1	0	0	1	0	0	0	8	06.HLH
2	0	0	0	0	0	1	0	0	0	1	0	0	7	06.HLH
2	0	0	1	0	0	1	0	0	0	0	0	0	6	06.HLH
2	0	0	0	0	1	1	0	0	0	0	0	0	4	06.HLH
3	0	1	0	1	0	1	0	0	0	0	0	0	4	06.HLH
4	0	1	0	1	1	1	0	0	0	0	0	0	3	06.HLH
2	0	0	0	0	0	1	0	0	0	0	0	1	2	06.HLH
2	0	0	0	0	0	1	1	0	0	0	0	0	2	06.HLH
3	0	0	0	1	0	1	0	0	1	0	0	0	2	06.HLH
3	1	1	0	0	0	1	0	0	0	0	0	0	2	06.HLH
2	0	0	0	0	0	1	0	1	0	0	0	0	1	06.HLH
3	0	0	0	1	0	1	0	0	0	1	0	0	1	06.HLH
3	0	0	1	1	0	1	0	0	0	0	0	0	1	06.HLH
3	0	1	0	0	0	1	0	0	1	0	0	0	1	06.HLH
3	0	1	1	0	0	1	0	0	0	0	0	0	1	06.HLH

supplementary tables and appendix_ Prenatal Diagnosis and Prevalence of Critical Congenital Heart Defects: an International Retrospective Cohort Study_MK Bakker et al. 2019

3	1	0	0	0	1	1	0	0	0	0	0	0	1	06.HLH
4	0	0	0	1	0	1	1	0	0	1	0	0	1	06.HLH
4	0	1	0	0	1	1	0	0	1	0	0	0	1	06.HLH
4	1	1	0	0	0	1	0	1	0	0	0	0	1	06.HLH
4	1	1	0	1	0	1	0	0	0	0	0	0	1	06.HLH
1	0	0	0	0	0	0	1	0	0	0	0	0	1021	07.PulmA
2	0	0	0	1	0	0	1	0	0	0	0	0	9	07.PulmA
2	0	0	0	0	0	0	1	0	1	0	0	0	4	07.PulmA
2	1	0	0	0	0	0	1	0	0	0	0	0	2	07.PulmA
3	0	0	0	1	0	0	1	0	1	0	0	0	1	07.PulmA
4	1	1	0	0	0	0	1	0	1	0	0	0	1	07.PulmA
1	0	0	0	0	0	0	0	1	0	0	0	0	494	08.TriA
2	0	0	0	0	0	0	1	1	0	0	0	0	11	08.TriA
2	1	0	0	0	0	0	0	1	0	0	0	0	4	08.TriA
2	0	0	0	1	0	0	0	1	0	0	0	0	2	08.TriA
2	0	1	0	0	0	0	0	1	0	0	0	0	2	08.TriA
3	0	0	0	0	0	0	1	1	1	0	0	0	1	08.TriA
1	0	0	0	0	0	0	0	0	1	0	0	0	754	09.TAPVR
2	0	0	0	1	0	0	0	0	1	0	0	0	11	09.TAPVR
2	0	1	0	0	0	0	0	0	1	0	0	0	2	09.TAPVR
2	1	0	0	0	0	0	0	0	1	0	0	0	1	09.TAPVR
1	0	0	0	0	0	0	0	0	0	1	0	0	2711	10.DTGA
2	0	1	0	0	0	0	0	0	0	1	0	0	29	10.DTGA
2	0	0	0	1	0	0	0	0	0	1	0	0	15	10.DTGA
2	0	0	0	0	0	0	0	1	0	1	0	0	12	10.DTGA
2	0	0	0	0	0	0	1	0	0	1	0	0	9	10.DTGA
3	0	1	0	1	0	0	0	0	0	1	0	0	8	10.DTGA
3	0	1	0	0	0	0	0	1	0	1	0	0	6	10.DTGA
3	0	0	0	1	0	0	1	0	0	1	0	0	4	10.DTGA
2	0	0	0	0	0	0	0	0	1	1	0	0	3	10.DTGA
3	0	0	0	0	0	0	1	0	1	1	0	0	2	10.DTGA
3	0	0	0	1	0	0	0	0	1	1	0	0	2	10.DTGA

supplementary tables and appendix_ Prenatal Diagnosis and Prevalence of Critical Congenital Heart Defects: an International Retrospective Cohort Study_MK Bakker et al. 2019

2	1	0	0	0	0	0	0	0	0	1	0	0	1	10.DTGA
3	1	0	0	0	0	0	0	0	0	1	1	0	1	10.DTGA
4	0	1	0	1	0	0	0	1	0	1	0	0	1	10.DTGA
1	0	0	0	0	0	0	0	0	0	0	1	0	3415	11.TOF
2	0	0	0	0	0	0	1	0	0	0	1	0	4	11.TOF
2	0	0	0	0	0	0	0	0	1	0	1	0	3	11.TOF
2	0	1	0	0	0	0	0	0	0	0	1	0	2	11.TOF
2	1	0	0	0	0	0	0	0	0	0	1	0	2	11.TOF
3	0	0	0	1	0	0	0	0	1	0	1	0	2	11.TOF
2	0	0	0	0	0	0	0	1	0	0	1	0	1	11.TOF
1	0	0	0	0	0	0	0	0	0	0	0	1	713	12.PTA
2	0	0	1	0	0	0	0	0	0	0	0	1	13	12.PTA
2	1	0	0	0	0	0	0	0	0	0	0	1	3	12.PTA
2	0	0	0	0	0	0	1	0	0	0	0	1	2	12.PTA
2	0	0	0	0	0	0	0	0	0	1	0	1	1	12.PTA
2	0	0	0	0	0	0	0	0	1	0	0	1	1	12.PTA
3	0	0	0	1	0	0	0	0	1	0	0	1	1	12.PTA
3	0	0	1	0	0	0	0	0	0	0	1	1	1	12.PTA

ew only

For peer review only - http://bmjopen.bmj.com/site/about/guidelines.xhtml

STROBE 2007 (v4) checklist of items to be included in reports of observational studies in epidemiology*
Checklist for cohort, case-control, and cross-sectional studies (combined)

Section/Topic	Item #	Recommendation	Reported on page #
**Title and abstract**	1	(*a*) Indicate the study's design with a commonly used term in the title or the abstract	1
		(*b*) Provide in the abstract an informative and balanced summary of what was done and what was found	2
**Introduction**			
Background/rationale	2	Explain the scientific background and rationale for the investigation being reported	4
Objectives	3	State specific objectives, including any pre-specified hypotheses	4
**Methods**			
Study design	4	Present key elements of study design early in the paper	5
Setting	5	Describe the setting, locations, and relevant dates, including periods of recruitment, exposure, follow-up, and data collection	5
Participants	6	(*a*) *Cohort study*—Give the eligibility criteria, and the sources and methods of selection of participants. Describe methods of follow-up<br>*Case-control study*—Give the eligibility criteria, and the sources and methods of case ascertainment and control selection. Give the rationale for the choice of cases and controls<br>*Cross-sectional study*—Give the eligibility criteria, and the sources and methods of selection of participants	5
		(*b*) *Cohort study*—For matched studies, give matching criteria and number of exposed and unexposed<br>*Case-control study*—For matched studies, give matching criteria and the number of controls per case	na
Variables	7	Clearly define all outcomes, exposures, predictors, potential confounders, and effect modifiers. Give diagnostic criteria, if applicable	5
Data sources/ measurement	8*	For each variable of interest, give sources of data and details of methods of assessment (measurement). Describe comparability of assessment methods if there is more than one group	5
Bias	9	Describe any efforts to address potential sources of bias	5/6
Study size	10	Explain how the study size was arrived at	5
Quantitative variables	11	Explain how quantitative variables were handled in the analyses. If applicable, describe which groupings were chosen and why	5
Statistical methods	12	(*a*) Describe all statistical methods, including those used to control for confounding	6
		(*b*) Describe any methods used to examine subgroups and interactions	6
		(*c*) Explain how missing data were addressed	na
		(*d*) *Cohort study*—If applicable, explain how loss to follow-up was addressed<br>*Case-control study*—If applicable, explain how matching of cases and controls was addressed	na

For peer review only

		Cross-sectional study—If applicable, describe analytical methods taking account of sampling strategy	
		(*e*) Describe any sensitivity analyses	na
**Results**			
Participants	13*	(a) Report numbers of individuals at each stage of study—eg numbers potentially eligible, examined for eligibility, confirmed eligible, included in the study, completing follow-up, and analysed	7
		(b) Give reasons for non-participation at each stage	na
		(c) Consider use of a flow diagram	na
Descriptive data	14*	(a) Give characteristics of study participants (eg demographic, clinical, social) and information on exposures and potential confounders	7, tables
		(b) Indicate number of participants with missing data for each variable of interest	table 3
		(c) *Cohort study*—Summarise follow-up time (eg, average and total amount)	na
Outcome data	15*	*Cohort study*—Report numbers of outcome events or summary measures over time	na
		Case-control study—Report numbers in each exposure category, or summary measures of exposure	na
		Cross-sectional study—Report numbers of outcome events or summary measures	7/8
Main results	16	(*a*) Give unadjusted estimates and, if applicable, confounder-adjusted estimates and their precision (eg, 95% confidence interval). Make clear which confounders were adjusted for and why they were included	7, tables and figures
		(*b*) Report category boundaries when continuous variables were categorized	na
		(*c*) If relevant, consider translating estimates of relative risk into absolute risk for a meaningful time period	na
Other analyses	17	Report other analyses done—eg analyses of subgroups and interactions, and sensitivity analyses	na
**Discussion**			
Key results	18	Summarise key results with reference to study objectives	9/10
Limitations	19	Discuss limitations of the study, taking into account sources of potential bias or imprecision. Discuss both direction and magnitude of any potential bias	11
Interpretation	20	Give a cautious overall interpretation of results considering objectives, limitations, multiplicity of analyses, results from similar studies, and other relevant evidence	11
Generalisability	21	Discuss the generalisability (external validity) of the study results	11
**Other information**			
Funding	22	Give the source of funding and the role of the funders for the present study and, if applicable, for the original study on which the present article is based	13

*Give information separately for cases and controls in case-control studies and, if applicable, for exposed and unexposed groups in cohort and cross-sectional studies.

Note: An Explanation and Elaboration article discusses each checklist item and gives methodological background and published examples of transparent reporting. The STROBE checklist is best used in conjunction with this article (freely available on the Web sites of PLoS Medicine at http://www.plosmedicine.org/, Annals of Internal Medicine at http://www.annals.org/, and Epidemiology at http://www.epidem.com/). Information on the STROBE Initiative is available at www.strobe-statement.org.

For peer review only
