# Supplementary material for: Prenatal diagnosis and prevalence of critical congenital heart defects: an international retrospective cohort study
Source: BMJ Open. 2019 Jul 2;9(7):e028139. doi: 10.1136/bmjopen-2018-028139 (PMC6609145; doi:10.1136/bmjopen-2018-028139)
Supplement: Reviewer comments [file bmjopen-2018-028139.reviewer_comments.pdf]

PEER REVIEW HISTORY

BMJ Open publishes all reviews undertaken for accepted manuscripts. Reviewers are asked to complete a checklist review form (**http://bmjopen.bmj.com/site/about/resources/checklist.pdf**) and are provided with free text boxes to elaborate on their assessment. These free text comments are reproduced below.

ARTICLE DETAILS

**TITLE (PROVISIONAL)**	Prenatal Diagnosis and Prevalence of Critical Congenital Heart Defects: an International Retrospective Cohort Study
**AUTHORS**	Bakker, Marian; Bergman, Jorieke; Krikov, Sergey; Amar, Emmanuelle; Cocchi, Guido; Cragan, Janet; de Walle, Hermien; Gatt, Miriam; Groisman, Boris; Liu, Shiliang; Nembhard, WN; Pierini, Anna; Rissmann, Anke; Chidambarathanu, S; Sipek Jr, Antonin; Szabova, Elena; Tagliabue, Giovanna; Tucker, David; Mastroiacovo, Pierpaolo; Botto, Lorenzo

VERSION 1 – REVIEW

**REVIEWER**	Andrea Székely<br>Semmelweis UNIversity Budapest, Hungary
**REVIEW RETURNED**	30-Dec-2018

**GENERAL COMMENTS**	The authors presented a retrospective analysis on the detection, types and fate of embryos with congenital heart disease. They have done a huge work also a cross-sectional analysis. The paper is well written and concise. The discussion is also interesting.

**REVIEWER**	Patrick Jay<br>Washington University School of Medicine, USA
**REVIEW RETURNED**	09-Jan-2019

**GENERAL COMMENTS**	Bakker et al report retrospective analyses of the prenatal detection of critical congenital heart defects (CCHD), the overall birth prevalence and neonatal (1-month) mortality from 15 international birth defect surveillance programs around the world. Wide variation in practice patterns and other undefined factors were associated with variable prenatal detection rates, termination rates, and 1-month mortality between centers. Some programs contributed considerably more years of data and cases than others over different time periods, which makes comparison between centers challenging. Nevertheless, this cohort of >18,000 CCHD cases among >8 million births provides important information regarding the overall prenatal detection rates and prevalence of CCHD. Some of the findings have profound ethical implications. Some of the feedback is offered with this in mind.<br>Major Issues<br>1. How did the authors define “multiple congenital anomalies that are not related”? I wonder if the authors actually mean that the multiple congenital anomalies do not fit a described pattern. Ockham’s razor suggests that there is likely to be a unifying explanation.<br>2. Can the authors say whether cardiologists or obstetricians diagnosed CCHD at each center? Does it matter?

	3. Figure 2 plots the proportion of prenatally diagnosed and proportion of live births among all CCHD cases by program as a bar graph. (The same data were given in Tables 3 and 5.) The text on p. 8 describes the relationship between the two proportions. The relationship has profound implications regarding why or if a case undergoes TOPFA (cf. Lytzen et al. 2018 JAMA Cardiol 3:828-837). Examination of the reasons is probably beyond the scope of this manuscript, but showing the data as a scatterplot and identifying points that deviate from the trend may help other investigators consider why some regions have higher or lower than expected rates of TOPFA. For example, the Czech Republic and France-Rhone Alps seem to have a lower rate of live births than expected for its rate of prenatal diagnosis. In contrast, Italy-Lombardy appears to have a higher than expected rate of live births. It is not necessarily an Italian phenomenon because Italy-Emilia Romagna and Tuscany fall on the trend line. USA-Atlanta, Canada and Argentina also appear to have higher rates of live births than expected. The trend is real, but the authors should be careful to point out that TOPFA is not strictly related to the prevalence of prenatal CCHD diagnosis. Recognition of the outliers should make one consider what other factors may contribute to the parents' decision. A fuller presentation of the data would help to promote balance in a discussion loaded with ethical issues.<br>(see attached)<br>4. To help others consider why deviations from the expected rate of live birth based on the trend exist, the authors could present the answers to all the questions in the questionnaire about local practices and policies. There may not be enough data to make strong conclusions, but the readers will probably be very interested in the information anyway. Presenting the questionnaire itself as supplementary file could also help guide future investigation. Perhaps the authors already have plans for such a study.<br>Minor Issues<br>1. P. 9, l. 35: Table 3 is referenced, but the data are in Table 2.<br>2. P. 9, l. 21 and 40. Table 2 is referenced, but the data are in Table 3.<br>3. Supplementary Table S1. Does "heterogeneous prevalence" mean that the annual prevalence fluctuates substantially between years? The variation is striking. Do the authors have a quantitative definition of the term? Do the authors have insight into why the variation exists?<br>4. The authors classified CCHDs into several main diagnoses. When a case has multiple defects, they deemed the more "serious" diagnosis to be the main one. They acknowledge that others may disagree with the main diagnosis. I agree that any disagreements would not affect the overall conclusions. That said, the authors could briefly explain their rationale for categories that will draw predictable quibbling. For example, it would seem more logical to make the single ventricle diagnoses that have tricuspid atresia, pulmonary atresia or double outlet right ventricle the latter diagnoses. In that vein, some cases of hypoplastic left heart have "single ventricle" checked are classified as hypoplastic left heart.

**REVIEWER**	Allison Divanovic, MD<br>Cincinnati Children's Hospital Medical Center, Cincinnati, OH, USA
**REVIEW RETURNED**	22-Feb-2019

GENERAL COMMENTS	The authors aim to assess the prevalence of CHD across multiple different sites over time. They also focus on the rates of prenatal diagnosis, type of CHD identified and association with additional anomalies as well as the outcome of the pregnancy and how those percentages differ across various institutions. The strength of this paper is the large number of institutions across multiple countries that contributed data which resulted in the identification of >18,000 cases of CHD. What I find most interesting is the wide range of prenatal detection rates. While I am generally pleased to see a trend toward higher percentages of prenatal diagnoses, I wonder if we can learn anything that is applicable to our own institutions regarding the policies in place at those institutions with the highest prenatal detection rates (France-Rhone Alpes and Italy-Lombardy). I also find the low percentages of still born infants useful information to use when coordinating delivery planning with the obstetricians.<br><br>The only suggestion I have is to include data, if available, regarding gestational age at delivery and birth weight as these factors often influence outcomes. We have made certain observations in our local patient population that makes we wonder if others have noticed the same findings. I also wonder if the authors have data regarding the percentage of parents that chose non-intervention/palliative care and how those numbers differ by location.

REVIEWER	Lazaros Kochilas<br>Emory University and children's Healthcare of Atlanta
REVIEW RETURNED	26-Feb-2019

GENERAL COMMENTS	This is an important and interesting article within the limitations that are appropriately discussed by the authors. I have two comments/suggestions:<br>1. It will be very informative to find out the particular circumstances in some of the reported countries/regions that explain their out of "range" results. For example what is the prenatal protocol used by the the France-Alpes region that makes it so successful? Besides ascertainment what else is behind the large differences between resumably quite similar countries (Czech and Slovak republics)<br>2. Although I agree with most of the authors conclusions I am not sure that such work can help in the prevention of CHD, which in my mind is preventing the pathogenesis of a congenital heart defect in-utero which is different than simply preventing the birth of a child with CHD as it may happen when pregnancies with fetal anomalies are terminated.

VERSION 1 – AUTHOR RESPONSE

Reviewer: 1
Reviewer Name: Andrea Székely
Institution and Country: Semmelweis UNIversity Budapest, Hungary Please state any competing interests or state ‘None declared’: none declared

Please leave your comments for the authors below

The authors presented a retrospective analysis on the detection, types and fate of embryos with congenital heart disease. They have done a huge work also a cross-sectional analysis. The paper is well written and concise. The discussion is also interesting.

>> We thank the reviewer for this positive feedback.

Reviewer: 2
Reviewer Name: Patrick Jay
Institution and Country: Washington University School of Medicine, USA Please state any competing interests or state 'None declared': None declared.

Please leave your comments for the authors below
Bakker et al report retrospective analyses of the prenatal detection of critical congenital heart defects (CCHD), the overall birth prevalence and neonatal (1-month) mortality from 15 international birth defect surveillance programs around the world. Wide variation in practice patterns and other undefined factors were associated with variable prenatal detection rates, termination rates, and 1-month mortality between centers. Some programs contributed considerably more years of data and cases than others over different time periods, which makes comparison between centers challenging. Nevertheless, this cohort of >18,000 CCHD cases among >8 million births provides important information regarding the overall prenatal detection rates and prevalence of CCHD. Some of the findings have profound ethical implications. Some of the feedback is offered with this in mind.
Major Issues

1. How did the authors define "multiple congenital anomalies that are not related"? I wonder if the authors actually mean that the multiple congenital anomalies do not fit a described pattern. Ockham's razor suggests that there is likely to be a unifying explanation.

   >> We thank the reviewer for his positive feedback. This has now been fixed. The definition of MCA used here is one that is used fairly frequently in the literature – that is the co-occurrence of congenital anomalies in the same child that does not constitute a sequence or syndrome (known underlying cause) – but probably this was not explained as clearly as it could have been. We have reworded the section accordingly (page 6). We agree with the reviewer that nearly every co-occurrence is likely to be non-random (as shown statistically by Prof. B Kallen in his work on MCA many years ago). Thank you for pointing this out so we could clarify it.

2. Can the authors say whether cardiologists or obstetricians diagnosed CCHD at each center? Does it matter?
   >> Depending on local practices the diagnosis is being made by an obstetrician or pediatric cardiologist. The programs abstract the clinical information from the medical records, and based on this information the diagnosis is coded and classified by trained staff from the programs. We have added this information to the manuscript, page 5. To improve homogeneity in coding and classification, one of the authors trained in pediatric cardiology and medical genetics (LDB) reviewed manually the case level data transmitted by the contributing programs for those cases with >1 CHD code.
   .

3. Figure 2 plots the proportion of prenatally diagnosed and proportion of live births among all CCHD cases by program as a bar graph. (The same data were given in Tables 3 and 5.) The text on p. 8 describes the relationship between the two proportions. The relationship has profound implications regarding why or if a case undergoes TOPFA (cf. Lytzen et al. 2018 JAMA Cardiol 3:828-837). Examination of the reasons is probably beyond the scope of this manuscript, but showing the data as a scatterplot and identifying points that deviate from the trend may help other investigators consider why some regions have higher or lower than expected rates of TOPFA. For example, the Czech Republic and France-Rhone Alps seem to have a lower rate of live births than expected for its rate of prenatal diagnosis. In contrast, Italy-Lombardy appears to have a higher than expected rate of live births. It is not necessarily an Italian phenomenon because Italy-Emilia Romagna and Tuscany fall on the trend line. USA-Atlanta, Canada and Argentina also appear to have higher rates of live births than expected. The trend is real, but the authors should be careful to point out that TOPFA is not strictly related to the prevalence of prenatal CCHD diagnosis. Recognition of the outliers should make one consider what other factors may contribute to the parents' decision. A fuller

presentation of the data would help to promote balance in a discussion loaded with ethical issues. (see attached)
>> We thank the reviewer for this very useful comment. We agree with the reviewer that the proportion of termination of pregnancy or any other pregnancy outcome is not a direct function of the proportion of prenatal diagnosis and that it is important to acknowledge factors that influence these. We would like to keep the original figure 2, since a scatterplot of proportion prenatally diagnosed and proportion TOPFA may tempt readers to link the two factors directly. We have added a paragraph to the discussion on factors that could influence the observed differences in outcome of pregnancy in relation to prenatal diagnosis (page 15) and added the reference to the manuscript.

4. To help others consider why deviations from the expected rate of live birth based on the trend exist, the authors could present the answers to all the questions in the questionnaire about local practices and policies. There may not be enough data to make strong conclusions, but the readers will probably be very interested in the information anyway. Presenting the questionnaire itself as supplementary file could also help guide future investigation. Perhaps the authors already have plans for such a study.
We have carefully considered the suggestion of the reviewer to include a summary of the answers to the questionnaire as a supplemental table. However, to better understand differences in prenatal detection rate and pregnancy outcomes we also need information on scanning protocols regarding the fetal heart and standards of care, which we regretfully have not. To highlight the reviewer's point, we have extended the paragraph on the prenatal screening policies (page 6) and added a sentence to the Discussion (page 14/15) .

Minor Issues
1. P. 9, l. 35: Table 3 is referenced, but the data are in Table 2.
>> This is now fixed
2. P. 9, l. 21 and 40. Table 2 is referenced, but the data are in Table 3.
>> This is now fixed.
3. Supplementary Table S1. Does "heterogeneous prevalence" mean that the annual prevalence fluctuates substantially between years? The variation is striking. Do the authors have a quantitative definition of the term? Do the authors have insight into why the variation exists?
>> Heterogeneous prevalence means that the prevalence rates fluctuate significantly over the time period examined. The test used was X^2 *test, with* $p<0.05$ *considered statistically significant. The programs in Northern Netherlands and Germany Saxony-Anhalt showed heterogeneous annual prevalence. This may have to do in part with the programs being relatively small, with an average of 17,500 -18,000 annual births.*
4. The authors classified CCHDs into several main diagnoses. When a case has multiple defects, they deemed the more "serious" diagnosis to be the main one. They acknowledge that others may disagree with the main diagnosis. I agree that any disagreements would not affect the overall conclusions. That said, the authors could briefly explain their rationale for categories that will draw predictable quibbling. For example, it would seem more logical to make the single ventricle diagnoses that have tricuspid atresia, pulmonary atresia or double outlet right ventricle the latter diagnoses. In that vein, some cases of hypoplastic left heart have "single ventricle" checked are classified as hypoplastic left heart.
Thank you for this insightful comment. The example of tricuspid plus pulmonary atresia is well taken. To underscore the reviewer's point we have now edited the text in the Appendix by adding a brief discussion of precisely those phenotypes where, in the words of the distinguished reviewer, 'predictable quibbling' may occur. Our hope was that by providing utmost transparency to the process and the findings, readers may more fully understand the ins and outs of the data, including limitations and strengths.

Reviewer: 3
Reviewer Name: Allison Divanovic, MD
Institution and Country: Cincinnati Children's Hospital Medical Center, Cincinnati, OH, USA Please state any competing interests or state 'None declared': None declared

Please leave your comments for the authors below
The authors aim to assess the prevalence of CHD across multiple different sites over time. They also focus on the rates of prenatal diagnosis, type of CHD identified and association with additional anomalies as well as the outcome of the pregnancy and how those percentages differ across various institutions. The strength of this paper is the large number of institutions across multiple countries that contributed data which resulted in the identification of >18,000 cases of CHD. What I find most interesting is the wide range of prenatal detection rates. While I am generally pleased to see a trend toward higher percentages of prenatal diagnoses, I wonder if we can learn anything that is applicable to our own institutions regarding the policies in place at those institutions with the highest prenatal detection rates (France-Rhone Alpes and Italy-Lombardy). I also find the low percentages of still born infants useful information to use when coordinating delivery planning with the obstetricians.
We thank the reviewer for his suggestions. As we have pointed out in the Discussion, prenatal diagnosis is affected by access to prenatal services, availability of technology and skilled sonographers and screening protocols (page 14).

The only suggestion I have is to include data, if available, regarding gestational age at delivery and birth weight as these factors often influence outcomes. We have made certain observations in our local patient population that makes we wonder if others have noticed the same findings. I also wonder if the authors have data regarding the percentage of parents that chose non-intervention/palliative care and how those numbers differ by location.
>> Since our primary aim was to describe trends in prenatal diagnosis and prevalence of critical CHD we did not analyse gestational age and birth weight. We agree with the reviewer that this an interesting and worthwhile topic, and whereas it is outside the scope of this study we are considering additional analyses of these and other variables in the future. We have acknowledged that we did not assess gestational age and birth weight as factors for neonatal mortality in the Discussion (page 16). Regretfully, the surveillance programs collecting the data do not systematically collect information on parents who chose non-intervention or palliative care.

Reviewer: 4
Reviewer Name: Lazaros Kochilas
Institution and Country: Emory University and children's Healthcare of Atlanta Please state any competing interests or state ‘None declared’: None

Please leave your comments for the authors below
This is an important and interesting article within the limitations that are appropriately discussed by the authors. I have two comments/suggestions:

1. It will be very informative to find out the particular circumstances in some of the reported countries/regions that explain their out of "range" results. For example what is the prenatal protocol used by the the France-Alpes region that makes it so successful? Besides ascertainment what else is behind the large differences between resumably quite similar countries (Czech and Slovak republics)
   >> This is an important consideration and we have edited / added text accordingly. Please see our answers to comments 3 and 4 of reviewer 2.

2. Although I agree with most of the authors conclusions I am not sure that such work can help in the prevention of CHD, which in my mind is preventing the pathogenesis of a congenital heart defect in-utero which is different than simply preventing the birth of a child with CHD as it may happen when pregnancies with fetal anomalies are terminated.
   >> We agree completely with the reviewer on the primacy of primary prevention to reduce the impact of these common and often severe conditions. Thank you for pointing out that our final comments could be misconstrued, as we did not mean to imply that prenatal diagnosis and subsequent termination of pregnancy is prevention. Rather we underscore how the high prevalence of CCHD should translate in more active interventions aimed at primary prevention . In addition, prenatal diagnosis can translate into better care of affected newborns, as it allows time for a more deliberate and planned approach to delivery and immediate care. We have changed the sentence in the Discussion to make it more clear (page 17).

VERSION 2 – REVIEW

**REVIEWER**	Patrick Jay<br>Washington University School of Medicine, USA
**REVIEW RETURNED**	16-May-2019

**GENERAL COMMENTS**	The authors have appropriately addressed the issues raised in my original review. Their findings have broad and important implications, which they have thoughtfully considered.

**REVIEWER**	Allison Divanovic<br>Cincinnati Children's Hospital, Cincinnati, OH, USA
**REVIEW RETURNED**	24-May-2019

**GENERAL COMMENTS**	The authors have revised the manuscript in a way that has adequately addressed the suggestions of the reviewers.

**REVIEWER**	Lazaros Kochilas<br>Emory University and Children's Healthcare of Atlanta, USA
**REVIEW RETURNED**	28-May-2019

**GENERAL COMMENTS**	The authors appropriately addressed the reviewers' comments within the limitations of this study, which were adequately presented in the revised manuscript.
